# Consensus recommendations for the nutritional management of children with cancer in limited resource settings: a report from the International Initiative for Pediatrics and Nutrition

**DOI:** 10.3389/fnut.2025.1605632

**Published:** 2025-06-26

**Authors:** Karina Viani, Jullyana Alves, Erika Damasco-Avila, Mariana S. Murra, Judy Schoeman, Michelle Walters, Elena J. Ladas

**Affiliations:** ^1^Department of Pediatrics, School of Medicine, Instituto de Tratamento do Câncer Infantil, University of São Paulo, São Paulo, Brazil; ^2^Department of Pediatrics, Instituto de Medicina Integral Prof. Fernando Figueira, Recife, Brazil; ^3^Division of Hematology/Oncology/Stem Cell Transplantation, Department of Pediatrics, Columbia University Irving Medical Center, New York, NY, United States; ^4^Department of Pediatrics, Hospital de Amor, Barretos, Brazil; ^5^Division of Paediatric Oncology and Haematology, Department of Paediatrics and Child Health, Faculty of Medicine and Health Sciences, University of Pretoria, Pretoria, South Africa; ^6^Department of Paediatrics and Child Health, Faculty of Medicine and Health Sciences, Tygerberg Hospital, Stellenbosch University, Cape Town, South Africa

**Keywords:** manuals, neoplasms, pediatrics, malnutrition, nutrition assessment, diet therapy, nutritional support

## Abstract

**Introduction:**

Malnutrition (under- and over-nutrition) is a critical challenge in pediatric oncology, particularly in low- and middle-income countries (LMIC), where supportive care resources are scarce. It negatively impacts treatment toxicity, survival rates, and quality of life. Despite the availability of broad guidelines, there is a lack of practical, context-adapted protocols for nutritional assessment and intervention in LMIC. The International Initiative for Pediatrics and Nutrition (IIPAN) developed adapted consensus-based protocols to address this gap.

**Methods:**

A multidisciplinary panel of experts in pediatric oncology and nutrition from high-income and LMIC settings collaborated to develop adapted nutrition assessment and intervention manuals. The process involved literature reviews, iterative expert consultations, field testing in LMIC hospitals, and subsequent revisions based on real-world application feedback. Training programs were designed to ensure implementation, including in person and virtual mentorship.

**Results:**

Two comprehensive manuals and one complementary material were developed: (1) Nutritional Assessment: A Training Manual in Anthropometry, (2) Nutritional Intervention: A Training Manual for Pediatric Oncology, and (3) Appendix for Nutritional Intervention. These resources provide structured detailed guidance on nutrition assessment, interventions, and the management of nutrition-related complications. Their scalability and accessibility are crucial for optimizing nutritional management and improving clinical outcomes in limited resource settings.

**Conclusion:**

These evidence-based, expert-designed adapted protocols address critical gaps in nutritional care for children with cancer in LMIC.

## 1 Introduction

The global burden of childhood cancer is projected to be 13.7 million new cases between 2020 and 2050 (1), with the majority (90%) occurring in low- and middle-income countries (LMIC) (2). Unfortunately, significant disparities in survival exist between children receiving treatment in the high-income setting compared to LMIC (1, 2). One of the main factors adversely affecting the survival of children with cancer undergoing treatment in an LMIC is the scarcity of well-established supportive care programs, with nutrition representing the most notable area in need of investment (1). This disproportionally affects children with cancer in LMIC due to the endemic burden of malnutrition (under- and over-nutrition) alongside widespread prevalence of micronutrient deficiencies (3–6).

The prevalence of undernutrition at diagnosis among children with cancer in LMIC varies by region with up to 75% of children with cancer also classified as undernourished at diagnosis (7, 8). Undernutrition often increases over the course of treatment, particularly for children receiving highly intensive chemotherapy. At the same time, an increasing burden of overnutrition is facing many LMIC. Poor nutrition is associated with higher rates of treatment-related toxicity (9, 10), increased mortality (9–14), treatment abandonment (9, 15), and poorer quality of life (16). Managing poor nutrition in LMIC is especially challenging due to limited access to nutritional support, lack of trained personnel or nutrition specialists, high personnel turnover, and sociodemographic factors (e.g., food insecurity, low socioeconomic status) which hinder the initiation and effectiveness of nutritional interventions in LMIC (17–19).

The development of adapted protocols for the delivery of nutritional care for children with cancer in countries with limited resources is essential for improving their clinical outcomes. Adapted guidelines have been put forth in establishing nutrition programs for pediatric cancer units in LMIC (20); however, there are no available adapted care protocols for the delivery of nutrition assessment and intervention. The International Initiative for Pediatrics and Nutrition (IIPAN), established in 2016 and based at Columbia University Irving Medical Center, is a global initiative that aims to build clinical nutrition capacity and advance nutrition research in LMIC. IIPAN supports and trains local clinical research nutritionists at each collaborating center, providing them with specialized training in nutritional screening, assessment, intervention, and monitoring (7, 21). Recognizing the need for comprehensive clinical adapted care protocols, we present consensus-based protocols for nutrition assessment and intervention for children with cancer undergoing treatment in limited-resource settings.

## 2 Methods

The methodology employed a modified Delphi technique to reach a consensus among the expert panel, which consisted of nutritionists, dieticians, and pediatric oncologists each with extensive experience working with children with cancer in limited-resource settings. Development of the initial protocols began in 2018 and were completed in 2019. The nutrition protocols then underwent pilot testing and were revised in 2022 based on user feedback. Throughout the development process, stakeholder meetings were held where panel members reviewed the content based on their collective insights. Panel members provided feedback over multiple rounds, which led to subsequent revisions aimed at enhancing the clarity and relevance of the content. Final consensus was achieved through collaborative discussions, resulting in agreement on the revised drafts.

The content of the manual was selected based on the anthropometric, biochemical, clinical and dietary indicators forming nutrition assessment and driving the form of subsequent nutrition intervention. The guidelines were designed to provide guidance in managing factors common in resource-limited settings such as children presenting with high tumor burden, limited access to nutrition support products and necessary adaptation of severe acute malnutrition (SAM) guidelines are all commonly encountered clinical conditions in the LMIC setting. Following this, a literature review was performed to evaluate existing guidelines, best practices, and peer-reviewed clinical studies in childhood cancer for each topic. A report was generated and through an iterative process the available resources were reviewed with the panel members for determination of inclusion/exclusion based on applicability in the resource-limited setting.

The process resulted in three training manuals:

• Nutritional Assessment: A Training Manual in Anthropometry (Supplementary file 1): This manual provides detailed instructions for the accurate collection of anthropometric data and is designed to facilitate the gathering of nutritional indicators crucial for assessing growth in children and adolescents with cancer.

• Nutritional Intervention: A Training Manual for Pediatric Oncology (Supplementary file 2): This manual outlines nutritional intervention strategies aimed at sustaining and promoting normal growth and development in children undergoing cancer therapy.

• Appendix for Nutritional Intervention: A Training Manual for Pediatric Oncology (Supplementary file 3): This manual serves as an appendix to the nutrition intervention manual, providing necessary supplemental information on biochemical, clinical and dietary data to conduct a comprehensive nutrition assessment and intervention.

## 3 Results

### 3.1 Training and implementation

Healthcare professionals representing several disciplines, including dietitians/nutritionists, physicians, and nurses, were trained in the best practices outlined in the protocols. Trainees underwent several training phases, which consisted of a week-long intensive program conducted through group or individual instruction. Following the initial training, ongoing mentorship was provided through virtual group calls. Onsite training was provided annually, and monitoring and evaluation was performed on a quarterly basis.

Before each initial training session, a survey based on the framework proposed by Ladas et al. (20) was conducted to evaluate the hospital’s nutrition resources, personnel, and services. This survey helps customize the training to address the available resources of each hospital based on the International Society of Paediatric Oncology (SIOP) nutrition assessment of the unit’s level of care. The manuals were provided to all trainees before the initial training phase in preparation for the training sessions.

Group instruction included a combination of lectures and clinical rotations. The lecture content was developed to complement the materials in the manuals, incorporating active learning techniques that foster a deeper understanding of the material and encourage critical thinking. Active learning methods included group case studies, role-playing activities, and discussions. After the lectures, participants attended clinical rotations at local hospitals, where they gained hands-on experience providing nutritional care as part of a multidisciplinary healthcare team. Experienced dietitians supervised these rotations and offered guidance and feedback (22). The manuals were referenced and utilized throughout the lectures and clinical rotations.

A pre-test and post-test knowledge assessment was administered to compare participants’ knowledge before and after the training, allowing for the measurement of knowledge gained. After the training, participants completed an evaluation to provide feedback on various aspects of the course, such as the clarity of the content and instructors, the interactivity of the course, and the relevance of the material. This feedback was used to improve future versions of the course (22).

Individual training has also been implemented. During this training, a designated IIPAN trainer conducted on-site sessions for the established IIPAN program. The IIPAN nutritionists received training in nutrition screening, assessment, interventions, and monitoring while providing ongoing nutritional care to patients. This training included program setup and implementation, participation in ward rounds, and the establishment of procedures for communication with the healthcare team.

To provide ongoing support and mentorship, weekly virtual calls were held to discuss clinical nutrition cases. These calls promote a supportive environment focused on improving patient care and nutritional competency of the nutritionist. Each session included representatives from multiple hospitals fostering peer-to-peer learning. An IIPAN trainer led these calls; however, all participants were encouraged to engage in discussions and provide feedback, creating an interactive learning atmosphere. The IIPAN trainer referenced manuals during the calls to ensure standardized nutritional care. As the nutritionists demonstrated increased competency, the frequency of the training calls gradually decreased.

### 3.2 Nutritional assessment

Evaluating nutritional status is essential throughout the continuum of cancer care to ensure optimal growth and development while maximizing clinical outcomes. Nutritional assessment enables clinicians to monitor growth, identify any growth issues, and determine appropriate nutritional interventions. The Nutritional Assessment protocols provide comprehensive guidelines for conducting nutritional anthropometrics in the pediatric oncology population, ensuring accurate and consistent collection of anthropometric data. These protocols include detailed instructions on how to measure, plot, and interpret weight, height, Body Mass Index (BMI)-for-age, and mid-upper arm circumference (MUAC) accurately. Additionally, the manual offers guidance on identifying potential growth problems and diagnosing malnutrition based on BMI-for-age and MUAC. It also highlights specific circumstances when MUAC should be considered over BMI-for-age.

For most countries, the World Health Organization’s (WHO’s) growth charts for height-for-age, BMI-for-age, and MUAC-for-age are included in the manuals (23, 24). For children older than 5 years, the manual also includes Mramba’s MUAC growth charts (25). In select cases, country-specific cut-offs serve as the benchmark to monitor growth and development. To expedite the time spent performing nutrition assessment, applications that assist with calculating z-score are provided.

Some adaptations were incorporated to ensure the proper collection of anthropometric data in various clinical settings, capacities, and levels of resources, and considered the specific needs of children and adolescents with cancer. The manual adapted instructions for measuring weight and height in children with difficulty standing or having amputations, ensuring accurate measurements despite these challenges. Additionally, it provides guidance for using computer software and cellphone applications to facilitate the calculation and interpretation of growth indicators. Other adaptations include guidance on the use of the tared weighing method, adjusted weight calculations for children with amputations, measuring length in a supine position with a tape measure in the absence of a length board, and providing a BMI reference table when a calculator is not available.

### 3.3 Nutritional intervention

Nutritional interventions are often challenging to implement in a child with cancer due to the multiple factors concurrently impacting appetite and dietary intake. The Nutrition Intervention manual is a comprehensive resource designed to guide nutritionists utilizing data obtained from the methodology outlined in the Nutrition Assessment manual to guide the development of a tailored nutrition intervention. The manual covers the types of pediatric cancers (acute leukemias, lymphomas, brain tumors, neuroblastoma, Wilms tumor, soft tissue sarcomas of skeletal muscular origin and bone tumors) and their treatments, along with common gastrointestinal complications resulting from these treatments (bowel perforation and/or obstruction, constipation, diarrhea, gastrointestinal hemorrhage, mucositis/stomatitis, nausea and vomiting, neutropenic enterocolitis/typhlitis, pancreatitis, acute kidney injury). Next, the manual provides an overview of how to perform a nutrition assessment, including the evaluation of biochemical parameters to aid the detection and remediation of macro- and micronutrient deficiencies as well as how to monitor nutrition-related toxicities such as hypertriglyceridemia or hyperglycemia, clinical examinations and screening for signs of severe vitamin and trace element deficiency, both in table format with relevant pictures for ease of reference. In-depth dietary assessments, including information on nutrition requirements and food records, and methods in conducting a 24-h recall are provided. The Intervention Manual provides guidance on implementing adapted nutrition interventions (e.g., specialized diets including food safety diet, low-sodium diet, low-fat diet) and strategies for nutritional counseling, enteral tube feeding (types of feeding tubes, placement of the nasogastric [NG] tube, cleaning the NG tube, determining the formula, dosing/rate/duration), parenteral nutrition, and criteria for monitoring a nutritional intervention. A significant section is dedicated to managing SAM in children diagnosed with cancer, with modifications based on clinical experience for treating SAM in this population, recognizing that typical signs and symptoms may not always be present.

Accompanying this manual, the “Appendix for Nutrition Intervention: A Training Manual for Pediatric Oncology” contains several tables of relevant complementary information, such as management of common nutrition-related side-effects of cancer chemotherapy and radiotherapy, common laboratory tests used in nutritional assessment, clinical evidence of nutritional deficiencies, and reference energy, protein, fluids and micronutrient requirements. Additionally, the Appendix contains validated algorithms (26). Lastly, the Appendix lists commonly used enteral formulas in limited-resource settings.

Adaptations were made to facilitate the provision of nutritional intervention across diverse clinical settings, resource levels, and capacities. These include the consideration of culturally driven food choices and socioeconomic status, emphasis on the use of clinical examinations and dietary recalls when advanced biochemical tests are unavailable, modification of oral and enteral interventions to incorporate locally available foods and homemade feeds when commercial formulas are unavailable (recipes from different pediatric cancer units were compiled), local guidance on food safety, alternative methods for verifying NG tube placement; guidance on gravity or bolus feeding in the absence of diet pumps, alternative approaches when there is a lack of standard or individualized parenteral nutrition solutions, and recommendations for using ready-to-use therapeutic food (RUTF) for children with SAM when supplies of F-100 are limited. These adaptations ensure that the protocol is practical and applicable to the diversity of resources in LMIC settings.

## 4 Discussion

The objective of the IIPAN consensus-based protocols is to serve as a guide in the nutritional management of children with cancer. Therefore, it is important to consider their scaling and distribution to ensure that they reach the regions where they are most needed. This involves partnerships with non-governmental organizations and health organizations for distributing the manuals. One partnership is that with the WHO Knowledge Action Portal (KAP) where IIPAN is a collaborating partner with WHO. This platform expedites the sharing of knowledge and builds global communities and fosters collaboration. Through this partnership, IIPAN will share educational materials to promote knowledge exchange and share best practices in building capacity in the nutritional workforce. Providing access to these manuals and other materials in the KAP will be an invaluable resource for healthcare professionals working in pediatric oncology. To ensure access to clinicians whose English is their second language, translation of these manuals to several languages is underway.

Dissemination of these manuals also occurs through routine societal meetings. The International Society of Paediatric Oncology (SIOP) Nutrition Network has collaborated with IIPAN and has hosted nutrition workshops at the regional SIOP Africa and Asia congresses since 2014. In collaboration with IIPAN, SIOP Africa and World Child Cancer (WCC), country-wide trainings have taken place in Uganda and Ghana (22, 27). At the five-day nutritional workshop held by WCC and IIPAN in Accra, Ghana in 2022, 74% of participants felt they were ‘very knowledgeable’ after the course compared to 10% prior and 92% felt they could use the knowledge daily (22). Additionally, there is ongoing collaboration between IIPAN and regional oncology societies including the Pediatric Oncology East and Mediterranean Group, the Pediatric Hematology-Oncology Association of Central America – which consists of pediatric cancer units located in Central America and Caribbean – and the Sociedade Brasileira de Oncologia Pediátrica (Pediatric Oncology Brazilian Society) which hosts nutrition sessions at biannual meetings to advance clinical practice (28). The success of these meetings have been instrumental in advancing knowledge and care with 33 meetings held to date on nutrition and childhood cancer resulting in 5,615 clinicians educated to date (27, 29, 30).

In 2022, the Pan American Health Organization (PAHO) published the *Nutritional Care Guide for Pediatric Cancer* (31). This guide was developed within the framework of the World Health Organization’s Global Initiative for Childhood Cancer, with the main objective of addressing the nutritional care for children with cancer. The guide is available in Spanish, English, and French and represents experts from the Americas, whereas the IIPAN protocols were designed by a global panel of experts and tested in multiple settings, including sites in Africa and Asia, ensuring broader applicability across diverse healthcare contexts. These distinctions highlight the complementary nature of both resources, with the PAHO guidelines serving as a broad framework and the IIPAN manuals offering practical, field-tested guidance tailored to clinical use in resource-limited settings. Notably, there are also local manuals and guidelines for the nutritional care of pediatric cancer, such as Brazil’s national cancer institute consensus on pediatric oncology nutrition (32). Although tailored to the culture of a specific country, these are often limited by language and developed by local experts.

There are some limitations to our work. The English language is a barrier that may restrict accessibility and implementation in some regions, thus translation in other languages is ongoing. The limited availability of studies on nutrition in pediatric oncology, particularly in LMIC, poses challenges in establishing comprehensive, evidence-based recommendations tailored to these settings, although this is rapidly changing with the development of multi-national research groups. IIPAN and the International Agency for Research on Cancer/WHO, have formalized a collaboration to conduct two multinational nutrition biobanking studies. The first study is recruiting children and adolescents receiving treatment for acute lymphoblastic leukemia (ALL) or brain tumors in Southern Europe (clinicaltrials.gov ID NCT05375617), while the second study is recruiting children and adolescents undergoing treatment for ALL in LMICs (clinicaltrials.gov ID NCT05929976). Our adapted protocols also have notable strengths. They were developed by experts from multiple countries, ensuring a diverse and globally informed perspective. Moreover, they are specifically designed to be practical and adaptable for resource-limited settings, enhancing their applicability in diverse healthcare infrastructures, and were tested in real-world scenarios by local dietitians/nutritionists and refined prior to finalization. An ongoing commitment to continuous monitoring and updates, integrating user feedback and adjusting recommendations as capacity and resources evolve as well as new scientific evidence becomes available ensures the long-term relevance and sustainability of these adapted protocols.

## 5 Conclusion

This effort is a significant step toward improving the nutritional management of children with cancer, particularly in LMIC. By providing evidence-based, expert-designed recommendations tailored to resource-constrained settings, these protocols have the potential to enhance early identification of nutritional risks, promote timely interventions, and ultimately improve patient outcomes, which aligns with the WHO’s Global Initiative for Childhood Cancer goal of increasing the survival rate of children with cancer by 2030 (33). Furthermore, the adaptability of these protocols to different healthcare infrastructures increases their application to diverse clinical settings, fostering a more equitable nutrition care to pediatric cancer patients worldwide.
