# Correction: Consensus recommendations for the nutritional management of children with cancer in limited resource settings: a report from the International Initiative for Pediatrics and Nutrition

**DOI:** 10.3389/fnut.2025.1675916

**Published:** 2025-08-22

**Authors:** Karina Viani, Jullyana Alves, Erika Damasco-Avila, Mariana S. Murra, Judy Schoeman, Michelle Walters, Elena J. Ladas

**Affiliations:** ^1^Department of Pediatrics, School of Medicine, Instituto de Tratamento do Câncer Infantil, University of São Paulo, São Paulo, Brazil; ^2^Department of Pediatrics, Instituto de Medicina Integral Prof. Fernando Figueira, Recife, Brazil; ^3^Division of Hematology/Oncology/Stem Cell Transplantation, Department of Pediatrics, Columbia University Irving Medical Center, New York, NY, United States; ^4^Department of Pediatrics, Hospital de Amor, Barretos, Brazil; ^5^Division of Paediatric Oncology and Haematology, Department of Paediatrics and Child Health, Faculty of Medicine and Health Sciences, University of Pretoria, Pretoria, South Africa; ^6^Department of Paediatrics and Child Health, Faculty of Medicine and Health Sciences, Tygerberg Hospital, Stellenbosch University, Cape Town, South Africa

**Keywords:** manuals, neoplasms, pediatrics, malnutrition, nutrition assessment, diet therapy, nutritional support

In the published article, there was an error in the **Supplementary File 1**, *Table 1*. A column was missing to differ the nutritional classifications for overweight and obesity by age group. The correct Supplementary File 1 has been published in the original article.

The original article has been updated.

