# Supplementary material for: Consensus recommendations for the nutritional management of children with cancer in limited resource settings: a report from the International Initiative for Pediatrics and Nutrition
Source: Front Nutr. 2025 Jun 26;12:1605632. doi: 10.3389/fnut.2025.1605632 (PMC12240747; doi:10.3389/fnut.2025.1605632)
Supplement: Supplementary file 1 [file Data_Sheet_1.pdf]

IIPAN

COLUMBIA
COLUMBIA UNIVERSITY
IRVING MEDICAL CENTER

IIPAN
INTERNATIONAL INITIATIVE
for PEDIATRICS AND NUTRITION

Nutritional Assessment:
A Training Manual in Anthropometry

Second Edition

Contents

Introduction 4

Measuring Weight and Height 4

Measuring Weight 4

Calculating Weight for Children with Amputations 6

Measuring Height 6

Measuring Height with a Length Board 6

Measuring Height with Stadiometer 7

Body Mass Index (BMI), Percentile, and Z-score 8

Plotting and Interpreting BMI 9

Plotting BMI-for-age 9

Computer Software 10

Cellphone Applications 10

WHO Growth Charts 10

Interpret Plotted Points for Growth Indicators 11

Identify Growth Problems from Plotted Points 12

Trends in BMI-for-age 13

Mid-upper Arm Circumference (MUAC) 14

MUAC Measurement 14

Measuring Tapes 14

Obtaining the Measurement 15

Classification of Malnutrition 15

MUAC Indicators of Overweight and Obesity 16

Frequency of Anthropometric Monitoring 16

References 17

Additional Reading 17

Appendix A: BMI Tables 19

Table A-1. BMI for Corresponding Height (cm) and Weight (kg) 19

Table A-2. BMI for Corresponding Height (cm) and Weight (kg) 20

Appendix B: Height-for-age and BMI-for-age Growth Charts 21

Figure B-1: Length/height-for-age Percentiles for Girls - Birth to 5 Years 21

Figure B-2: Length/height-for-age Z-scores for Girls - Birth to 5 Years 21

Figure B-3: Length/height-for-age Percentiles for Boys - Birth to 5 Years 22
Figure B-4: Length/height-for-age Z-scores for Boys - Birth to 5 Years 22
Figure B-5: Height-for-age Percentiles for Girls - 5 to 19 Years 23
Figure B-6: Height-for-age Z-scores for Girls - 5 to 19 Years 23
Figure B-7: Height-for-age Percentiles for Boys - 5 to 19 Years 24
Figure B-8: Height-for-age Z-scores for Boys - 5 to 19 Years 24
Figure B-9. BMI-for-age Percentiles for Girls - Birth to 5 Years 25
Figure B-10. BMI-for-age Z-scores for Girls - Birth to 5 Years 25
Figure B-11. BMI-for-age Percentiles for Boys - Birth to 5 Years 26
Figure B-12. BMI-for-age Z-scores for Boys - Birth to 5 Years 26
Figure B-13. BMI-for-age Percentiles for Girls - 5 to 19 Years 27
Figure B-14. BMI-for-age Z-scores for Girls - 5 to 19 Years 27
Figure B-15. BMI-for-age Percentiles for Boys - 5 to 19 Years 28
Figure B-16. BMI-for-age Z-scores for Boys - 5 to 19 Years 28
Appendix C: MUAC-for-age Growth Charts 29
Figure C-1. MUAC-for-age Percentiles for Girls - 3 Months to 5 Years 29
Figure C-2. MUAC-for-age Z-scores for Girls - 3 Months to 5 Years 29
Figure C-3. MUAC-for-age Percentiles for Boys - 3 Months to 5 Years 30
Figure C-4. MUAC-for-age Z-scores for Boys - 3 Months to 5 Years 30
Figure C-5. MUAC-for-age Z-scores for Girls - 5 to 19 Years 31
Figure C-6. MUAC-for-age Z-scores for Boys - 5 to 19 Years 31

Abbreviations

BMI	body mass index	mm	millimeter
CDC	Center for Disease Control and Prevention	MUAC	mid-upper arm circumference
cm	centimeter	UNICEF	United Nations Children's Fund
kg	kilogram	WHO	World Health Organization
m	meter		

Introduction

Evaluation of nutritional status is necessary throughout the continuum of cancer care to ensure normal growth and development and optimize clinical outcomes. Nutritional assessment enables clinicians to monitor growth and development, identify growth problems, and determine nutritional interventions. Nutritional assessment includes anthropometry (*measurements obtained to evaluate the size, shape, and composition of the human body*), diagnostic testing including general and nutrition-specific biochemical tests, a general physical examination to detect nutrient deficiencies, and analysis of dietary intake. Advanced imaging may be utilized for evaluation of body composition. In pediatric oncology, consideration of the anticipated cancer treatment and expected acute- and long-term toxicities are routinely included in a nutritional assessment.

<u>This manual provides instruction on the proper collection of anthropometric data</u>. This training manual is designed to facilitate the collection of nutritional indicators of growth assessment in children and adolescents with cancer. Additional reading on nutritional assessment may be found in the references. Instructions for the collection of nutritional anthropometrics have been adapted from the World Health Organization (WHO), United Nations Children's Fund (UNICEF), and Center for Disease Control and Prevention (CDC) training modules and guidelines.[1-3] References within each section indicate the primary resource.

The intended audience of this manual is the healthcare team working in the clinical setting, both in- and out-patient. Included in this manual are reference growth charts provided by international agencies, although it is recognized that some regions may have region-specific growth charts. We recommend consulting with the leadership at your institution to comply with your institutional standards of practice. Instruction on calculations related to nutritional assessment along with graphing and interpretation is provided. When applicable, text is supported with images to provide visual instruction.

Measuring Weight and Height

The following supplies are recommended to measure weight and height:

- Scale
- Height board (stadiometer) or measuring tape
- Length board (infantometer) or measuring tape
- Growth curves
- Calculator, computer program (Anthro and AnthroPlus), and/or phone application (AnthroCalc)

Measuring Weight

Source: WHO[1]

Many different kinds of scales can be used for measuring weight. Scales with attached stadiometers (*devices for measuring height*) are ideal for the clinical setting as they readily provide both weight and height. A weight scale without a stadiometer is ideal for use in a variety of locations due to its ease of transportation. The WHO recommends that the scale be solid and durable, with digital reading,

measuring up to 150 kilograms (kg) to a precision of 0.1 kg, and allow for tared weighing.[1] Bathroom and hanging scales are not reliable for obtaining weight measurements.

Taring a scale (*tared weighing*) is a methodology used to help weigh small children. Taring means setting the scale to zero with an individual standing on the scale. Electronic scales can be tared with the parent/caregiver's weight and then the child is held by the parent/caregiver to weigh the child. Taring reduces the risk of arithmetic errors. Scales that are not digital are not able to be tared.

Taring is ideal for children who are less than two years old or are unable to stand. The following steps are recommended for tared weighing:[1]

- The parent/caregiver's weight is obtained.
- When the weight appears on the scale, tare the scale – or set it to zero – while the parent/caregiver is still standing.
- Ask the parent/caregiver to hold the child.
- Record the new weight on the scale as the weight of the child.

In cases where the parent/caregiver is heavy (>100 kg) and the child's weight is too low (<2.5 kg) to register on the scale, have a lighter person hold the baby on the scale and re-tare the scale to record the child's weight. Refer to Figure 1 for an illustration of tared weighing.

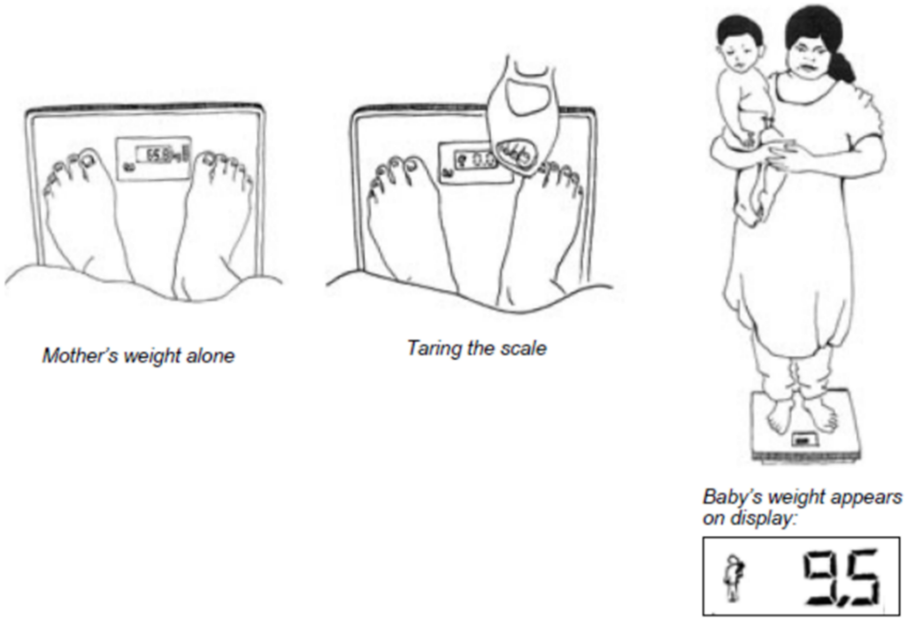

Figure 1. Tared weight (Source: WHO[1])

Institutions that do not have a tared scale will have to weigh the caregiver first and document that weight. Have the caregiver hold the child and then weigh again with the caregiver and child; the weight of the caregiver must be subtracted from the total weight to get the weight of the child.

Children older than two years of age should be weighed alone on the scale unless clinical circumstances prevent the child from standing alone.

Scales can lose accuracy over time due to general use, frequent movement, and environmental conditions; therefore, it is important to ensure that scales are calibrated (*weighing accurately*).[4] To test whether a scale is accurately calibrated, weigh an object with a known weight and check whether the

scale accurately measures the object. If recalibration is needed, the method will depend on the type of scale and model. Testing for calibration should be performed every three months.

Calculating Weight for Children with Amputations

For children and adolescents with an amputation, the following formula may be used to calculate weight while adjusting for the amputation (Figure 2).[5]

$$\text{Corrected weight for an amputee} = \frac{(\text{Current weight} \times 100)}{(100 - \%\ \text{Amputation})}$$

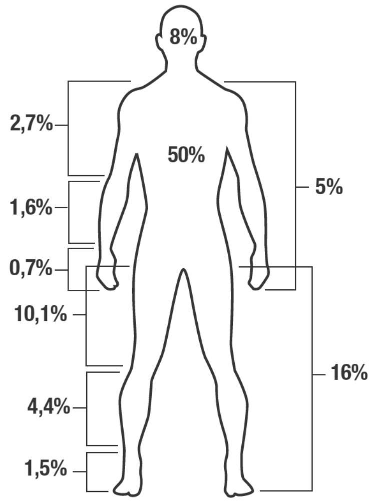

Figure 2. Percent Amputation (Source: Sociedade Brasileira de Oncologia Pediátrica[6])

Case Example: Calculate Weight for an Amputee

12-year-old male with left leg amputation

Current weight: 50 kg

Proportion of weight due to full leg amputation: 16% (Figure 2)

$$\text{Corrected weight} = \frac{(50\ \text{kg} \times 100)}{(100 - 16)}$$

$$\text{Corrected weight} = \frac{5000}{84}$$

Answer: Corrected weight = 59.5 kg

Measuring Height

Depending on a child's age and ability to stand, height may be measured lying down or standing upright.

Measuring Height with a Length Board

The WHO recommends the following steps using the length board to measure the child's height lying down (Figure 3).[1] Cover the board with cloth or paper for hygiene. Lay the child on their back with their

head against the fixed headboard. While holding the knees, pull the footboard against the child's feet. The soles of the feet should be flat against the footboard, toes pointing upwards. Read the measurement and record the child's length in centimeters (cm) to the last completed 0.1 cm.

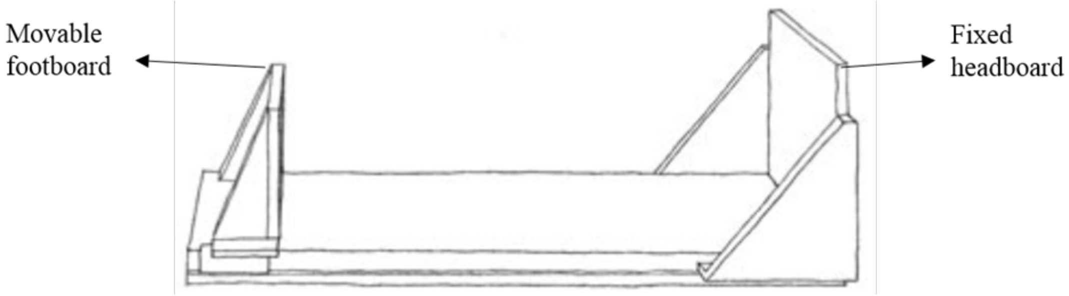

Figure 3. Length board (Source: WHO[1])

If no length board is available, place the child in a supine position (*lying flat horizontally on their back*) on a paper sheet that can be marked with a pen. Mark the sheet on the top of the head end and the base of the foot (*feet should be flexed upward*). Measure the distance between marks with a tape measure.

If paper sheets are not available, measure bed height. Bed height is measured on a flat bed and with pillows removed. The patient lays horizontal in a bed with feet flexed upward. Use a clipboard as a flat surface to extend perpendicular lines from the top of the head and the bottom of the feet. Then, mark the sheet at two positions close to the side edge of the bed. Measure the distance between the two marks using a flexible tape to the nearest 0.5 cm.[7]

Measuring Height with Stadiometer

To measure height with a stadiometer follow the instructions recommended by the WHO[1]:

- Before measuring ensure that the stadiometer is on the ground level and the child has removed shoes, socks, and any hat/hair ornaments.
- The child should stand with flat feet with the back of the head against the vertical board. The back of the head, shoulder blades, back, calves, and heels should all touch the vertical board of the stadiometer.
- Ensure that the child's head is positioned horizontally – eyes are parallel to the baseboard.
- After aligning the child's body, slide down the headboard to rest firmly on top of the head.
- Make sure the measurer's eyes are at the same level as the headboard.
- Read and record the measurement after the patient exhales, if possible.
- Accurately record the height to the nearest 0.1 cm.

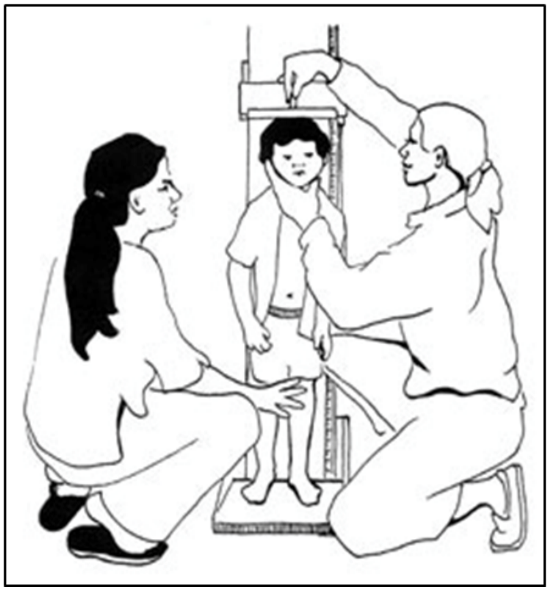

Figure 4. Measuring height with a stadiometer (Source: WHO[1])

Body Mass Index (BMI), Percentile, and Z-score

BMI is calculated from height and weight. It is a growth indicator that can be plotted on a graph by age and sex to measure growth and identify problems.

$$\text{BMI}\left(\text{kg/m}^2\right) = \frac{\text{Weight (kg)}}{\text{Length or Height (m)}^2}$$

Use metric units for BMI calculations and round to one decimal place.

Metric units:

1 inch = 2.54 cm = 0.0254 m

1 pound = 0.4536 kg

To calculate BMI using a calculator with x^2 function:[1]

- Type in the weight in kg (*to the nearest 0.1 kg*).
- Press the / or ÷ sign.
- Type in the length or height in meters.
- Press the x^2 button. The height squared is displayed.
- Press the = button. The BMI is displayed.
- Round the BMI to one decimal place and record the BMI.

In a calculator with no x^2 button, follow steps 1–3, and then press the = button 2 times to display the BMI.

If you have no calculator, utilize the BMI table (Appendix A).[1] The WHO instructions are as follows:

- Height or length is listed (*centimeters)* on the far left column of the BMI table. Select the closest measurement - if height is halfway select the next higher measurement.

- Weight is listed across the row. Select the closest height - if the weight is halfway between those shown, consider it “on the line”.
- The corresponding BMI would be directly upward – on the top row of the table (*also listed on the bottom row*). If the weight was “on the line”, the BMI will be halfway between those shown, e.g., 14.5 if between 14 and 15.
- Record the BMI.

Case Example: Determine BMI with the BMI Table

Source: WHO[1]

2 years and 4 months old female

Height is 88.2 cm. The closest height in the far left column of the table is 88 cm *(circled below*).

Weight is 11.5 kg. The closest weight on the row for her height is 11.6 kg.

Tracing a finger upward from the weight (*follow the lined arrow*), you find that her BMI (*on the top row of the table*) is 15.

L or H (cm)	Body Mass Index (BMI)																			L or H (cm)
	8	9	10	11	12	13	14	15	16	17	18	19	20	21	22	23	24	25	26	
84	5.6	6.4	7.1	7.8	8.5	9.2	9.9	10.6	11.3	12.0	12.7	13.4	14.1	14.8	15.5	16.2	16.9	17.6	18.3	84
85	5.8	6.5	7.2	7.9	8.7	9.4	10.1	10.8	11.6	12.3	13.0	13.7	14.5	15.2	15.9	16.6	17.3	18.1	18.8	85
86	5.9	6.7	7.4	8.1	8.9	9.6	10.4	11.1	11.8	12.6	13.3	14.1	14.8	15.5	16.3	17.0	17.8	18.5	19.2	86
87	6.1	6.8	7.6	8.3	9.1	9.8	10.6	11.4	12.1	12.9	13.6	14.4	15.1	15.9	16.7	17.4	18.2	18.9	19.7	87
88	6.2	7.0	7.7	8.5	9.3	10.1	10.8	11.6	12.4	13.2	13.9	14.7	15.5	16.3	17.0	17.8	18.6	19.4	20.1	88
89	6.3	7.1	7.9	8.7	9.5	10.3	11.1	11.9	12.7	13.5	14.3	15.0	15.8	16.6	17.4	18.2	19.0	19.8	20.6	89
90	6.5	7.3	8.1	8.9	9.7	10.5	11.3	12.2	13.0	13.8	14.6	15.4	16.2	17.0	17.8	18.6	19.4	20.3	21.1	90
91	6.6	7.5	8.3	9.1	9.9	10.8	11.6	12.4	13.2	14.1	14.9	15.7	16.6	17.4	18.2	19.0	19.9	20.7	21.5	91
92	6.8	7.6	8.5	9.3	10.2	11.0	11.8	12.7	13.5	14.4	15.2	16.1	16.9	17.8	18.6	19.5	20.3	21.2	22.0	92

To use the formula (kg/m^2) and a calculator to determine BMI, convert height in meters. The height of 88.2 cm is 0.882 m. The girl’s BMI is calculated as follows:

$$\text{BMI}\left(\text{kg/m}^2\right) = \frac{\text{Weight (kg)}}{\text{Length or Height (m)}^2}$$

$$\text{BMI}\left(\text{kg/m}^2\right) = \frac{11.5\ \text{kg}}{0.882\ \text{m}^2} = 14.8\ \text{kg/m}^2$$

This would be recorded as a BMI of 14.8 kg/m^2. This number will be used to determine z-score on the growth chart.

The BMI charts have an upper limit of 26. They were constructed by the WHO using length for children under 2 years and height for children aged 2 years and older.[1]

Plotting and Interpreting BMI

Plotting BMI-for-age

For BMI to be a clinically useful tool, it must be plotted on a growth chart. Absolute BMI may only be used for individuals 18 years of age.[2] Computer software, cellphone applications, or growth charts can be used to plot BMI-for-age and determine the BMI-for-age z-score.

BMI-for-age is an important growth indicator for screening overweight and obesity. BMI does not distinguish adipose tissue from lean tissue.

Computer Software

The WHO provides two independent computer programs for anthropometric calculations, Anthro and AnthroPlus, which can be downloaded to a computer at the following links[1]:

- Anthro: 0 to 5 years old: http://www.who.int/childgrowth/software/en/
- AnthroPlus: >5 years old: http://www.who.int/growthref/tools/en/

The Anthro and AnthroPlus software consists of the following modules:

- Anthropometric calculator: This module facilitates deriving nutritional status results for an individual child or adolescent based on the WHO reference standards (*0-5 years or 5-19 years*) depending on his/her age for the indicators weight-for-age, length/height-for-age and BMI-for-age. This module does not save data to enable comparison of multiple data points.
- Individual assessment: This module enables the user to collect and save data for children who are repeatedly examined up to the age of 19 years.
- Nutritional survey: This module enables the user to collect and save data for groups of children.

Cellphone Applications

The AnthroCalc app calculates percentiles and z-scores for length/height-for-age, weight-for-age, and BMI-for-age using WHO growth charts.[8] However, unlike Anthro and AnthroPlus, the app does not save information for repeated assessments. The AnthroCalc app is available for download on Android and Apple phones through the following links:

- Apple: https://apps.apple.com/us/app/anthrocalc/id1521729239
- Android: https://play.google.com/store/apps/details?id=appinventor.ai_dlmetzger58.AnthroCalc&hl=en_US&gl=US

To determine BMI-for-age z-score using AnthroCalc:

- Select "Child Growth" on the home screen
- Select "WHO Growth Charts"
- Enter date of birth and date of measurement
- Select sex
- Enter measured length/height and weight
- Select calculate

WHO Growth Charts

The WHO growth charts for BMI-for-age may be found in Appendix B. The y-axis shows the child's BMI and x-axis shows the age in months and years.

The WHO recommends the following steps for plotting BMI-for-age:[1]

- Age should be plotted in completed weeks, months or years and months on a vertical line and not between the vertical lines.
- BMI should be plotted on a horizontal line (*e.g., 14, 14.2*), or in the space between lines (*e.g., 14.5*). If BMI was calculated using a calculator, record and plot to one decimal place.
- For multiple visits, connect adjacent points on a straight line to observe the trends in BMI.

Case Example: Plotting BMI-for-age

Source: WHO[1]

7-month-old female's BMI-for-age

BMI-for-age is plotted for two visits. The horizontal lines represent 0.2 BMI units. At the first visit, she is 7 months old and has a BMI of 17. Refer to the next case example to plot and calculate her age and BMI in the next two visits.

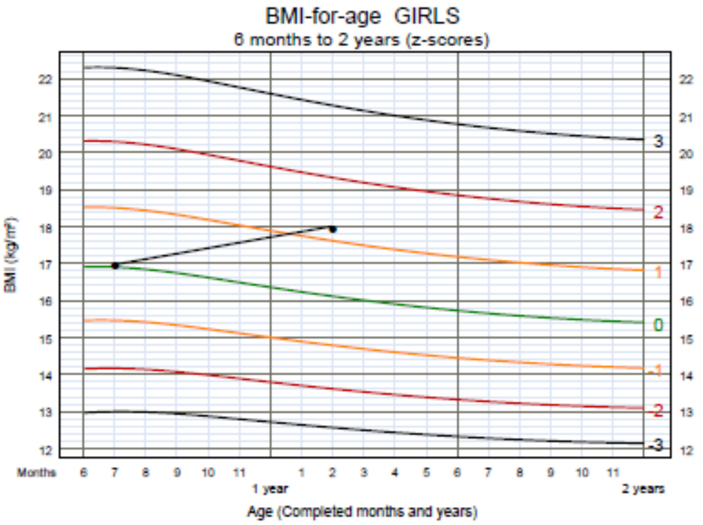

Case Example: Plotting BMI at Multiple Time Points

Source: WHO[1]

7-month-old female's BMI-for-age at multiple visits

How old is this patient at the second visit? Answer: 1 year and 2 months

What is her BMI at the second visit? Answer: BMI is 18 kg/m²

Plot the point for her next expected visit, when she is 1 year and 10 months old and has a BMI of 17.5. Connect the points as illustrated in the figure.

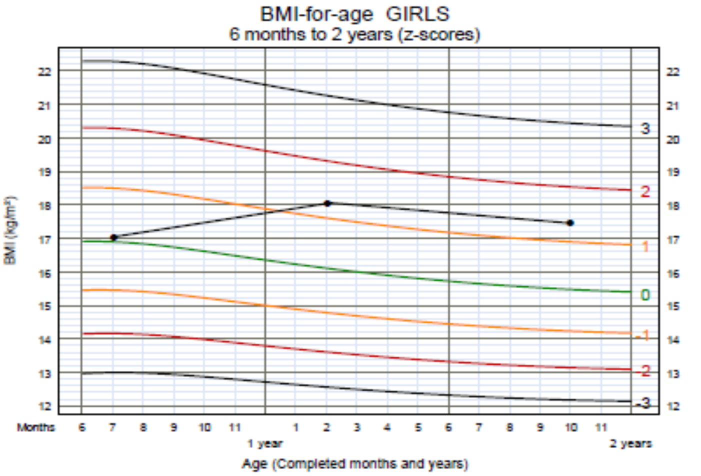

Interpret Plotted Points for Growth Indicators

Source: WHO[1]

Plot the BMI value on the growth curve to determine BMI percentile or z-score. The curved lines on the growth charts help interpret the plotted points that represent a child's growth status. The line labeled 0

on each chart represents the median, which is the average. The other curved lines are z-score lines, which indicate distance from the average. Z-scores are also called standard deviation scores.[1]

On the growth charts, z-score lines are positive (1, 2, 3) or negative (-1, -2, -3) numbers. If a point is far from the median in either direction (*for example, close to the 3 or -3 z-score line*), it may represent a growth problem. Other factors must also be considered such as the growth trend, the health condition of the child, and the height of the parents.[1]

Identify Growth Problems from Plotted Points

Source: WHO[1]

Table 1 provides classifications of nutritional status based on the z-score of the child as per WHO. Table 2 provides the classification of nutritional status based on z-score by the Centers for Disease Control and Prevention (CDC). It is important to note that the WHO and CDC have different cutoff points for classifying nutritional status.

The z-score is classified by being plotted above or below a particular z-score line. If the value falls exactly on the z-score line, it is considered in the less severe category. For example, if using the WHO reference, BMI-for-age on the -3 line is considered "wasted" as opposed to "severely wasted." Refer to the growth charts for BMI-for-age and height-for-age in Appendix B. The growth charts are constructed by the WHO and the values on the chart are read as follows:

- A point between the z-score lines -2 and -3 is "below -2."
- A point between the z-score lines 2 and 3 is "above 2."

Table 1. WHO nutritional classifications based on z-scores

Z-score	Nutritional Classification for Length/height-for-age	Nutritional Classification for BMI-for-age (under 5 years of age)	Nutritional Classification for BMI-for-age (5-19 years of age)
Above 3	Refer to Legend[1]	Obese	Severely Obese
Above 2		Overweight	Obese
Above 1		Possible risk of overweight[2]	Overweight
0 (median)			
Below -1			
Below -2	Stunted[3]	Wasted	Wasted
Below -3	Severely stunted[3]	Severely Wasted[4]	Severely Wasted[4]

[1]Very tall. Rarely a problem. If excessive, it may indicate an endocrine disorder such as a growth hormone-producing tumor. Refer to a physician. Refer to WHO growth charts in Appendix B to plot height-for-age.

[2]A plotted point above a z-score of 1 indicates possible risk. A trend towards the 2 z-score line may indicate risk of overweight/obesity.

[3]It is possible for a stunted or severely stunted child to become overweight. Refer to WHO growth charts in Appendix B to plot height-for-age.

[4]This indicates severe acute malnutrition, refer to a physician. Refer to the WHO Guidelines for its management.

Sources: World Health Organization. *Training Course on Child Growth Assessment*. Geneva, Switzerland: World Health Organization; 2008. https://www.who.int/publications/i/item/9789241595070; World Health Organization. *Obesity and overweight*. Geneva, Switzerland: World Health Organization; 2025. https://www.who.int/news-room/fact-sheets/detail/obesity-and-overweight

Table 2. CDC nutritional classifications based on BMI-for-age z-score

Z-score	Nutritional Classification
< -1.65	Underweight
-1.65 to < 1.04	Healthy Weight
1.04 to < 1.65	Overweight
≥ +1.65	Obesity

Abbreviations: CDC, Center for Disease Control and Prevention; BMI, body mass index.
Source: Barlow SE. Expert committee recommendations regarding the prevention, assessment, and treatment of child and adolescent overweight and obesity: summary report. Pediatrics. 2007;120 Suppl 4:S164-192.

Trends in BMI-for-age

BMI does not typically increase with age as with weight and height. An infant's BMI sharply increases with weight gain, relative to length, in the first six months. However, from age two to five years, BMI remains relatively stable.

The WHO growth charts present both percentiles and z-scores for age groups from birth to five years and five to 19 years (Refer to Appendix B).[1] Table 1 provides the nutritional classification based on BMI z-score.The WHO recommends observing a child's growth problems in reference to parental weight. The child's risk of being overweight increases by 40% if one parent is obese, and by 70% if both parents are obese.[1] It is important to recognize that overweight and obesity can co-exist with stunting.[1] In the setting of pediatric oncology, nutritional classification must also consider the diagnosis, cancer therapy and its associated side effects, and other existing nutritional conditions.[7]

Case Example: Observing Trends in BMI

Source: WHO[1]

4-year-old male's trends in BMI-for-age

The chart illustrates a trend towards overweight. If his growth line crosses the 2 z-score line, he will be considered overweight.

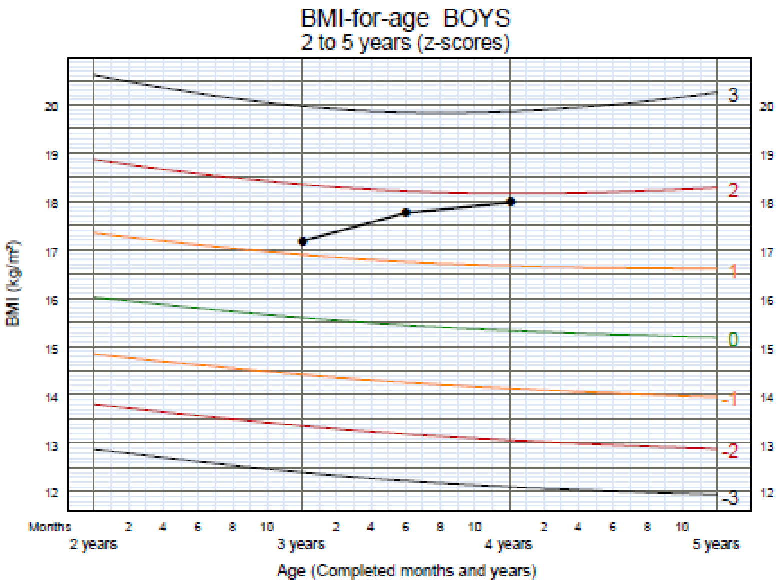

Mid-upper Arm Circumference (MUAC)

While it is common practice to use BMI-for-age to define acute malnutrition and height-for-age to define stunting, measures based on weight alone (*including BMI*) may be poor indicators of nutritional status in children with cancer[9] and it not useful in children with large solid tumor burden or with excessive edema. The use of MUAC is an ideal indicator to assess nutritional status in this setting.[9,10] UNICEF provides an easy-to-use, color-coded measuring tape, which allows for rapid assessment of nutritional status in children aged six months to five years.[3] Tapes may be obtained from the UNICEF supply office. Depending on the country location, a regional UNICEF office can provide more information on ways to access the tapes. Model number is S0145620 (Figure 5).

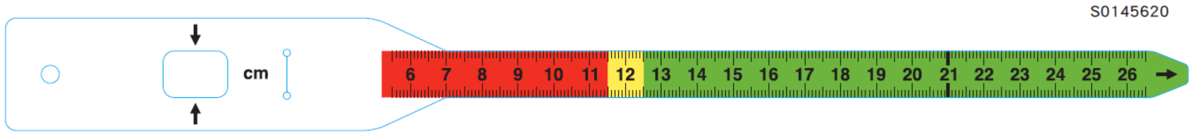

Figure 5. UNICEF MUAC tape model number S0145620 (2009)

MUAC Measurement

Measuring Tapes

The UNICEF MUAC tape (Figure 5) is used for children six months to five years. The colors, red *(<115 mm*), yellow *(≥115-<125 mm*), and green *(125 mm*) correspond to the nutritional classifications of

severe acute malnutrition, acute malnutrition, and normal, respectively. Flexible measuring tapes that can measure to the 0.1 cm may also be used.

Obtaining the Measurement

Steps to measure MUAC (Figure 6):[3]

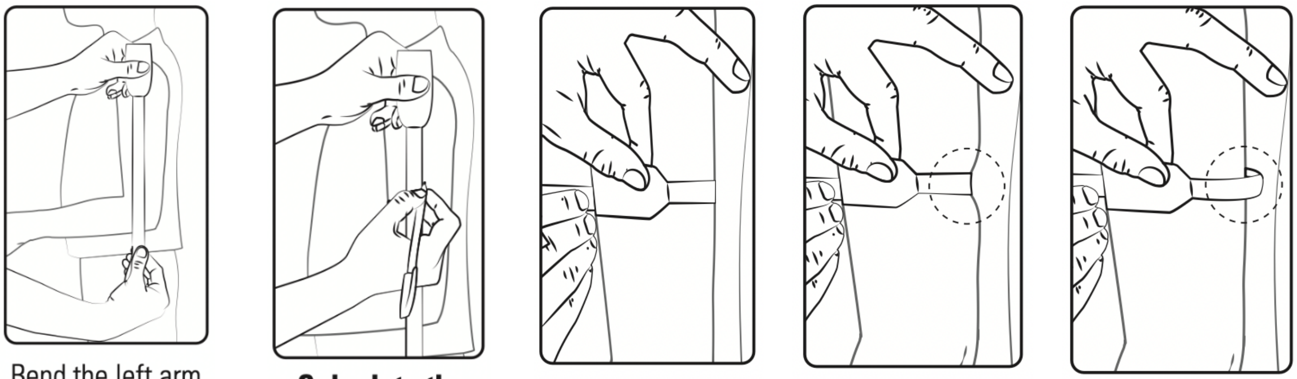

Figure 6. MUAC measurement (Source: UNICEF[3])

Classification of Malnutrition

Table 3 provides the measurements and their corresponding classification for acute malnutrition and severe acute malnutrition based on MUAC.[1,11]

Table 3. MUAC cut-off for malnutrition

Age	Severe wasting	Moderate wasting	No wasting
6-59 months	<11.5 cm (*z-scores of* -3)	≥11.5 - <12.5 cm *(z-scores of -2)*	≥12.5 cm
5-9 years	<13.0 cm	≥13.0 - <14.5 cm	≥14.5 cm
10-15 years	<16.0 cm	≥16.0 - <18.5 cm	≥18.5 cm

Abbreviations: cm, centimeter.

Sources: World Health Organization. *Training Course on Child Growth Assessment*. Geneva, Switzerland: World Health Organization; 2008. https://www.who.int/publications/i/item/9789241595070; Malawi Ministry of Health. *Guidelines for Community-Based Management of Acute Malnutrition*. Lilongwe, Malawi: Malawi Ministry of Health; 2016. https://www.fantaproject.org/sites/default/files/resources/Malawi-CMAM-Guidelines-Dec2016.pdf.

To determine MUAC-for-age z-score, Mramba growth charts can be used for children 5-19 years of age, using the growth charts in Appendix C or the following link: https://peditools.org/mrambamuac/.[12]

MUAC Indicators of Overweight and Obesity

With the increased prevalence of global obesity, classifications for overweight and obesity are being developed. For male and female children nine to 11 years of age, a MUAC measurement that is approximately 25 cm (*250 mm*) is classified as overweight/obese.[13] The number may vary by region, thus refer to country-specific cut-offs, if available. Region-specific values are presented in Table 4.

Table 4. Region-specific cut-offs for MUAC indicators of overnutrition

Region	Age	Overweight	
		Females	Males
South Africa	5-9 years	18.3/18.9 cm	18.4/18.6 cm
	10-14 years	22.5/22.8 cm	22.2/23.2 cm
		Overweight	Obesity
India	5-9 years	18.8 cm	19.4 cm
	10-14 years	23.0 cm	23.2 cm

Abbreviations: cm, centimeter.

Sources: Jaiswal M, Bansal R, Agarwal A. Role of mid-upper arm circumference for determining overweight and obesity in children and adolescents. *J Clin Diagn Res*. 2017;11(8):sc05-sc08.; Craig E, Bland R, Ndirangu J, Reilly JJ. Use of mid-upper arm circumference for determining overweight and overfatness in children and adolescents. *Arch Dis Child*. 2014;99(8):763-6.

Frequency of Anthropometric Monitoring

Anthropometric measurements, which include weight, height, and MUAC, should ideally be taken at every outpatient visit for all children. In the inpatient setting, these measurements should occur more frequently. Currently, there are no evidence-based guidelines regarding the frequency of anthropometric measurements for children and adolescents with cancer in the hospital setting. However, based on clinical experience, the following can be used as a guide for patients experiencing severe or moderate acute malnutrition:

- Daily weight measurements
- Weekly MUAC measurements
- Monthly height measurements

Daily weight measurements would not be warranted in a child with a large solid tumor. For patients who are not acutely malnourished, re-screening should be performed weekly to identify nutritional risk factors that would warrant a full nutrition assessment.

References

1. World Health Organization. *Training Course on Child Growth Assessment*. Geneva, Switzerland: World Health Organization; 2008. https://www.who.int/publications/i/item/9789241595070

2. Center for Disease Control and Prevention. *National Health and Nutritional Examination Survey (NHANES): Anthropometry Procedures Manual*. Center for Disease Control and Prevention; 2007. https://www.cdc.gov/nchs/data/nhanes/nhanes_07_08/manual_an.pdf

3. United Nation's Children's Fund. *MUAC Measuring Tapes*. United Nation's Children's Fund; 2020. https://www.unicef.org/supply/media/3941/file/child-design-tape-en.pdf

4. United States Agency for International Development, PATH. *Weight Scales: Guide to Selection*. Seattle, WA: PATH; 2016. https://media.path.org/documents/PATH_weight_scales.pdf

5. Osterkamp LK. Current perspective on assessment of human body proportions of relevance to amputees. *J Am Diet Assoc*. 1995;95(2):215-218.

6. Lemos P, Teles N, Guedes K, et al. *Avaliação Antropometria em Pacientes Oncológicos Pediátricos*. São Paulo, Brazil: Sociedade Brasileira de Oncologia Pediátrica (SOBOPE) - Comitê de Nutrição; 2015:1-14.

7. Gray DS, Crider JB, Kelley C, Dickinson LC. Accuracy of recumbent height measurement. *J Parenter Enteral Nutr*. 1985;9(6):712-715.

8. BC Children's Hospital. Tools & calculators: Anthropometric calculators. Accessed July 28, 2022. http://www.bcchildrens.ca/health-professionals/clinical-resources/endocrinology-diabetes/tools-calculators#Anthro--calculators

9. Ladas EJ, Arora B, Howard SC, Rogers PC, Mosby TT, Barr RD. A framework for adapted nutritional therapy for children with cancer in low- and middle-income countries: a report from the SIOP PODC Nutrition Working Group. *Pediatr Blood Cancer*. 2016;63(8):1339-1348.

10. Barr R, Atkinson S, Pencharz P, Arguelles GR. Nutrition and cancer in children. *Pediatr Blood Cancer*. 2008;50(2 Suppl):437.

11. Malawi Ministry of Health. *Guidelines for Community-Based Management of Acute Malnutrition*. Lilongwe, Malawi: Malawi Ministry of Health; 2016. https://www.fantaproject.org/sites/default/files/resources/Malawi-CMAM-Guidelines-Dec2016.pdf

12. Mramba L, Ngari M, Mwangome M, et al. A growth reference for mid upper arm circumference for age among school age children and adolescents, and validation for mortality: growth curve construction and longitudinal cohort study. *BMJ*. 2017;358:j3423.

13. Chaput JP, Katzmarzyk PT, Barnes JD, et al. Mid-upper arm circumference as a screening tool for identifying children with obesity: a 12-country study. *Pediatr Obes*. 2017;12(6):439-445.

14. World Health Organization. *Child Growth Standards*. Geneva, Switzerland: World Health Organization; 2006. https://www.who.int/tools/child-growth-standards/standards

15. World Health Organization. *Growth Reference Data for 5-19 Years*. Geneva, Switzerland: World Health Organization; 2007. https://www.who.int/tools/growth-reference-data-for-5to19-years

Additional Reading

Arora B, Ladas EJ. International nutrition capacity building: a global SIOP-PODC model from India. *Indian J Cancer*. 2015;52(2):163-6.

Barr R, Atkinson S, Pencharz P, Arguelles GR. Nutrition and cancer in children. *Pediatr Blood Cancer*. 2008;50(2 Suppl):437.

Fleming CA, Viani K, Murphy AJ, et al. The development, testing, and preliminary feasibility of an adaptable pediatric oncology nutrition algorithm for low-middle income countries. *Indian J Cancer*. 2015;52(2):225-8.
Iniesta RR, Paciarotti I, Brougham MF, McKenzie JM, Wilson DC. Effects of pediatric cancer and its treatment on nutritional status: a systematic review. *Nutr Rev*. 2015;73(5):276-95.
Ladas EJ. Nutrition therapy: support for integration into cancer care. *Pediatr Blood Cancer*. 2013;60(6):895-6.
Murphy AJ, Mosby TT, Rogers PC, Cohen J, Ladas EJ. An international survey of nutritional practices in low- and middle-income countries: a report from the International Society of Pediatric Oncology (SIOP) PODC Nutrition Working Group. *Eur J Clin Nutr.* 2014;68(12):1341-5.
Rogers PC. Importance of nutrition in pediatric oncology. *Indian J Cancer*. 2015;52(2):176-8.
Schoeman J. Nutritional assessment and intervention in a pediatric oncology unit. *Indian J Cancer*. 2015;52(2):186-90.
Viani K. Parenteral and enteral nutrition for pediatric oncology in low- and middle-income countries. *Indian J Cancer*. 2015;52(2):182-4.

Appendix A: BMI Tables

Table A-1. BMI for Corresponding Height (cm) and Weight (kg)

L or H (cm)	Body Mass Index (BMI)																			L or H (cm)
	8	9	10	11	12	13	14	15	16	17	18	19	20	21	22	23	24	25	26	
42	1.4	1.6	1.8	1.9	2.1	2.3	2.5	2.6	2.8	3.0	3.2	3.4	3.5	3.7	3.9	4.1	4.2	4.4	4.6	42
43	1.5	1.7	1.8	2.0	2.2	2.4	2.6	2.8	3.0	3.1	3.3	3.5	3.7	3.9	4.1	4.3	4.4	4.6	4.8	43
44	1.5	1.7	1.9	2.1	2.3	2.5	2.7	2.9	3.1	3.3	3.5	3.7	3.9	4.1	4.3	4.5	4.6	4.8	5.0	44
45	1.6	1.8	2.0	2.2	2.4	2.6	2.8	3.0	3.2	3.4	3.6	3.8	4.1	4.3	4.5	4.7	4.9	5.1	5.3	45
46	1.7	1.9	2.1	2.3	2.5	2.8	3.0	3.2	3.4	3.6	3.8	4.0	4.2	4.4	4.7	4.9	5.1	5.3	5.5	46
47	1.8	2.0	2.2	2.4	2.7	2.9	3.1	3.3	3.5	3.8	4.0	4.2	4.4	4.6	4.9	5.1	5.3	5.5	5.7	47
48	1.8	2.1	2.3	2.5	2.8	3.0	3.2	3.5	3.7	3.9	4.1	4.4	4.6	4.8	5.1	5.3	5.5	5.8	6.0	48
49	1.9	2.2	2.4	2.6	2.9	3.1	3.4	3.6	3.8	4.1	4.3	4.6	4.8	5.0	5.3	5.5	5.8	6.0	6.2	49
50	2.0	2.3	2.5	2.8	3.0	3.3	3.5	3.8	4.0	4.3	4.5	4.8	5.0	5.3	5.5	5.8	6.0	6.3	6.5	50
51	2.1	2.3	2.6	2.9	3.1	3.4	3.6	3.9	4.2	4.4	4.7	4.9	5.2	5.5	5.7	6.0	6.2	6.5	6.8	51
52	2.2	2.4	2.7	3.0	3.2	3.5	3.8	4.1	4.3	4.6	4.9	5.1	5.4	5.7	5.9	6.2	6.5	6.8	7.0	52
53	2.2	2.5	2.8	3.1	3.4	3.7	3.9	4.2	4.5	4.8	5.1	5.3	5.6	5.9	6.2	6.5	6.7	7.0	7.3	53
54	2.3	2.6	2.9	3.2	3.5	3.8	4.1	4.4	4.7	5.0	5.2	5.5	5.8	6.1	6.4	6.7	7.0	7.3	7.6	54
55	2.4	2.7	3.0	3.3	3.6	3.9	4.2	4.5	4.8	5.1	5.4	5.7	6.1	6.4	6.7	7.0	7.3	7.6	7.9	55
56	2.5	2.8	3.1	3.4	3.8	4.1	4.4	4.7	5.0	5.3	5.6	6.0	6.3	6.6	6.9	7.2	7.5	7.8	8.2	56
57	2.6	2.9	3.2	3.6	3.9	4.2	4.5	4.9	5.2	5.5	5.8	6.2	6.5	6.8	7.1	7.5	7.8	8.1	8.4	57
58	2.7	3.0	3.4	3.7	4.0	4.4	4.7	5.0	5.4	5.7	6.1	6.4	6.7	7.1	7.4	7.7	8.1	8.4	8.7	58
59	2.8	3.1	3.5	3.8	4.2	4.5	4.9	5.2	5.6	5.9	6.3	6.6	7.0	7.3	7.7	8.0	8.4	8.7	9.1	59
60	2.9	3.2	3.6	4.0	4.3	4.7	5.0	5.4	5.8	6.1	6.5	6.8	7.2	7.6	7.9	8.3	8.6	9.0	9.4	60
61	3.0	3.3	3.7	4.1	4.5	4.8	5.2	5.6	6.0	6.3	6.7	7.1	7.4	7.8	8.2	8.6	8.9	9.3	9.7	61
62	3.1	3.5	3.8	4.2	4.6	5.0	5.4	5.8	6.2	6.5	6.9	7.3	7.7	8.1	8.5	8.8	9.2	9.6	10.0	62
63	3.2	3.6	4.0	4.4	4.8	5.2	5.6	6.0	6.4	6.7	7.1	7.5	7.9	8.3	8.7	9.1	9.5	9.9	10.3	63
64	3.3	3.7	4.1	4.5	4.9	5.3	5.7	6.1	6.6	7.0	7.4	7.8	8.2	8.6	9.0	9.4	9.8	10.2	10.6	64
65	3.4	3.8	4.2	4.6	5.1	5.5	5.9	6.3	6.8	7.2	7.6	8.0	8.5	8.9	9.3	9.7	10.1	10.6	11.0	65
66	3.5	3.9	4.4	4.8	5.2	5.7	6.1	6.5	7.0	7.4	7.8	8.3	8.7	9.1	9.6	10.0	10.5	10.9	11.3	66
67	3.6	4.0	4.5	4.9	5.4	5.8	6.3	6.7	7.2	7.6	8.1	8.5	9.0	9.4	9.9	10.3	10.8	11.2	11.7	67
68	3.7	4.2	4.6	5.1	5.5	6.0	6.5	6.9	7.4	7.9	8.3	8.8	9.2	9.7	10.2	10.6	11.1	11.6	12.0	68
69	3.8	4.3	4.8	5.2	5.7	6.2	6.7	7.1	7.6	8.1	8.6	9.0	9.5	10.0	10.5	11.0	11.4	11.9	12.4	69
70	3.9	4.4	4.9	5.4	5.9	6.4	6.9	7.4	7.8	8.3	8.8	9.3	9.8	10.3	10.8	11.3	11.8	12.3	12.7	70
71	4.0	4.5	5.0	5.5	6.0	6.6	7.1	7.6	8.1	8.6	9.1	9.6	10.1	10.6	11.1	11.6	12.1	12.6	13.1	71
72	4.1	4.7	5.2	5.7	6.2	6.7	7.3	7.8	8.3	8.8	9.3	9.8	10.4	10.9	11.4	11.9	12.4	13.0	13.5	72
73	4.3	4.8	5.3	5.9	6.4	6.9	7.5	8.0	8.5	9.1	9.6	10.1	10.7	11.2	11.7	12.3	12.8	13.3	13.9	73
74	4.4	4.9	5.5	6.0	6.6	7.1	7.7	8.2	8.8	9.3	9.9	10.4	11.0	11.5	12.0	12.6	13.1	13.7	14.2	74
75	4.5	5.1	5.6	6.2	6.8	7.3	7.9	8.4	9.0	9.6	10.1	10.7	11.3	11.8	12.4	12.9	13.5	14.1	14.6	75
76	4.6	5.2	5.8	6.4	6.9	7.5	8.1	8.7	9.2	9.8	10.4	11.0	11.6	12.1	12.7	13.3	13.9	14.4	15.0	76
77	4.7	5.3	5.9	6.5	7.1	7.7	8.3	8.9	9.5	10.1	10.7	11.3	11.9	12.5	13.0	13.6	14.2	14.8	15.4	77
78	4.9	5.5	6.1	6.7	7.3	7.9	8.5	9.1	9.7	10.3	11.0	11.6	12.2	12.8	13.4	14.0	14.6	15.2	15.8	78
79	5.0	5.6	6.2	6.9	7.5	8.1	8.7	9.4	10.0	10.6	11.2	11.9	12.5	13.1	13.7	14.4	15.0	15.6	16.2	79
80	5.1	5.8	6.4	7.0	7.7	8.3	9.0	9.6	10.2	10.9	11.5	12.2	12.8	13.4	14.1	14.7	15.4	16.0	16.6	80
81	5.2	5.9	6.6	7.2	7.9	8.5	9.2	9.8	10.5	11.2	11.8	12.5	13.1	13.8	14.4	15.1	15.7	16.4	17.1	81
82	5.4	6.1	6.7	7.4	8.1	8.7	9.4	10.1	10.8	11.4	12.1	12.8	13.4	14.1	14.8	15.5	16.1	16.8	17.5	82
83	5.5	6.2	6.9	7.6	8.3	9.0	9.6	10.3	11.0	11.7	12.4	13.1	13.8	14.5	15.2	15.8	16.5	17.2	17.9	83
	8	9	10	11	12	13	14	15	16	17	18	19	20	21	22	23	24	25	26	

Source: WHO[1]

Table A-2. BMI for Corresponding Height (cm) and Weight (kg)

L or H (cm)	Body Mass Index (BMI)																			L or H (cm)
	8	9	10	11	12	13	14	15	16	17	18	19	20	21	22	23	24	25	26	
84	5.6	6.4	7.1	7.8	8.5	9.2	9.9	10.6	11.3	12.0	12.7	13.4	14.1	14.8	15.5	16.2	16.9	17.6	18.3	84
85	5.8	6.5	7.2	7.9	8.7	9.4	10.1	10.8	11.6	12.3	13.0	13.7	14.5	15.2	15.9	16.6	17.3	18.1	18.8	85
86	5.9	6.7	7.4	8.1	8.9	9.6	10.4	11.1	11.8	12.6	13.3	14.1	14.8	15.5	16.3	17.0	17.8	18.5	19.2	86
87	6.1	6.8	7.6	8.3	9.1	9.8	10.6	11.4	12.1	12.9	13.6	14.4	15.1	15.9	16.7	17.4	18.2	18.9	19.7	87
88	6.2	7.0	7.7	8.5	9.3	10.1	10.8	11.6	12.4	13.2	13.9	14.7	15.5	16.3	17.0	17.8	18.6	19.4	20.1	88
89	6.3	7.1	7.9	8.7	9.5	10.3	11.1	11.9	12.7	13.5	14.3	15.0	15.8	16.6	17.4	18.2	19.0	19.8	20.6	89
90	6.5	7.3	8.1	8.9	9.7	10.5	11.3	12.2	13.0	13.8	14.6	15.4	16.2	17.0	17.8	18.6	19.4	20.3	21.1	90
91	6.6	7.5	8.3	9.1	9.9	10.8	11.6	12.4	13.2	14.1	14.9	15.7	16.6	17.4	18.2	19.0	19.9	20.7	21.5	91
92	6.8	7.6	8.5	9.3	10.2	11.0	11.8	12.7	13.5	14.4	15.2	16.1	16.9	17.8	18.6	19.5	20.3	21.2	22.0	92
93	6.9	7.8	8.6	9.5	10.4	11.2	12.1	13.0	13.8	14.7	15.6	16.4	17.3	18.2	19.0	19.9	20.8	21.6	22.5	93
94	7.1	8.0	8.8	9.7	10.6	11.5	12.4	13.3	14.1	15.0	15.9	16.8	17.7	18.6	19.4	20.3	21.2	22.1	23.0	94
95	7.2	8.1	9.0	9.9	10.8	11.7	12.6	13.5	14.4	15.3	16.2	17.1	18.1	19.0	19.9	20.8	21.7	22.6	23.5	95
96	7.4	8.3	9.2	10.1	11.1	12.0	12.9	13.8	14.7	15.7	16.6	17.5	18.4	19.4	20.3	21.2	22.1	23.0	24.0	96
97	7.5	8.5	9.4	10.3	11.3	12.2	13.2	14.1	15.1	16.0	16.9	17.9	18.8	19.8	20.7	21.6	22.6	23.5	24.5	97
98	7.7	8.6	9.6	10.6	11.5	12.5	13.4	14.4	15.4	16.3	17.3	18.2	19.2	20.2	21.1	22.1	23.0	24.0	25.0	98
99	7.8	8.8	9.8	10.8	11.8	12.7	13.7	14.7	15.7	16.7	17.6	18.6	19.6	20.6	21.6	22.5	23.5	24.5	25.5	99
100	8.0	9.0	10.0	11.0	12.0	13.0	14.0	15.0	16.0	17.0	18.0	19.0	20.0	21.0	22.0	23.0	24.0	25.0	26.0	100
101	8.2	9.2	10.2	11.2	12.2	13.3	14.3	15.3	16.3	17.3	18.4	19.4	20.4	21.4	22.4	23.5	24.5	25.5	26.5	101
102	8.3	9.4	10.4	11.4	12.5	13.5	14.6	15.6	16.6	17.7	18.7	19.8	20.8	21.8	22.9	23.9	25.0	26.0	27.1	102
103	8.5	9.5	10.6	11.7	12.7	13.8	14.9	15.9	17.0	18.0	19.1	20.2	21.2	22.3	23.3	24.4	25.5	26.5	27.6	103
104	8.7	9.7	10.8	11.9	13.0	14.1	15.1	16.2	17.3	18.4	19.5	20.6	21.6	22.7	23.8	24.9	26.0	27.0	28.1	104
105	8.8	9.9	11.0	12.1	13.2	14.3	15.4	16.5	17.6	18.7	19.8	20.9	22.1	23.2	24.3	25.4	26.5	27.6	28.7	105
106	9.0	10.1	11.2	12.4	13.5	14.6	15.7	16.9	18.0	19.1	20.2	21.3	22.5	23.6	24.7	25.8	27.0	28.1	29.2	106
107	9.2	10.3	11.4	12.6	13.7	14.9	16.0	17.2	18.3	19.5	20.6	21.8	22.9	24.0	25.2	26.3	27.5	28.6	29.8	107
108	9.3	10.5	11.7	12.8	14.0	15.2	16.3	17.5	18.7	19.8	21.0	22.2	23.3	24.5	25.7	26.8	28.0	29.2	30.3	108
109	9.5	10.7	11.9	13.1	14.3	15.4	16.6	17.8	19.0	20.2	21.4	22.6	23.8	25.0	26.1	27.3	28.5	29.7	30.9	109
110	9.7	10.9	12.1	13.3	14.5	15.7	16.9	18.2	19.4	20.6	21.8	23.0	24.2	25.4	26.6	27.8	29.0	30.3	31.5	110
111	9.9	11.1	12.3	13.6	14.8	16.0	17.2	18.5	19.7	20.9	22.2	23.4	24.6	25.9	27.1	28.3	29.6	30.8	32.0	111
112	10.0	11.3	12.5	13.8	15.1	16.3	17.6	18.8	20.1	21.3	22.6	23.8	25.1	26.3	27.6	28.9	30.1	31.4	32.6	112
113	10.2	11.5	12.8	14.0	15.3	16.6	17.9	19.2	20.4	21.7	23.0	24.3	25.5	26.8	28.1	29.4	30.6	31.9	33.2	113
114	10.4	11.7	13.0	14.3	15.6	16.9	18.2	19.5	20.8	22.1	23.4	24.7	26.0	27.3	28.6	29.9	31.2	32.5	33.8	114
115	10.6	11.9	13.2	14.5	15.9	17.2	18.5	19.8	21.2	22.5	23.8	25.1	26.5	27.8	29.1	30.4	31.7	33.1	34.4	115
116	10.8	12.1	13.5	14.8	16.1	17.5	18.8	20.2	21.5	22.9	24.2	25.6	26.9	28.3	29.6	30.9	32.3	33.6	35.0	116
117	11.0	12.3	13.7	15.1	16.4	17.8	19.2	20.5	21.9	23.3	24.6	26.0	27.4	28.7	30.1	31.5	32.9	34.2	35.6	117
118	11.1	12.5	13.9	15.3	16.7	18.1	19.5	20.9	22.3	23.7	25.1	26.5	27.8	29.2	30.6	32.0	33.4	34.8	36.2	118
119	11.3	12.7	14.2	15.6	17.0	18.4	19.8	21.2	22.7	24.1	25.5	26.9	28.3	29.7	31.2	32.6	34.0	35.4	36.8	119
120	11.5	13.0	14.4	15.8	17.3	18.7	20.2	21.6	23.0	24.5	25.9	27.4	28.8	30.2	31.7	33.1	34.6	36.0	37.4	120
121	11.7	13.2	14.6	16.1	17.6	19.0	20.5	22.0	23.4	24.9	26.4	27.8	29.3	30.7	32.2	33.7	35.1	36.6	38.1	121
122	11.9	13.4	14.9	16.4	17.9	19.3	20.8	22.3	23.8	25.3	26.8	28.3	29.8	31.3	32.7	34.2	35.7	37.2	38.7	122
123	12.1	13.6	15.1	16.6	18.2	19.7	21.2	22.7	24.2	25.7	27.2	28.7	30.3	31.8	33.3	34.8	36.3	37.8	39.3	123
124	12.3	13.8	15.4	16.9	18.5	20.0	21.5	23.1	24.6	26.1	27.7	29.2	30.8	32.3	33.8	35.4	36.9	38.4	40.0	124
125	12.5	14.1	15.6	17.2	18.8	20.3	21.9	23.4	25.0	26.6	28.1	29.7	31.3	32.8	34.4	35.9	37.5	39.1	40.6	125
	8	9	10	11	12	13	14	15	16	17	18	19	20	21	22	23	24	25	26	

Source: WHO[1]

Appendix B: Height-for-age and BMI-for-age Growth Charts

Figure B-1: Length/height-for-age Percentiles for Girls - Birth to 5 Years

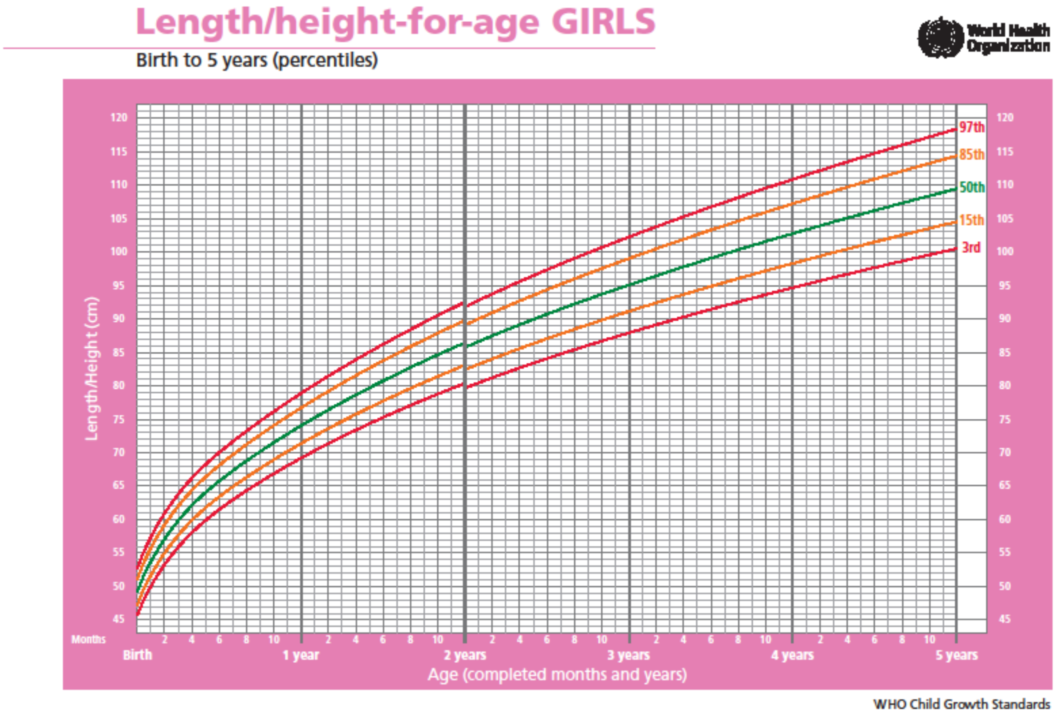

Source: WHO[14]

Figure B-2: Length/height-for-age Z-scores for Girls - Birth to 5 Years

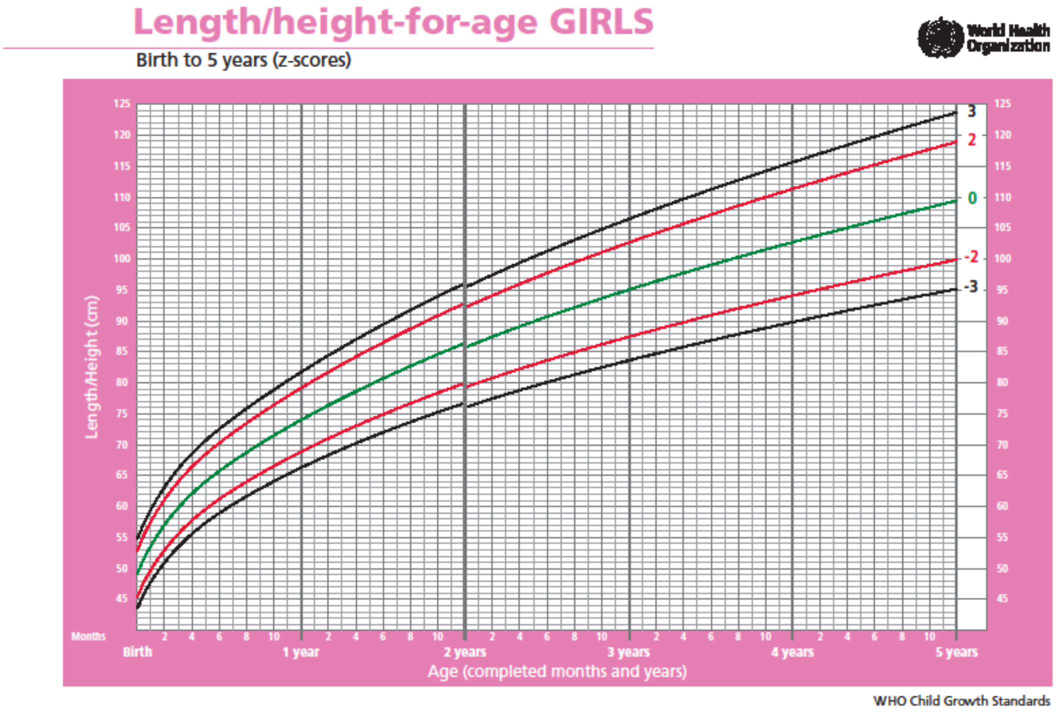

Source: WHO[14]

Figure B-3: Length/height-for-age Percentiles for Boys - Birth to 5 Years

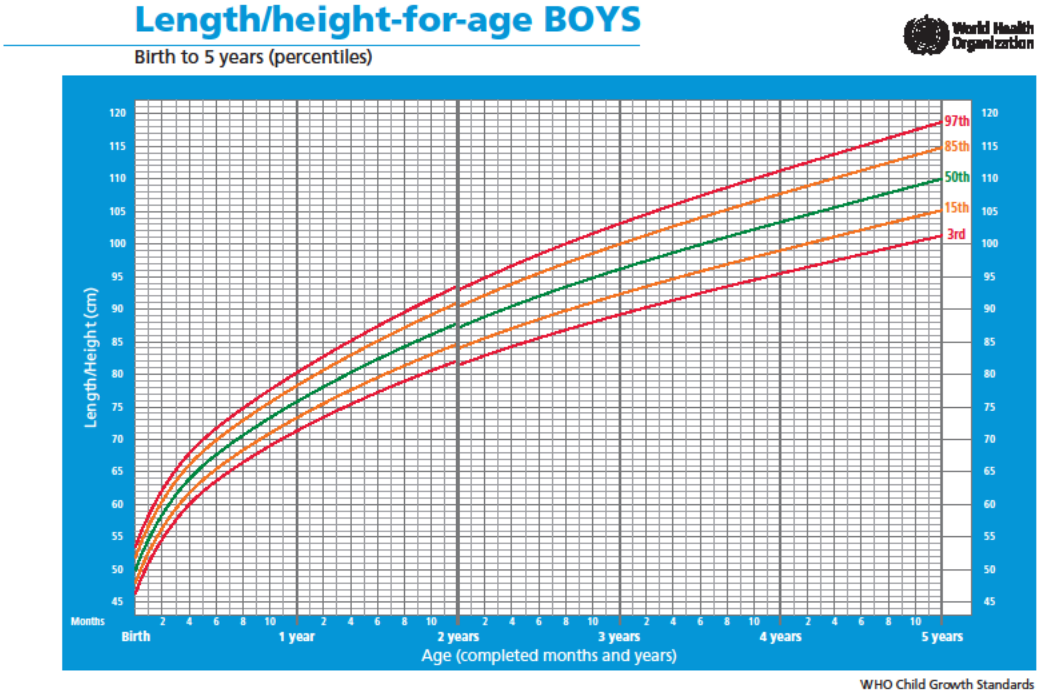

Source: WHO[14]

Figure B-4: Length/height-for-age Z-scores for Boys - Birth to 5 Years

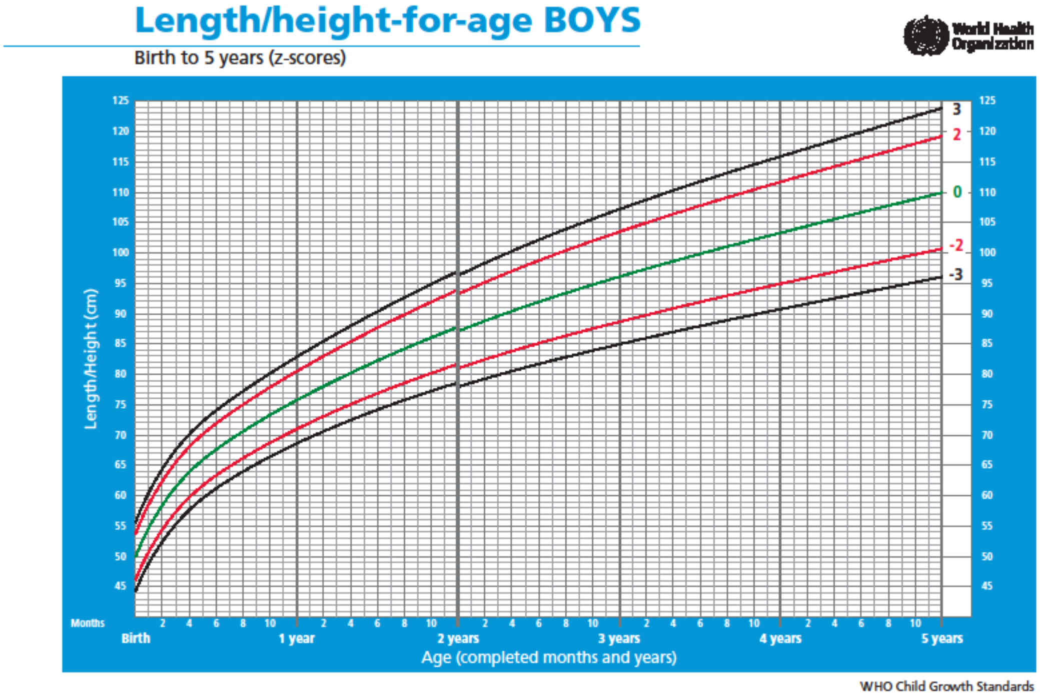

Source: WHO[14]

IIPAN

COLUMBIA
COLUMBIA UNIVERSITY
IRVING MEDICAL CENTER

Figure B-5: Height-for-age Percentiles for Girls - 5 to 19 Years

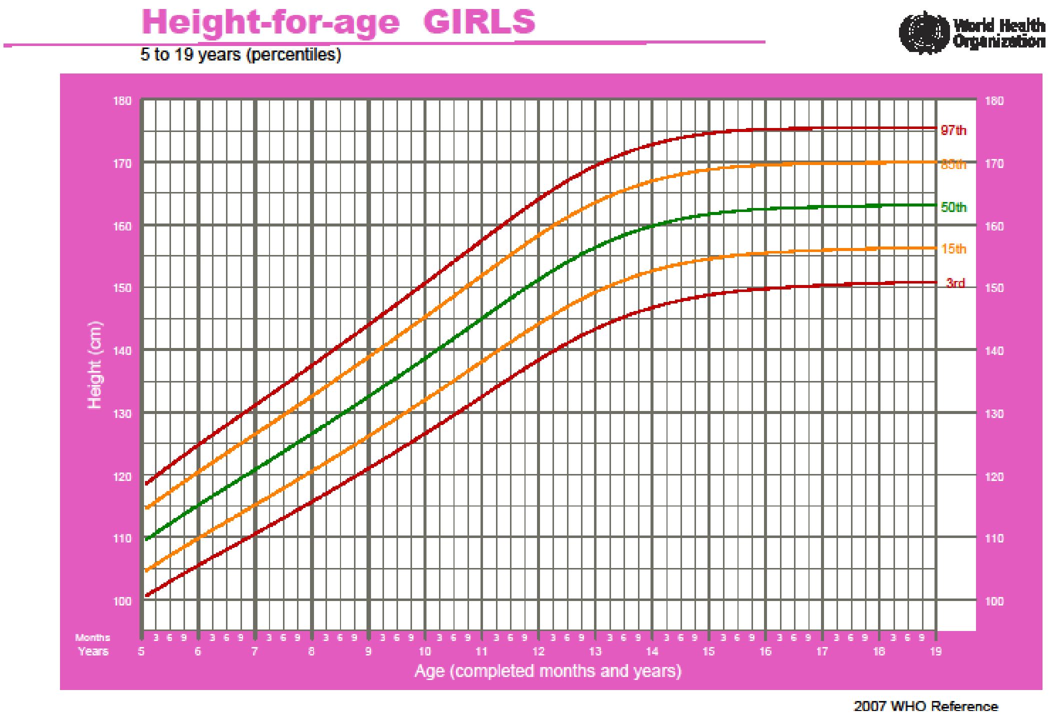

Source: WHO[14]

Figure B-6: Height-for-age Z-scores for Girls - 5 to 19 Years

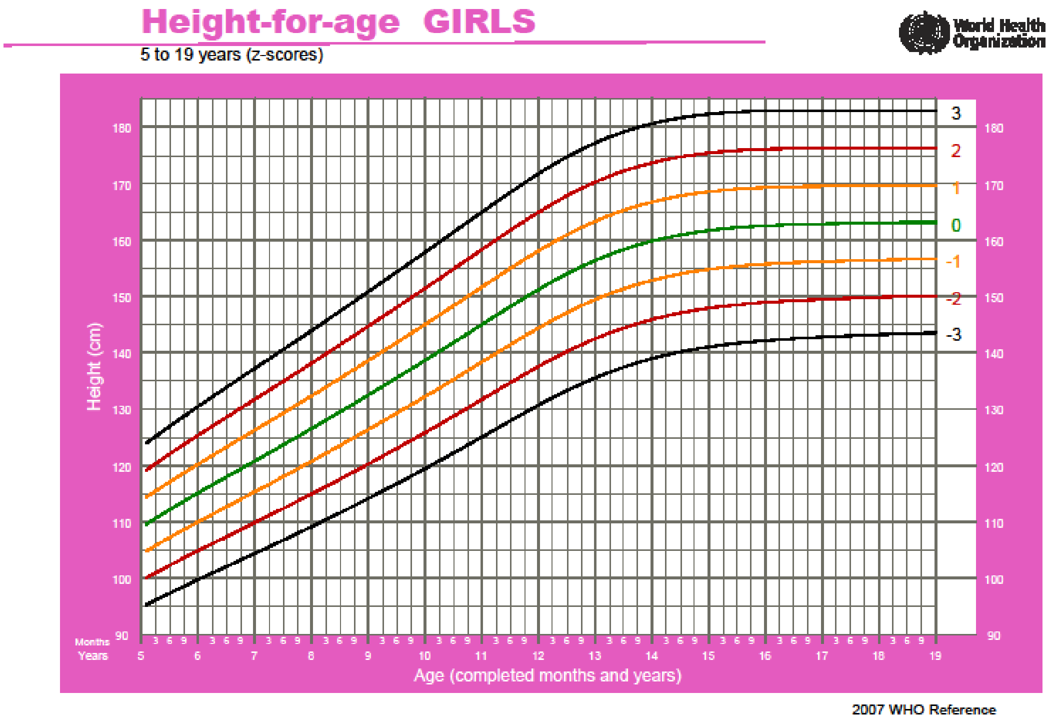

Source: WHO[14]

Figure B-7: Height-for-age Percentiles for Boys - 5 to 19 Years

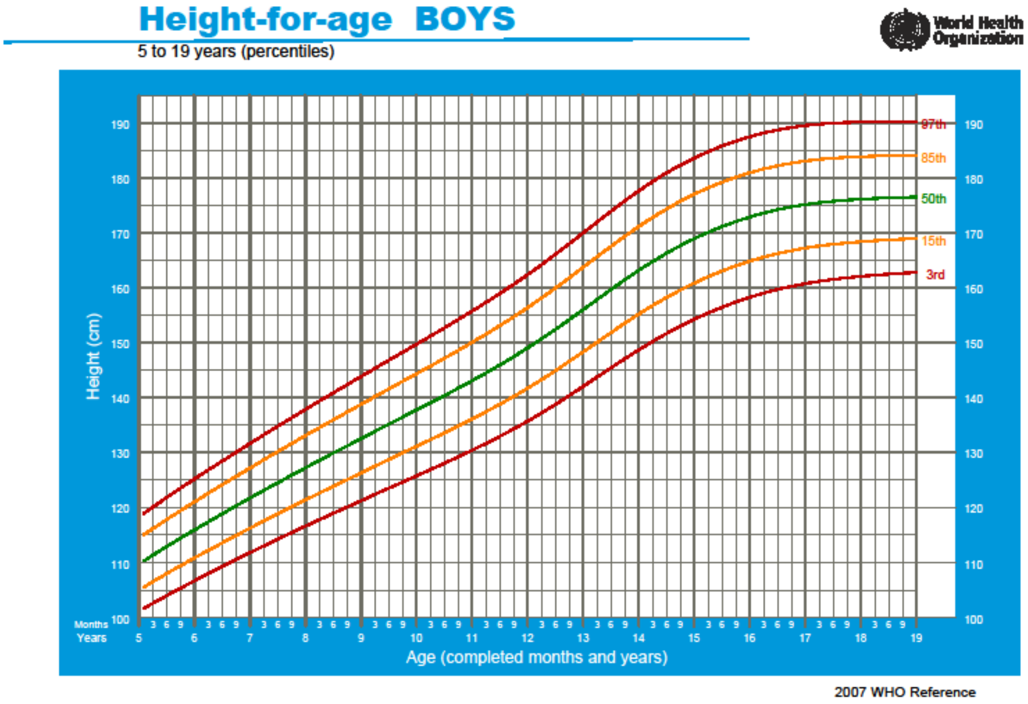

Source: WHO[14]

Figure B-8: Height-for-age Z-scores for Boys - 5 to 19 Years

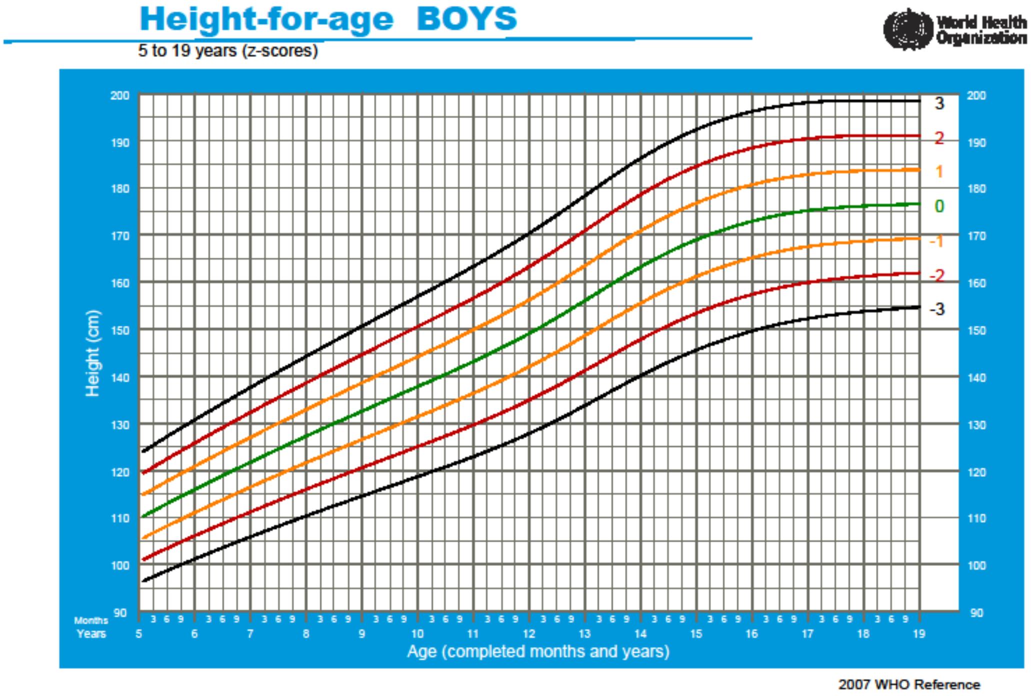

Source: WHO[14]

Figure B-9. BMI-for-age Percentiles for Girls - Birth to 5 Years

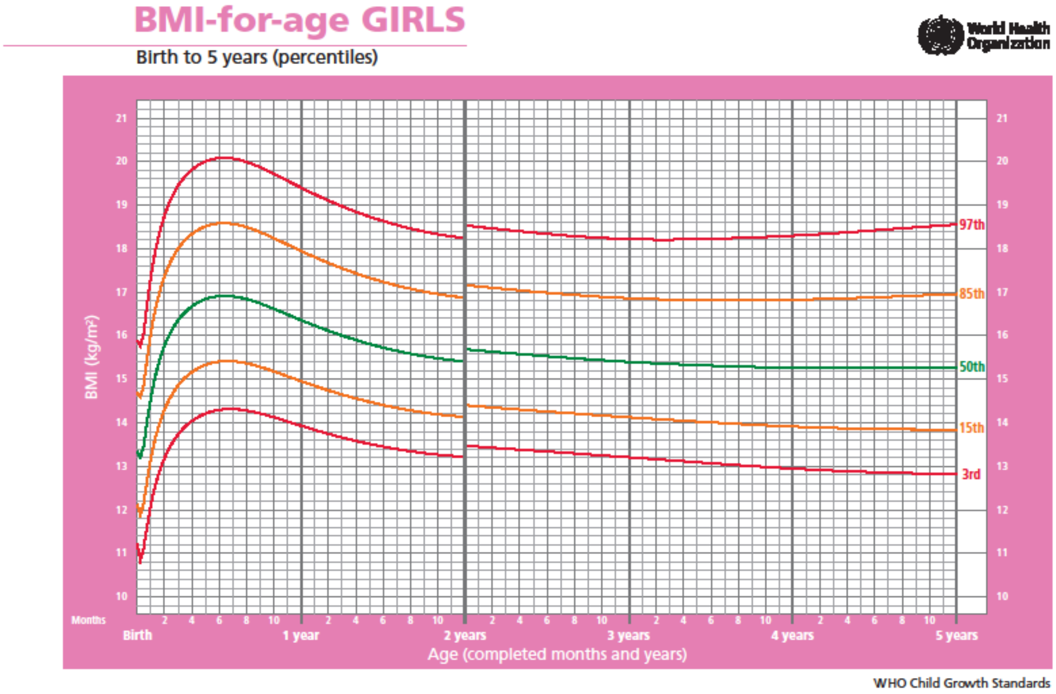

Source: WHO[14]

Figure B-10. BMI-for-age Z-scores for Girls - Birth to 5 Years

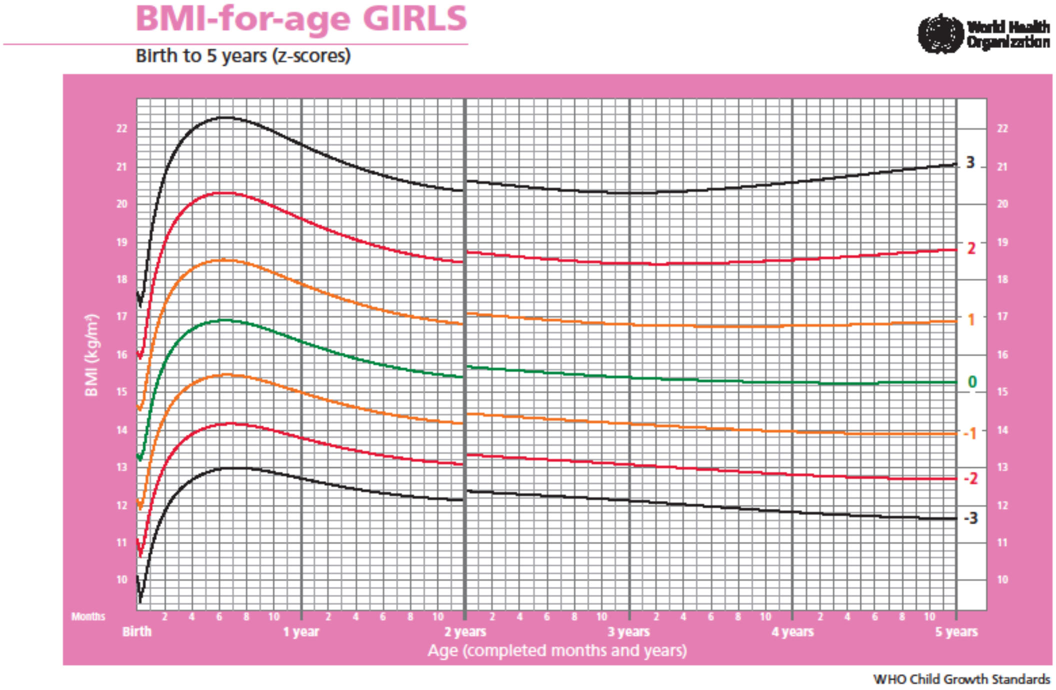

Source: WHO[14]

Figure B-11. BMI-for-age Percentiles for Boys - Birth to 5 Years

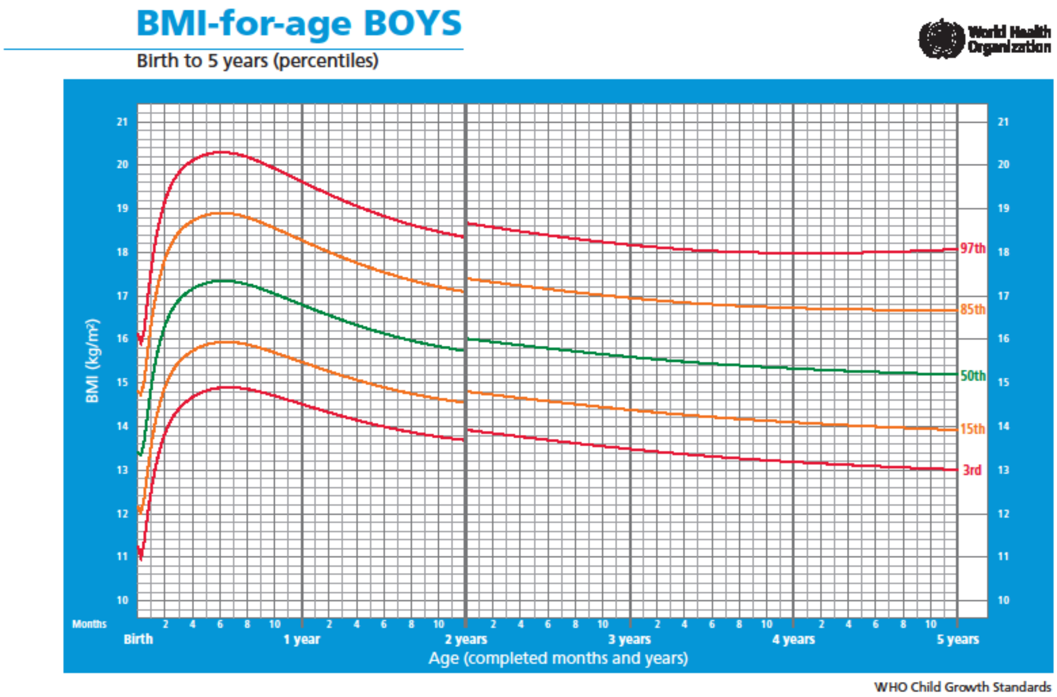

Source: WHO[14]

Figure B-12. BMI-for-age Z-scores for Boys - Birth to 5 Years

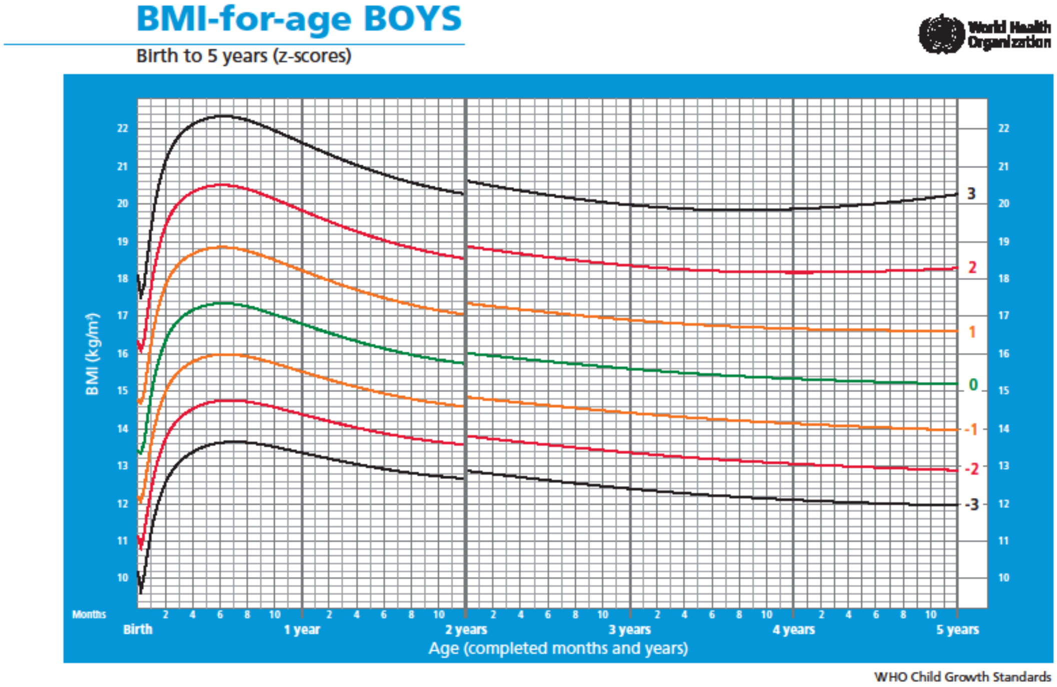

Source: WHO[14]

Figure B-13. BMI-for-age Percentiles for Girls - 5 to 19 Years

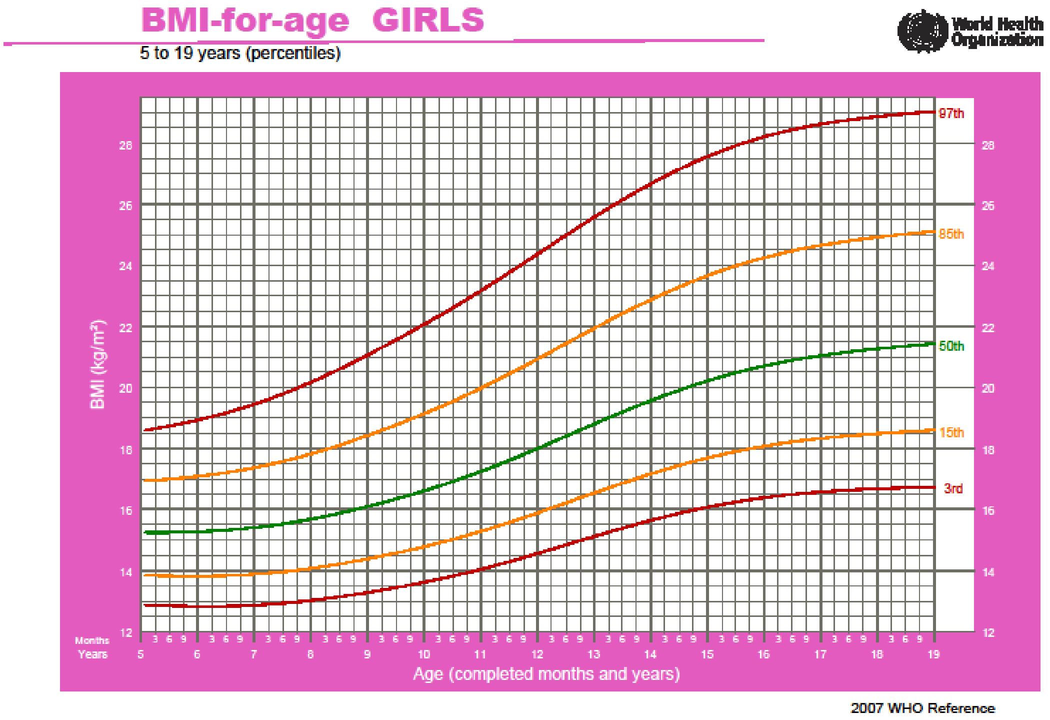

Source: WHO[15]

Figure B-14. BMI-for-age Z-scores for Girls - 5 to 19 Years

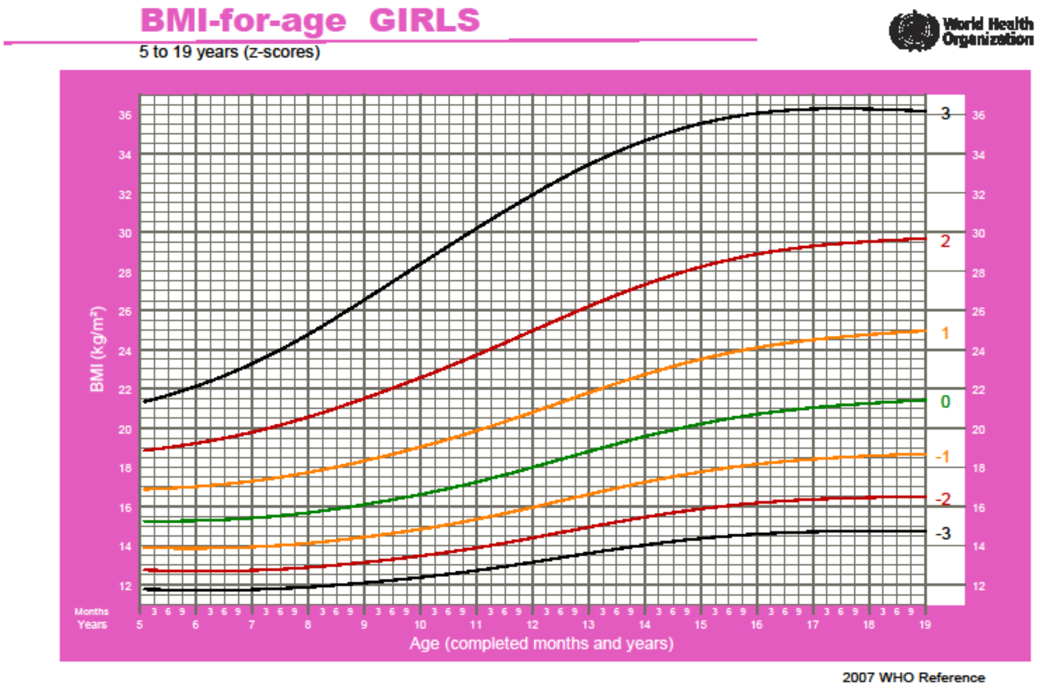

Source: WHO[15]

Figure B-15. BMI-for-age Percentiles for Boys - 5 to 19 Years

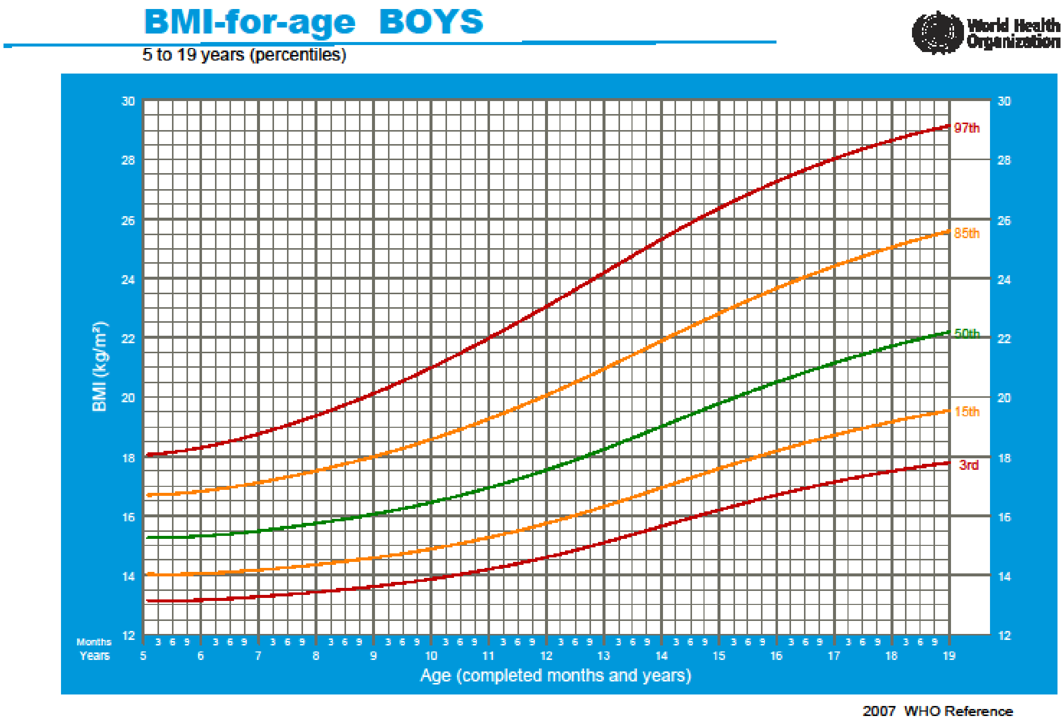

Source: WHO[15]

Figure B-16. BMI-for-age Z-scores for Boys - 5 to 19 Years

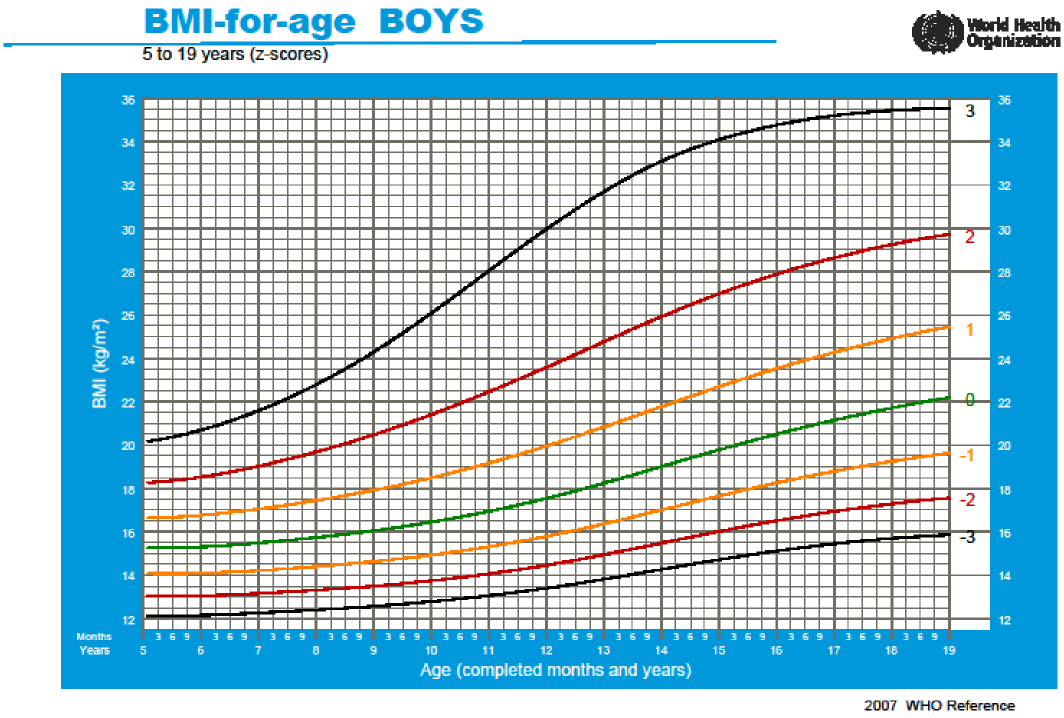

Source: WHO[15]

Appendix C: MUAC-for-age Growth Charts

Figure C-1. MUAC-for-age Percentiles for Girls - 3 Months to 5 Years

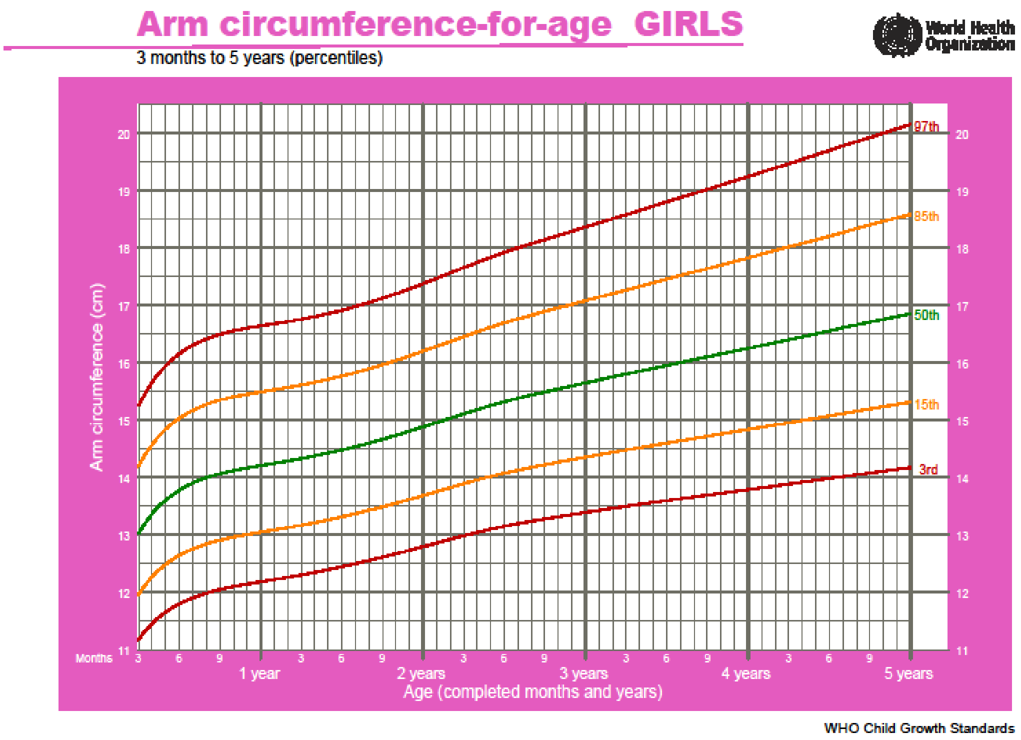

Source: WHO[14]

Figure C-2. MUAC-for-age Z-scores for Girls - 3 Months to 5 Years

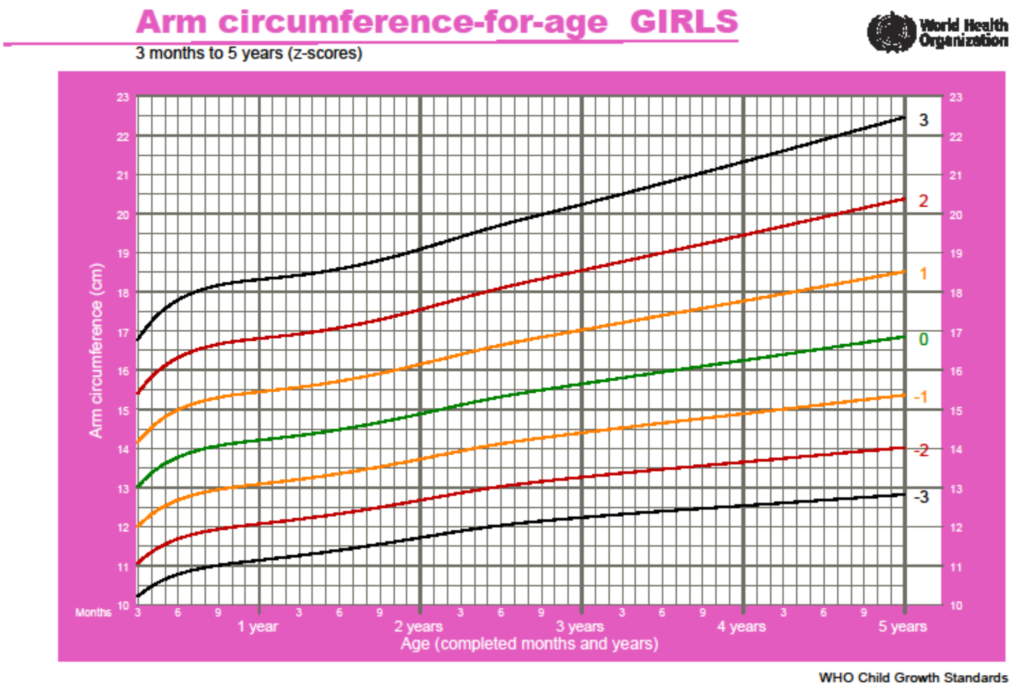

Source: WHO[14]

Figure C-3. MUAC-for-age Percentiles for Boys - 3 Months to 5 Years

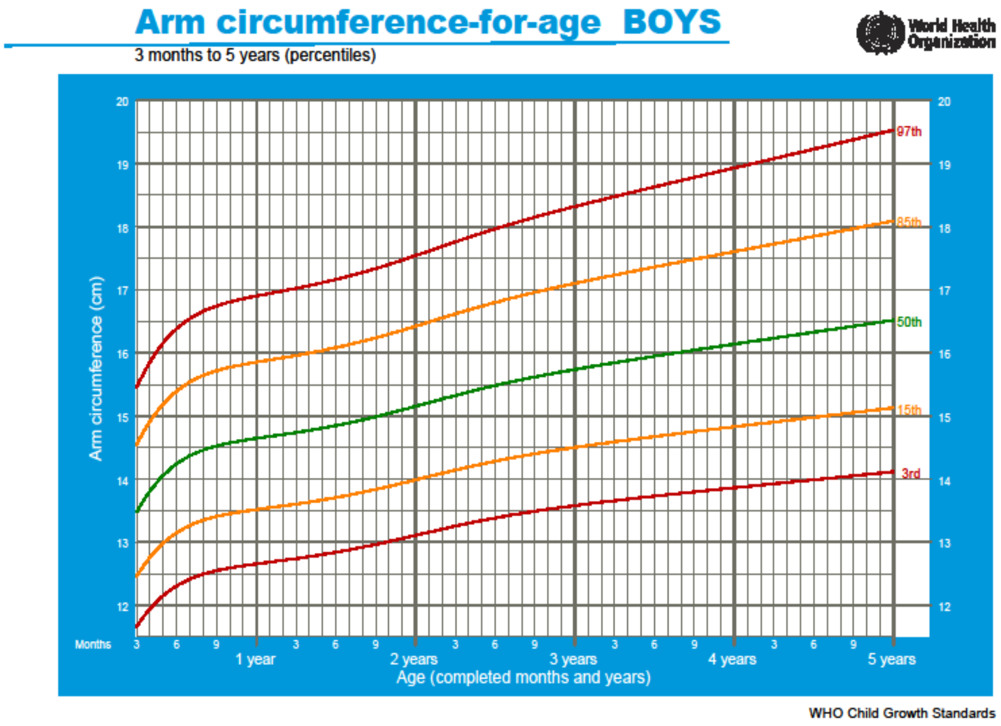

Source: WHO[14]

Figure C-4. MUAC-for-age Z-scores for Boys - 3 Months to 5 Years

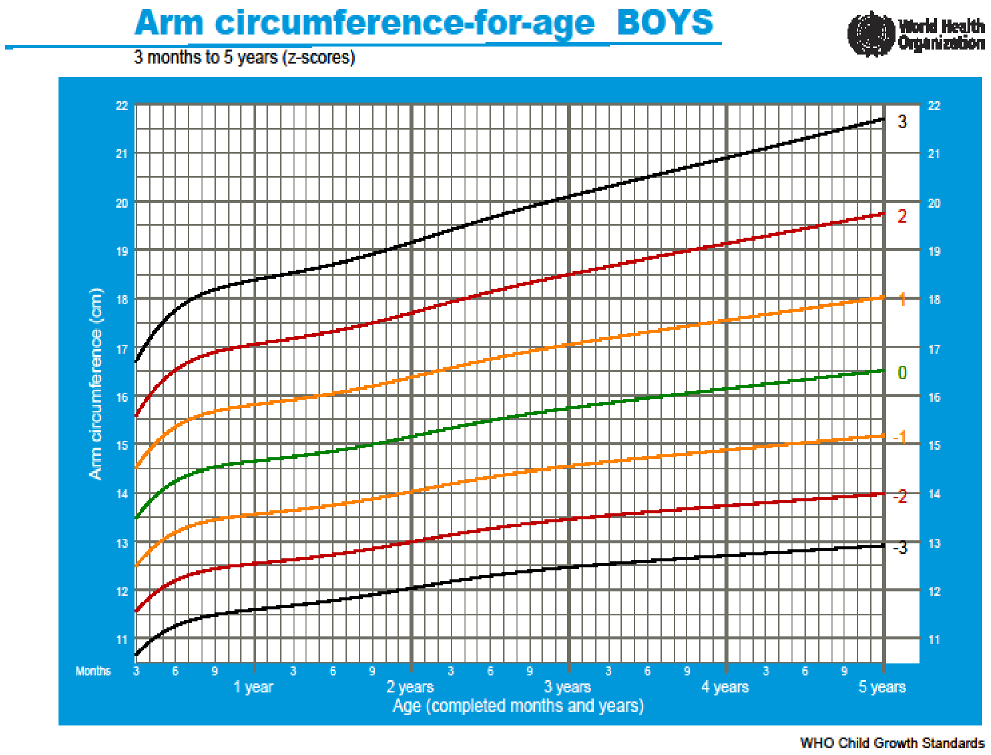

Source: WHO[14]

Figure C-5. MUAC-for-age Z-scores for Girls - 5 to 19 Years

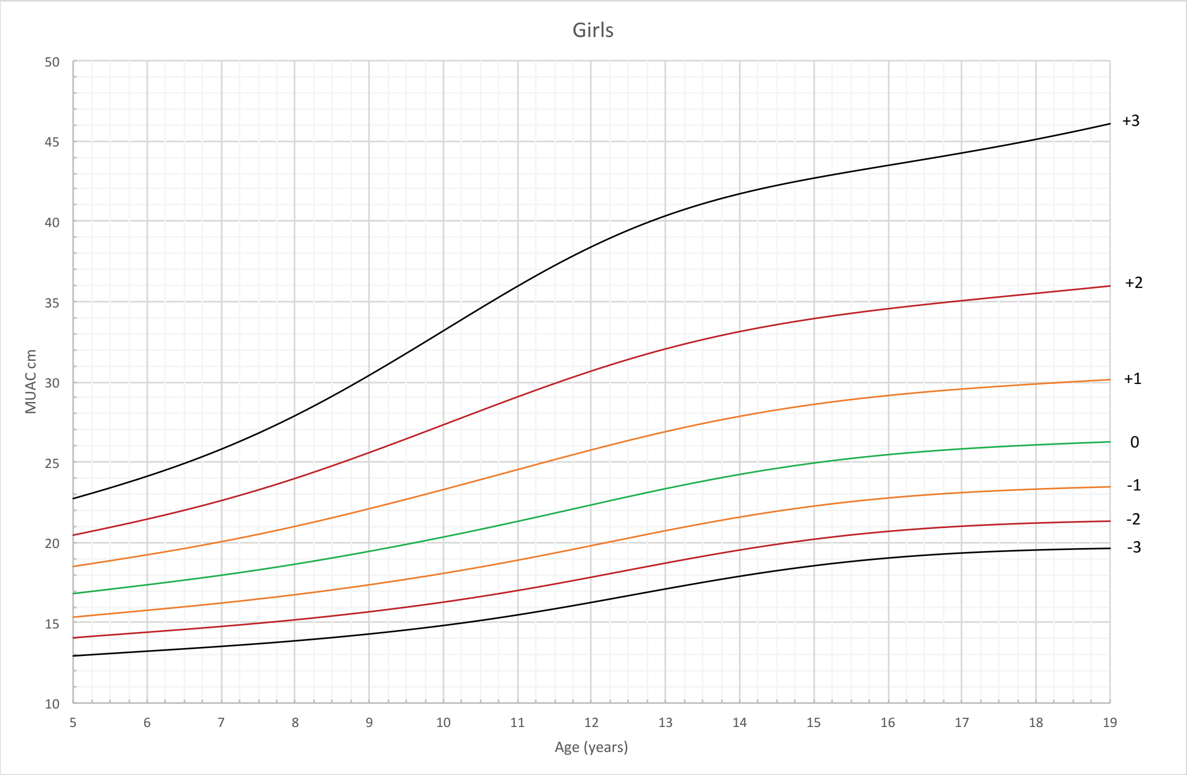

Source: Mramba[12]

Figure C-6. MUAC-for-age Z-scores for Boys - 5 to 19 Years

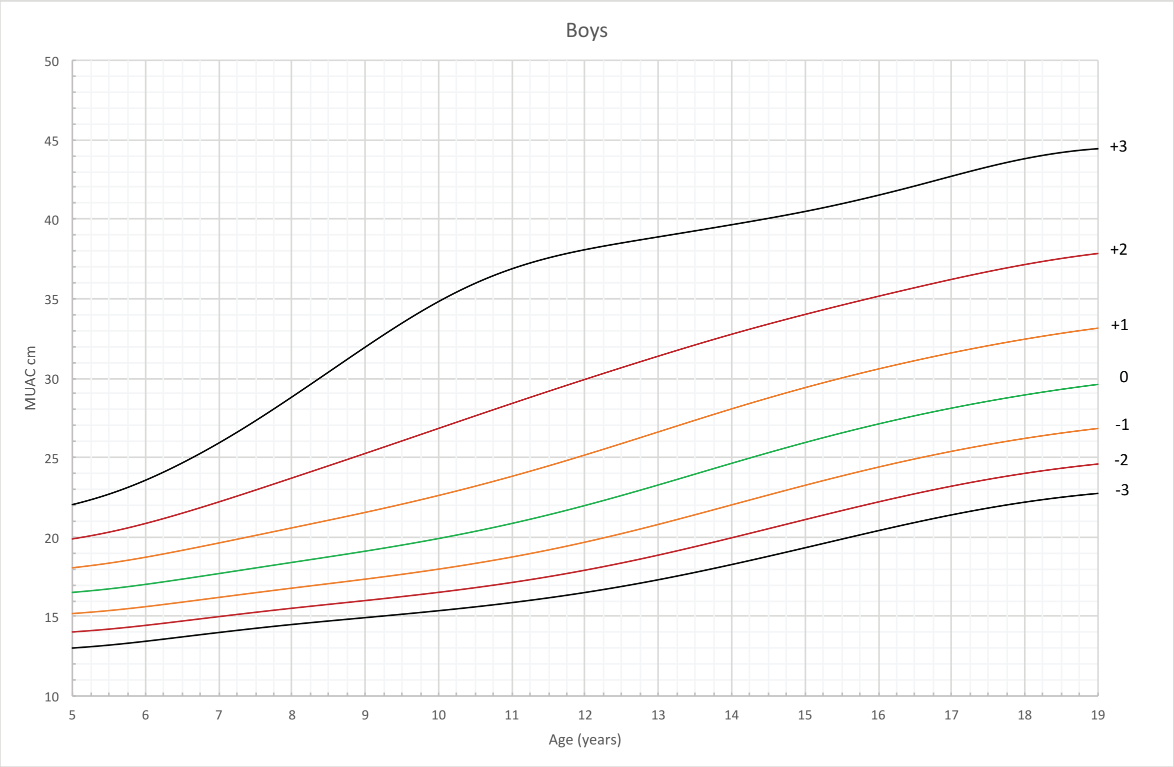

Source: Mramba[12]
