# Supplementary material for: Consensus recommendations for the nutritional management of children with cancer in limited resource settings: a report from the International Initiative for Pediatrics and Nutrition
Source: Front Nutr. 2025 Jun 26;12:1605632. doi: 10.3389/fnut.2025.1605632 (PMC12240747; doi:10.3389/fnut.2025.1605632)
Supplement: Supplementary file 2 [file Data_Sheet_2.pdf]

Nutritional Intervention:
A Training Manual for Pediatric Oncology

Second Edition

Contents

Introduction 5
Types of Pediatric Cancers and Associated Treatments 5
Acute Leukemias 5
Lymphomas 6
Brain Tumors 7
Solid Tumors 7
Neuroblastoma 7
Wilms Tumor 8
Soft Tissue Sarcomas of Skeletal Muscular Origin 8
Bone Tumors 8
Common Gastrointestinal Complications 9
Bowel Perforation and/or Obstruction 9
Constipation 9
Diarrhea 12
Gastrointestinal Hemorrhage 12
Mucositis/Stomatitis 13
Nausea/Vomiting 13
Neutropenic Enterocolitis/Typhlitis 13
Pancreatitis 13
Acute Kidney Injury (AKI) 14
Nutritional Assessment and Intervention 15
Nutritional Assessment (ABCDs of Nutrition Assessment) 15
Anthropometry 15
Biochemical Parameters 15
Clinical Evaluation 16
Dietary Assessment 16
Nutritional Intervention 19
Oral Interventions 19
Strategies for Nutritional Counseling 20
Enteral Tube Feeding 21
Types of Feeding Tubes 21

Placement of the Nasogastric Tube 23
Cleaning the Nasogastric Tube 24
Determining the Formula 24
Dosing, Rate, and Duration 25
Criteria for Monitoring Nutritional Intervention 27
Specialized Diets 28
Parenteral Nutrition (PN) 30
Severe Acute Malnutrition Management in the Pediatric Oncology Setting 32
Initial Treatment/Stabilization 33
Rehabilitation 33
Monitoring 33
Micronutrients 34
Collaboration with Other Disciplines 35
References 36

Abbreviations

AKI	acute kidney injury
ALL	acute lymphoblastic leukemia
ALT	alanine aminotransferase
AMDR	Acceptable Macronutrient Distribution Range
AML	acute myeloid leukemia
AST	aspartate aminotransferase
BUN	blood urea nitrogen
cm	centimeter
CNS	central nervous system
CT	computed tomography
dL	deciliter
DRI	Dietary Reference Intake
EN	enteral nutrition
g	gram
G-tube	gastrostomy tube
GI	gastrointestinal
MCT	medium chain triglyceride
mEq	milliequivalent
mg	milligram
mL	milliliter
mm	millimeter
mmol	millimole
MRI	magnetic resonance imaging
MUAC	mid-upper arm circumference
ND	nasoduodenal
NG	nasogastric
NHL	non-Hodgkin lymphoma
NJ	nasojejunal
NPO	nothing by mouth (Latin: *nil per os*)
NV	nausea/vomiting
PEG	percutaneous endoscopic gastrostomy
PN	parenteral nutrition
RDA	Recommended Dietary Allowance

GIR	glucose infusion rate
GJ-tube	gastro-jejunostomy tube
HIC	high-income country
HIV	human immunodeficiency virus
ILE	lipid injectable emulsion
IU	international unit
kg	kilogram
LMIC	low- and middle-income country
MAM	moderate acute malnutrition
mcg	microgram
RUTF	ready-to-use therapeutic food
SAM	severe acute malnutrition
SD	standard deviation
SES	socioeconomic status
tbsp	tablespoon
TPN	total parenteral nutrition
UL	Tolerable Upper Intake Level
UNICEF	United Nations Children's Fund
WBC	white blood cell
WHO	World Health Organization

Introduction

Nutritional interventions are often challenging to implement in a child with cancer due to the multiple factors concurrently impacting appetite and dietary intake. The primary goal of nutritional intervention in a child with cancer is to sustain and promote normal growth and development while the child is receiving the necessary anticancer therapy. Historically, clinicians were primarily concerned with maintaining optimal weight and preventing nutritional deficiencies. However, with the increasing prevalence of obesity, most clinicians are faced with balancing both ends of the nutritional spectrum—under- and over-nutrition. Nutritional intervention should be proactive so as to prevent the development of under-nutrition in patients at high risk of becoming nutritionally depleted, rather than being reactive and targeting the reversal of under-nutrition only when it becomes apparent. The same clinical goal holds for obesity, where prevention is a key approach for avoiding its onset altogether; this is especially relevant for children with acute lymphoblastic leukemia (ALL) and certain types of brain tumors. The most appropriate nutritional intervention must meet nutritional requirements while also addressing side effects impacting dietary intake, such as mucositis, severe nausea/vomiting (NV), gastrointestinal (GI) obstruction, constipation, and diarrhea. A family-based approach to nutritional counseling is generally regarded as optimal, as parents or guardians are essential to providing appropriate nutrition to the infant or child throughout the course of therapy. Importantly, an awareness of culturally driven food choices is essential for providing effective advice to diverse patient populations. Finally, in many resource-constrained settings, the socioeconomic status (SES) of the parents or guardians can impact their ability to sustain adequate nutrition at home and must be taken into consideration. Reliance on a dietitian to provide support and education to staff, families, and patients is a crucial component of optimal nutritional care.

Types of Pediatric Cancers and Associated Treatments

Acute leukemias, lymphomas, brain tumors, and other solid tumors such as neuroblastoma and Wilms tumor are the principal cancers observed in pediatric oncology. The incidence of a specific disease often correlates with age; for example, neuroblastoma is most commonly seen in infants and toddlers, while ALL is most commonly seen in school-age children and osteogenic sarcoma is primarily a disease of adolescence.[1] Treatment modalities include surgery, chemotherapy, radiation therapy, and/or biological therapies.

Acute Leukemias

Acute leukemias are cancers of the blood and represent the most common cancer category in children. Leukemias are categorized by the type of blood cell involved. In contrast to adult leukemias, nearly all childhood leukemias are of acute onset. The majority (i.e., 75-80%) of childhood leukemias are ALL, classifying it as the most common cancer in children in many parts of the world. The second most common is acute myeloid leukemia (AML). Both ALL and AML can occur throughout childhood and adolescence. ALL shows a peak of incidence among young school-age children.

The presenting signs and symptoms of acute leukemias include fatigue, pallor, bleeding, fever, lymphadenopathy, and bone pain. The diagnosis can be suspected by a review of the peripheral blood smear, and confirmed by a bone marrow aspirate and biopsy. Risk stratification in ALL depends first on age and initial white blood cell (WBC) count, with an age of 1 to 9 years and a WBC count of less than 50,000 per microliter considered favorable (i.e., standard or low risk). Children aged 10 years or older

at diagnosis and/or with an elevated WBC count are categorized as high risk. Other factors used to risk stratify are lineage (B or T cell disease), the involvement of the central nervous system (CNS) as determined through lumbar punctures, cytogenetic characteristics of the leukemia, and measurements of early response to induction therapy. Infants less than 12 months with ALL have a particularly poor prognosis and are usually treated on separate protocols.[2] Importantly, childhood ALL has an excellent cure rate in high-income countries (HICs), exceeding 90% overall. Cure rates in low- and middle-income countries (LMICs) vary widely depending on the access to and quality of treatment but are generally lower—sometimes dramatically so.

ALL treatment begins with an induction phase consisting of three to four chemotherapies, with some duration of hospitalization, and aims to achieve remission in one month. A prolonged post-induction period lasts 6 to 12 months and consists of moderate-intensity chemotherapy, mainly delivered in the outpatient setting. The duration and specifics vary by risk group and protocol. Finally, the maintenance phase of therapy is a prolonged phase of low-intensity treatment involving mainly oral chemotherapy taken at home. Compliance with oral therapy has been shown to be crucial in preventing relapse. The total duration of therapy is generally 2 to 3.5 years depending on the protocol and, in some cases, sex. A small number of children with particularly high-risk ALL may undergo bone marrow transplant as part of their initial treatment.

The treatment of AML is more intensive, requiring prolonged hospitalization. Therapy is based on cycles of high-intensity chemotherapy, resulting in marrow aplasia lasting for weeks. Infection rates are very high in children with AML, and deaths due to infection are a major risk. High-quality supportive care (nutrition, infection management, etc.) is crucial. Patients with high-risk disease, based on either cytogenetics or a lack of response, undergo bone marrow transplant. The total duration of therapy is generally 5 to 7 months. In HICs, the cure rate is between 65% and 70%. The treatment of AML is a major challenge in LMICs, as the supportive care needed to safely deliver the high-intensity treatments necessary to cure AML is often unavailable.

Lymphomas

The lymphomas are subdivided into two groups: Hodgkin and Non-Hodgkin lymphoma (NHL). Hodgkin lymphoma is seen primarily in older children and adolescents, and it typically presents with lymph node enlargement in the neck and/or chest. Epstein–Barr virus infection has long been associated with certain subtypes.[3] Advanced-stage patients have the additional involvement of the spleen, abdominal lymph nodes, and elsewhere. “B symptoms” of weight loss, fevers, and night sweats are less common in children compared to adults. All patients receive chemotherapy, while radiation therapy is reserved for higher risk patients or those with a delayed response to chemotherapy. Current treatment protocols emphasize risk-adapted strategies, based on sex differences in susceptibility to late effects. For example, males have a greater risk of infertility following treatment with alkylating agents, while females are more susceptible to second malignant neoplasms following irradiation to the chest. These late effects may be lessened by risk-adapted treatments that minimize exposure to specifically harmful modalities.

Pediatric NHL is a group of disorders including Burkitt, lymphoblastic, diffuse large cell, and other less common subtypes. The low-grade, indolent lymphomas of adults are typically not seen in children. Demographic features include a male preponderance, an association with immunodeficiency syndromes including human immunodeficiency virus (HIV) infection, and organ transplantation. Compared to Hodgkin lymphoma, the age at presentation of NHL extends to toddlers, and, in addition

to lymphadenopathy, there may be visceral and bone marrow or CNS involvement. The treatment is similar to that for ALL although generally shorter in duration. Cyclophosphamide (Cytoxan®) is a key agent, and radiotherapy is generally not required. Rituximab (Rituxin®) is a monoclonal antibody targeting a B lymphocyte antigen expressed in diffuse large cell lymphomas.

Brain Tumors

Primary CNS tumors are a diverse group of disorders that, together, represent the most common types of pediatric solid tumors. In HICs, pediatric brain tumors are the leading cause of both cancer-related morbidity and mortality.[1] CNS tumors occur throughout childhood. About 60% are located within the posterior fossa (i.e., the brainstem and cerebellum, located in the back of the head), commonly presenting with signs of hydrocephalus (i.e., fluid buildup within the brain leading to headaches, vomiting, and lethargy) and cranial nerve dysfunction. About 40% arise in the cerebrum (i.e., the large upper region of the brain), often causing symptoms such as headache, weakness, endocrine disturbance, and seizures. Diagnostic evaluation includes a detailed history, neurologic and funduscopic examination, imaging (i.e., magnetic resonance imaging [MRI] or computed tomography [CT]), and, in rare cases, lumbar puncture for cerebrospinal fluid cytology and/or tumor marker determination.

While there are more than 100 different brain tumor types, the most common are glioma (~55%), medulloblastoma (~20%), and ependymoma (~10%). While childhood brain tumors can metastasize within the brain and spine (i.e., the CNS), they rarely metastasize to other parts of the body. The mainstay of treatment consists of surgery, chemotherapy, and radiation, which, depending on the child's age, tumor location, and type of brain tumor, may be used in variable combinations. For example, a child with low-grade glioma can be cured with surgery only, if the tumor occurred in an area of the brain where resection is feasible (e.g., the cerebellum). However, if a low-grade glioma arises within the optic pathway or brainstem, where resection cannot be achieved, chemotherapy is used. Medulloblastoma, the most common malignant tumor in childhood, requires a combination of surgery, chemotherapy, and radiation for the best chance of a cure.

Solid Tumors

Neuroblastoma

Neuroblastoma is the most common malignancy of infants less than 1 year old and the most common extracranial solid tumor of childhood, accounting for 7% of childhood cancers. Nearly all cases are diagnosed by age five. Neuroblastoma arises from nerve cells originating in the neck, chest, abdomen, or pelvis. Infants often present with localized tumors, occasionally discovered on maternal ultrasound studies, while dissemination is common beyond the age of one year. The primary tumor is most often located in the adrenal gland and may grow to a large size, crossing the midline. Bone metastases may cause limping or the refusal to bear weight, along with the ecchymotic discoloration of the orbits (i.e., "raccoon eyes"). Paraspinous tumors may cause spinal cord compression with extremity weakness.[4]

Prognostic factors include age, stage, and pathologic features. Infants and children with localized tumors (i.e., low risk) are treated with surgery alone, which is curative in 90% to 100% of cases in HICs.[4] Intermediate-risk patients (i.e., mainly infants with regionally extensive tumors) receive moderately intensive four-drug chemotherapy, with survival rates exceeding 80%. Patients with high-risk neuroblastoma (i.e., mostly children over one year with histologically aggressive tumors metastatic

to the bone, bone marrow, lymph nodes, and liver) generally undergo confirmatory biopsy before receiving dose-intensive chemotherapy, followed by the attempted surgical resection of the tumor; they are then further treated with radiation to the sites of disease, and high-dose chemotherapy with autologous stem cell rescue.

Wilms Tumor

Wilms tumor, a cancer of the kidney, accounts for 6% of childhood cancers. The peak incidence is in children ages two to three years. The diagnosis is unusual beyond age 12, when primary renal tumors are more likely to be renal cell carcinomas. Wilms tumor has interesting associations with syndromes of hemihypertrophy (including Beckwith–Wiedemann syndrome), cryptorchidism, hypospadias, and aniridia. Cases are classified pathologically as “favorable histology” and “unfavorable histology,” with unfavorable histology including focal or diffuse anaplasia and/or clear cell sarcoma. The presenting signs and symptoms include asymptomatic or painful abdominal mass, painless hematuria, hypertension, and malaise. Treatment is based on staging after initial surgical exploration and attempted resection, the determination of histology, and the completion of a metastatic evaluation. Therapy includes nephrectomy for all patients, two-drug chemotherapy for Stages I and II, the addition of a third drug and abdominal irradiation for Stage III, and a combination of radiotherapy and multiagent chemotherapy for Stage IV (i.e., metastatic disease).

Soft Tissue Sarcomas of Skeletal Muscular Origin

Rhabdomyosarcoma is the most common of the soft tissue sarcomas. There are two age peaks: age two to four years, and early to mid-adolescence. Although a small proportion are associated with neurofibromatosis type 1 and Li-Fraumeni syndrome, most cases occur sporadically, with no known risk factor.[5]

There are two histological variants: embryonal and alveolar as well as several characteristic cytogenetic abnormalities.[5] Rhabdomyosarcoma generally presents as a painless lump with signs and symptoms varying by site: orbital (proptosis without erythema); parameningeal (dysphagia, “sinusitis,” jaw pain, and proptosis); and extremity (painless lump, urinary frequency, obtrusive hematuria, scrotal mass, and grape-like protruding vaginal mass). Treatment requires a multimodality approach and is based on staging. Treatment includes surgery / lymph node dissection with resection as a maximum non-mutilating, function-preserving operation and chemotherapy with or without radiation. The prognosis is related to the site of origin, tumor burden, resectability, presence of metastases, and histopathology.

Bone Tumors

Bone tumors account for 6% of childhood malignancies. They are most common in the second decade of life. The two most common types of bone cancer are osteogenic sarcoma and Ewing sarcoma. In osteogenic sarcoma, the clinical symptoms are swelling and pain in the affected area. The patient may present with a pathological fracture of the affected bone. Treatment consists of surgery (i.e., limb salvage or amputation) and chemotherapy. A biopsy is performed to establish the diagnosis, followed by neoadjuvant chemotherapy, with definitive surgery later. Neoadjuvant chemotherapy (i.e., the treatment given before surgery) may render the primary tumor resectable, reduce the risk of metastases, and provide important prognostic pathological information relating to the response to chemotherapy.[6] The lungs and bones are the most common sites for metastatic disease.

Ewing sarcoma of the bone accounts for 4% of childhood and adolescent cancers. The incidence among Caucasian is six times higher than it is among African American children, and the disease is rare in Chinese children. The most common primary sites are the pelvis, proximal bones of the extremities, upper tibia, and ribs. Common sites of metastases are the lung, bone, and bone marrow. Clinical symptoms at the time of presentation include pain, soft tissue swelling, and symptoms related to the site of origin. Constitutional symptoms of low-grade fever and weight loss may occur in some patients. Treatment consists of neoadjuvant chemotherapy, surgical resection, and/or local radiotherapy occurring after induction chemotherapy.

Common Gastrointestinal Complications

There are many side effects related to both chemotherapy and radiation that affect the GI tract. An overview of many of the conditions may be found below. Additional information and examples of several side effects may be found in Appendix Table 1.

Bowel Perforation and/or Obstruction

The invasion of NHL into the bowel itself (especially in Burkitt lymphoma) can present with bowel perforation or blockage. The early commencement of chemotherapy can resolve the invasion of the bowel wall, but intussusceptions may require surgery and chemotherapy. Some solid tumors of the abdomen can present with bowel obstruction, such as Wilms tumor and neuroblastoma. The obstruction is often due to extrinsic pressure causing the occlusion of the bowel, which tends to occur during advanced stages. This is treated by the initial surgical removal of the tumor followed by chemotherapy and, sometimes, radiation therapy. Nutritional intervention is often required to address bowel perforation or obstruction. In many cases, the use of the GI tract may be limited, necessitating other forms of nutritional intervention such as total parenteral nutrition (TPN); refer to the section on parenteral nutrition (PN) for more information. Most often, high-calorie meals fed in small portions frequently (i.e., every 1–2 hours) may provide some nutrients until surgery relieves the pressure on the bowels.

Constipation

The definition of *constipation* is individualized because a “normal” bowel movement varies between patients. For one patient, one stool every three days may be defined as normal, whereas one to two stools per day may be typical for others. In children with cancer, a decrease in the frequency of stools or the occurrence of hard stools that are accompanied by pain and discomfort can be caused by chemotherapeutic agents, intestinal obstruction, spinal cord compression, electrolyte imbalance, pain medication, a lack of movement/exercise, or a low-fiber diet.

Increasing fiber intake may help relieve constipation. To determine the minimum daily fiber intake for children older than two years, the child’s age in years plus five is equal to the recommended daily grams of fiber.[7] This can be achieved through a balanced diet containing whole grains, fruits, and vegetables, and increased fluid intake. Table 1 provides a list of foods rich in soluble and insoluble fiber. Fiber supplements can help manage constipation; however, the effectiveness of a fiber supplement will depend on the viscosity of the soluble fibers. High-viscosity fiber (e.g., gel-forming fiber, such as beta-glucans, psyllium, and guar gum) retains water and resists hydration, whereas insoluble fiber mechanically irritates the gut mucosa to stimulate water secretion; the latter may be particularly

helpful during constipation. Table 2 provides examples of sources of both forms of fiber.[8] Several lifestyle strategies, including increased fluid intake and mobility, can augment the effectiveness of fiber supplementation. It is important to always increase water intake as fiber intake increases. Additional information has been described elsewhere.[9]

Table 1. Food sources of insoluble and soluble fiber

	Fiber content		
Food	Total fiber (g / 100 g serving)	Soluble fiber (g / 100 g serving)	Insoluble fiber (g / 100 g serving)
Grains			
Instant sorghum porridge, cooked[a]	9.1	3.1	6.0
Whole-wheat bread[b]	6.0	1.3	4.7
Corn tortilla[b]	5.5	1.1	4.4
Pearl barley, cooked[c]	5.5	1.6	3.9
Rye bread[b]	4.5	1.6	2.9
Quinoa, cooked[c]	3.6	0.7	2.8
Brown rice (long grain), cooked[b]	3.3	0.4	2.9
Buckwheat, cooked[c]	2.3	0.3	2.0
Rolled oats, cooked[c]	2.1	0.9	1.2
Instant grits (boiled cornmeal), cooked[b]	1.6	0.1	1.5
Millet, cooked[c]	1.3	0.2	1.0
Fruit			
Guava, raw[b]	13.4	1.5	11.8
Figs, dried[c]	9.8	1.5	8.3
Prunes[b]	8.1	4.5	3.6
Avocado (Florida or Fuerte), raw[b]	6.8	1.3	5.5
Pear with skin, raw[b]	3.2	0.9	2.3
Raisins[b]	3.1	0.9	2.2
Kiwi, raw[c]	3.0	0.7	2.3
Plum with skin, raw[b]	2.9	1.1	1.8
Peaches with skin, raw[b]	2.8	1.3	1.5
Oranges, raw[b]	2.4	1.4	1.0
Apple with skin, raw[b]	2.2	0.7	1.5
Nectarine with skin, raw[b]	2.1	1.0	1.1
Peaches without skin, raw[b]	2.0	0.8	1.2
Bananas (yellow), raw[b]	1.8	0.6	1.2
Mango, raw[b]	1.8	0.7	1.1
Pineapple, raw[b]	1.4	0.0	1.4
Legumes			
Split peas, cooked from dry[b]	10.7	0.1	10.6
Red kidney beans, canned[b]	7.1	1.4	5.7
Pinto beans, canned[b]	6.7	1.0	5.7
Chickpeas, canned[b]	6.2	0.4	5.8
Lentils, cooked from dry[b]	5.9	0.5	5.4
Cowpeas, canned[b]	4.5	0.4	4.1

Vegetables			
Broccoli, raw[b]	3.5	0.4	3.1
Spinach, raw[b]	3.2	0.8	2.4
Carrots, raw[b]	2.9	0.5	2.4
Cauliflower, raw[b]	2.6	0.5	2.1
Green cabbage, raw[b]	2.3	0.5	1.8
Onion, raw[b]	1.9	0.7	1.2
Sweet green peppers, raw[b]	1.5	0.5	1.0
Red tomatoes, raw[b]	1.3	0.1	1.2
Cucumber with peel, raw[b]	1.1	0.2	0.9
Nuts and seeds			
Black chia seeds[d]	40.0	7.0	33.0
Flaxseeds[d]	20.0	7.0	13.0
Grated and dehydrated coconut[c]	16.3	1.4	13.3
Macadamias[e]	15.9	0.1	15.8
Almonds[e]	15.2	0.8	14.4
Peanuts[e]	10.9	1.0	9.9
Pistachios[e]	10.6	0.3	10.3
Pecans[e]	9.4	0.4	9.1
Pumpkin seeds[e]	8.5	1.2	7.3
Hazelnuts[e]	8.2	0.4	7.8
Walnuts[e]	6.1	0.8	5.3
Pine nuts[e]	4.5	0.8	3.8

Abbreviations: g, gram.
Sources: [a]Haliza W, Widowati S. The characteristic of different formula of low tannin sorghum instant porridge. *IOP Conf Ser: Earth Environ Sci.* 2021;653:012124. [b]Li BW, Andrews KW, Pehrsson PR. Individual sugars, soluble, and insoluble dietary fiber contents of 70 high consumption foods. *J Food Compos Anal*. 2002;15:715-723. [c]Fuller S, Tapsell LC, Beck EJ. Creation of a fibre categories database to quantify different dietary fibres. *J Food Compos Anal*. 2018;71:36-43. [d]United States Department of Agriculture. FoodData central. Updated October 28, 2022. Accessed November 2, 2022. https://fdc.nal.usda.gov. [e]Lintas C, Cappelloni M. Dietary fiber content of Italian fruit and nuts. *J Food Comp Anal.* 1992;5:146-151.

Table 2. Common fiber supplements

Properties	No water-holding capacity				
	Insoluble		Soluble, non-viscous		
	Wheat bran	Cellulose	Wheat dextrin	Inulin	Polydextrose/ oligosaccharides
Examples	*Food sources*: All-Bran®[a] cereal, whole-grain wheat[10]	Ciruelax®[b] *Food sources*: prunes, root and leafy vegetables, legumes, pears, apples[11]	Benefiber®[c] Fibre Clear®[d] Heat- or acid-treated wheat[10]	Fiber Choice®[e] Fiber Mais®[e] Chicory root[10]	Hyfiber®[f] Gadavyt Liquid Fiber®[g] *Food sources*: white onion, scallion, Jerusalem artichoke, asparagus, soybeans, chicory root, garlic[12]
Degree of fermentation	Poorly fermented	Poorly fermented	Readily fermented	Readily fermented	Readily fermented

Properties	Water-holding capacity				
	Soluble, viscous	Soluble, viscous / gel-forming			
	Methylcellulose	Partially hydrolyzed guar gum	Beta-glucans	Psyllium	Pectin
Examples	Citrucel®[h]	Fiber Mais®[e]<br>*Food sources*: guar or cluster beans[10]	Dietary Fibre Powder®[i]<br>*Food sources:* oats, barley[10]	Metamucil®[j]<br>Fibra-Flat®[k]<br>Dietary Fibre Powder®[i]	Ciruelax®[l]<br>*Food sources*: prunes, citrus fruits, apples[11]
Degree of fermentation	Non-fermented	Readily fermented	Readily fermented	Non-fermented	Readily fermented

[a]Kellogs, [b]Garden House, [c]Novartis, [d]Brunel Laboratories, [e]Nestle, [f]Medtrition, [g]Nutritech, [h]GSK, [i]Tony Furgerson, [j]Procter & Gamble, [k]Global Farma, [l]Garden House.

Source: Adapted from McRorie JW, Jr., McKeown NM. Understanding the physics of functional fibers in the gastrointestinal tract: an evidence-based approach to resolving enduring misconceptions about insoluble and soluble fiber. *J Acad Nutr Diet*. 2017;117(2):251-264.

Diarrhea

Diarrhea is a common side effect that can be caused by an infection, antibiotics, treatment (e.g., chemotherapy or radiation to the GI tract), or micronutrient deficiency (e.g., zinc); it is also associated with severe acute malnutrition (SAM). *Acute diarrhea* is usually defined as three or more loose stools in a 24-hour period, whereas *persistent diarrhea* is defined as diarrhea lasting more than 14 days. The final day of diarrhea is usually identified as the last day meeting the above definition, followed by 48 hours without diarrhea.[8] Several chemotherapy agents can cause diarrhea, including cisplatin, cyclophosphamide, doxorubicin, irinotecan, and methotrexate, as can molecularly targeted agents, radiation to the abdomen, and stem cell transplantation. Patients at risk of developing diarrhea associated with zinc deficiency are those strictly adhering to a vegetarian diet (i.e., no meat) and those from regions where the endemic prevalence of zinc deficiency is high.

For zinc-deficient diarrhea, the World Health Organization (WHO) recommends low-concentration oral rehydration salts along with zinc supplementation.[13] The recommended zinc dosage is 20 mg per day for children older than 6 months, or 10 mg per day for those younger than 6 months, for 10 to 14 days. However, these recommendations should be balanced with the interventions occurring during conventional chemotherapy. Clinical studies suggest the limited efficacy of zinc supplementation in children without zinc deficiency. For most other cases of diarrhea, gel-forming fibers may be beneficial (Table 2).

Gastrointestinal Hemorrhage

GI hemorrhage is a severe and potentially life-threatening side effect of cancer therapy. It may occur due to bowel infections, enterocolitis, ulcers, primary bowel tumors, graft-versus-host disease, complications of thrombocytopenia, and coagulation defects. Its presentation is varied and can include abdominal pain and distension, hematemesis, and melena (i.e., dark, sticky feces), as well as signs and symptoms associated with acute blood loss. The bleeding may be diffuse or localized and may require endoscopy and radiological imaging to ascertain the source. Supportive care with appropriate blood products and possible surgical intervention is required. Enteral feeding is contraindicated until the cessation of bleeding and the bowel is no longer showing clinical signs of ileus. The time to feeding

following GI hemorrhage is dependent on the etiology, severity, and risk of rebleeding.[14] The decision to initiate feeding must be directed by the patient's pediatrician and/or pediatric oncologist.

Mucositis/Stomatitis

Mucositis (also known as *stomatitis*) is a common side effect of intensive chemotherapy, most frequently seen within several days after the administration of anthracyclines, high-dose methotrexate, or radiation involving the head and neck or bowel. The GI mucosa contains rapidly dividing cells, which are preferentially affected by chemotherapy due to their rapid turnover. The mucositis may be limited to the mouth and esophagus, but it sometimes may extend into the entire bowel. Mucositis is associated with significant pain and the impairment of swallowing, which may lead to diminished oral nutritional intake. It is a portal of entry for bacteria; thus, sepsis is a risk in patients with severe mucositis. This is especially the case in children who are also neutropenic. Nutritional interventions for mucositis include avoiding acidic, spicy (although this may vary by region), or hot foods and drinks, as they may cause further irritation to the mucosa. In severe cases, the placement of a nasogastric (NG) tube may be indicated so as to ensure adequate dietary intake. The placement of an NG tube should be undertaken **prior** to the development of mucositis so as to avoid additional discomfort and the risk of further damage to the intestinal mucosa.

Nausea/Vomiting

At some point in therapy, most children will experience NV, which may not be entirely controlled with antiemetic agents. The emetic potential of most chemotherapy drugs has been classified into three categories: anticipatory NV (i.e., prior to chemotherapy administration), acute NV (i.e., during chemotherapy administration), and delayed NV (i.e., immediately or several hours after chemotherapy administration). Unfortunately, the prevention and treatment of NV is not always successful and often results in diminished nutritional intake. Most recently, an evidence-based clinical guideline has been developed for the diagnosis and management of NV in childhood cancer.[15] The dietary management of NV should entail consultation with a registered dietitian to include dietary strategies to optimize intake during periods of NV.

Neutropenic Enterocolitis/Typhlitis

Neutropenic enterocolitis, sometimes called *typhlitis* (i.e., inflammation of the caecum), is a severe complication of intensive chemotherapy and is often preceded by chemotherapy-induced gut mucositis. It is seen in prolonged neutropenia presenting with fever, severe abdominal pain, and, sometimes, diarrhea. In severe cases, it may lead to sepsis, due to gram-negative, gram-positive, and anaerobic bacteria, shock, and bowel perforation. Dietary management initially entails complete bowel rest, during which PN may be required (refer to section on PN). However, the strict avoidance of oral intake is controversial; some clinicians suggest that intake in small amounts may promote the healing process.[16]

Pancreatitis

Pancreatitis is a serious complication that most often occurs following the administration of L-asparaginase and cytarabine. It is also associated with stem cell transplantation and the prolonged use of TPN. The dietary management of pancreatitis is multidisciplinary and is directed by the patient's

pediatrician and/or oncologist. Its presentation includes severe abdominal pain, frequently radiating to the back, and elevated pancreatic enzymes (i.e., lipase and amylase) in the blood. Changes to the pancreas itself may also be observed with abdominal ultrasound or CT scan.

Acute pancreatitis can be mild, moderately severe, or severe.[17] *Mild acute pancreatitis* is defined as acute pancreatitis without organ failure or local or systemic complications, and it usually resolves within one week. *Moderately severe acute pancreatitis* is defined as the presence of either organ failure that resolves in less than 48 hours or local or systemic complications. Local complications include pancreatic complications such as fluid collection or necrosis, while systemic complications include the exacerbation of a previously diagnosed comorbid disease (e.g., kidney disease). *Severe acute pancreatitis* is defined as organ failure that persists for longer than 48 hours.

Historically, patients with acute pancreatitis were kept *nil per os* (NPO; i.e., nothing by mouth), with or without PN. However, recent research and guidelines support early enteral nutrition (EN) in children with mild acute pancreatitis.[18,19] Initiating a normal oral diet without restrictions within 48 hours of admission is well tolerated, is not associated with adverse pain outcomes, and may reduce the length of hospital stay.[18,19]

For severe acute pancreatitis, EN (i.e., either oral, NG, or nasojejunal [NJ] feeding) should be attempted within 72 hours of admission, once the patient is hemodynamically stable. EN is preferred over PN. However, a combination of EN and PN can be used in children who do not meet caloric goals with EN alone. Feeding into the jejunum should be reserved for those unable to tolerate oral or NG-tube feeding.

Children with recurrent acute pancreatitis should receive a regular diet in between episodes of pancreatitis, when tolerated.[18] In selected patients who are intolerant of a regular diet, abdominal pain and vomiting may improve on a low-fat diet. A regular diet can safely be started within one week after the onset of acute pancreatitis for cases other than those caused by hypertriglyceridemia.

For the treatment of acute recurrent pancreatitis caused by hypertriglyceridemia, a low-fat diet is required for both the treatment of hypertriglyceridemia and the prevention of recurrent pancreatitis.[18] While *hypertriglyceridemia* is defined as triglyceride levels exceeding the 95th percentile for age, the following classifications are used when triglyceride levels exceed 500 mg per dL[20]:

- **Very high**: triglycerides ≥ 500–999 mg per dL
- **Severe**: triglycerides ≥ 1000–1999 mg per dL
- **Very severe**: triglycerides ≥ 2000 mg per dL

Pancreatitis secondary to hypertriglyceridemia is typically seen when triglyceride levels exceed 1000 mg per dL.[20] The risk of acute pancreatitis increases to 5% with severe hypertriglyceridemia and increases further to 10% to 20% with very severe hypertriglyceridemia.

Acute Kidney Injury (AKI)

Acute kidney injury (AKI) is an abrupt decline in kidney function defined by a specified increase in serum creatinine or a decrease in urine output.[21] Risk factors for developing AKI include renal Burkitt's lymphoma, ALL, AML, lower kidney function at diagnosis, tumor lysis syndrome, and the administration of methotrexate.[22,23]

Energy metabolism is altered in patients with AKI due to the acute catabolic comorbidities and complications associated with AKI, which lead to increased energy requirements. According to expert

opinion, a caloric intake of 20% to 30% above the Dietary Reference Intake (DRI) will provide adequate calories in most children with AKI, without causing a significant risk of overfeeding and the associated complications.[24] Children with AKI experience increased protein catabolism, and thus, it is important to provide enough protein to optimize protein synthesis and the inflammatory response while preserving skeletal muscle. Small studies have shown that a protein intake of ≥ 80% of the estimated needs (0–2 years: 2–3 g protein/kg; 2–13 years: 1.5–2 g protein/kg; and > 13 years: 1.5 g protein/kg) in critically ill pediatric patients with AKI is not associated with a delay in renal recovery.[25] It has also been demonstrated that patients with AKI use more calories from fat and less calories from carbohydrates. Therefore, carbohydrates should make up 20% to 25% of calories, lipids 30% to 40%, and protein 40% to 50%.

Electrolyte abnormalities including hyponatremia, hyperkalemia, hyperphosphatemia, and hypocalcemia are common in AKI. Electrolytes should be medically managed, and dietary restriction of potassium and/or phosphorus may be needed. If dietary restriction of potassium and/or phosphorus is needed, evaluate the phosphorus and/or potassium content of the patient's diet and provide counseling on reducing dietary phosphorus and/or potassium intake. The bioavailability of potassium and phosphorus may also be considered when providing counseling. Potassium added to processed foods is 90% to 100% absorbed, whereas potassium from unprocessed fruits and vegetables is 60% absorbed.[26] Similarly, phosphorus bioavailability is highest from processed foods (up to 100% of phosphorus is absorbed), while only 40% to 60% of phosphorus found in whole foods is absorbed.[27] Processed foods that contain potassium or phosphorus additives differs by country. For infants, breastmilk is lower in phosphorus and potassium compared to cow's milk or infant formulas.[26,27]

Nutritional Assessment and Intervention

A nutritional assessment of a child diagnosed with cancer should include the "ABCDs": 1) anthropometry (refer to IIPAN manual *Nutritional Assessment: A Training Manual in Anthropometry*), 2) biochemical assessment, 3) clinical evaluation, and 4) dietary history. This includes an evaluation of the medical and surgical history, anticipated oncology treatments, and expected toxicities from treatment. Nutritional therapies may include oral interventions, EN, and PN. Specialized diets may also be indicated, particularly education on the neutropenic diet.[28] Advanced biochemical tests are usually not available in LMIC settings. When indicated, the use of EN is preferred over PN due to its proven efficacy while decreasing the risk of infections and cost. Importantly, EN are critical for enhancing the gut's immunity through effects on the microbiome.

Nutritional Assessment (ABCDs of Nutrition Assessment)

Anthropometry

Refer to the IIPAN manual *Nutritional Assessment: A Training Manual in Anthropometry.*

Biochemical Parameters

Biochemical parameters may be used in determining the nutritional status of a child with cancer. Serum prealbumin and albumin levels may be obtained to determine visceral protein status, although hydration status, inflammation, and liver function will affect these values. Prealbumin levels may also be affected by renal disease and vary according to age. Because the turnover rate is two to three days for

prealbumin—versus 21 days for albumin—prealbumin reflects the effects of recent interventions. The availability of this test depends on the institutional laboratory facilities. Other laboratory measures may be used to monitor organ function. Appendix Table 2 provides an overview of common laboratory values obtained in pediatric oncology, normal ranges, and the clinical interpretation from a nutritional perspective.

Nutritional diagnostics should be performed to aid in the detection and remediation of macro- and micronutrient deficiencies and to monitor for nutrition-related toxicities such as hypertriglyceridemia or hyperglycemia. Advanced biochemical tests are usually unavailable in LMICs but are not always an essential component of nutritional assessment. Screening for adequate dietary intake of vitamin A, vitamin D, folate, iron, or zinc are especially important in areas where deficiencies are endemic to the region and tests are unavailable. The combination of a clinical examination and dietary recall can be especially helpful when laboratory tests are unavailable.[29] The signs and symptoms of nutrient deficiencies (see Appendix Table 3) obtained from the clinical examination may be confirmed with laboratory tests, if available. Additionally, it is essential to assess for comorbidities that can result in under-nutrition, such as tuberculosis, HIV-related disease, and parasitic infections.

Clinical Evaluation

A nutrition-focused clinical examination is easy, free of cost, and may be performed by any trained provider. A nutritional clinical examination should consider the cancer diagnosis, anticipated exposure to chemotherapy/radiation, expected toxicities (side effects), and screening for nutritional deficiencies. Importantly, a clinical evaluation should consider food security, family access to food public assistance programs, and familial factors that may impede the child's ability to maintain a healthy nutritional status (e.g., the use of traditional complementary/alternative medicine). Routine clinical assessments, including screening for the signs of severe vitamin and trace element deficiency, should be performed. The signs and symptoms of nutrient deficiencies are provided in Appendix Table 3. At a minimum, all children should be screened for SAM (refer to the section entitled SAM Management in Pediatric Oncology Setting).[30]

Dietary Assessment

Dietary assessment aids in determining daily caloric intake and whether additional screening is indicated for micronutrient deficiencies. For children placed on restrictive diets, such as a low-fat diet or specific mineral restrictions, food records (e.g., prospective collection of dietary intake) allow the clinician to monitor the child's intake during admission or in the outpatient setting. Dietary recalls (e.g., retrospective collection of dietary intake) are beneficial in determining if enteral feeding is indicated as well as in assessing dietary diversity, quality, and composition. Dietary analysis may be instrumental in assisting children in managing treatment-related toxicities that may be exacerbated by dietary intake, such as diarrhea or constipation. Follow-up assessments of nutritional status for every child should preferably occur at every outpatient visit and more frequently in the inpatient setting. Daily follow-up is indicated in children with SAM, those with moderate acute malnutrition (MAM), and in those experiencing any condition or side effect affecting their ability to consume the recommended calorie or protein intake.

Several reference values may be utilized to ascertain diet. IIPAN recommends the use of the DRIs set forth by the Institute of Medicine of the National Academies in the United States.[7,31,32] For

macronutrients, the Acceptable Macronutrient Distribution Ranges (AMDRs), as a percentage of total calories, can serve as a reference for dietary composition.[7] For children 4 years of age and older, the AMDR is 45% to 65% carbohydrate, 10% to 30% protein, and 25% to 35% fat. The percentages for children younger than 4 years old are 45% to 65% carbohydrate, 5% to 20% protein, and 30% to 40% fat. For micronutrients, dietary intakes are classified as "under" (i.e., intake below the Recommended Dietary Allowance [RDA]), "met" (i.e., intake within the RDA), and "over" (i.e., intake exceeding the RDA). These values consist of the four indices listed below. References for estimating calories, protein, fluids, and micronutrients can be found in Appendix Tables 4–12.

The DRI is a system of nutrition recommendations inclusive of four values that vary by age and sex. The two values used in clinical practice are as follows:

- **Recommended Dietary Allowance (RDA)**: the average daily level of intake sufficient to meet the nutrient requirements of nearly all (i.e., 97–98%) individuals
- **Tolerable Upper Intake Level (UL)**: the maximum daily intake unlikely to cause adverse health effects

The DRIs have been established for nutrients that are considered essential (i.e., vitamins, minerals, water, carbohydrates, protein, fat, linoleic acid, and linolenic acid). In clinical practice, the DRI and RDA can be used to calculate the estimated nutrition requirements of patients (e.g., calories, protein, or micronutrients). The UL can be especially useful when evaluating the safety of a nutritional supplement and ensuring that the dose does not exceed the safe upper levels.

24-Hour Recall

Before attempting to improve dietary habits or offer nutritional guidance, it is necessary to assess and evaluate usual daily food intake. A dietary intake of < 50% of the estimated energy needs over a 48-hour period has been shown to be a significant predictor of weight loss in hospitalized children[33]; however, there is no universal standard that defines *significant* weight loss (refer to IIPAN manual *Nutritional Assessment: A Training Manual in Anthropometry*). The WHO estimates of adequate protein intake and energy requirements suggest that a protein-to-energy ratio of < 5% for 2- to 5-year-olds is considered detrimental and is classified as protein deficiency.[34]

There are several methods for obtaining information on dietary intake, including a 24-hour diet recall or a 3- to 5-day food record. A 24-hour diet recall is an informal, qualitative method in which the patient or patient's guardian is asked to recall all the foods and beverages that were consumed in the last 24 hours (Table 3). This includes information on the quantities and methods of preparation. An advantage of this method is that dietary information is easily obtained and can be useful when no other nutritional data is available. To aid patients in recalling food items, the Multiple-Pass Method (Table 4) is a validated approach to help patients accurately recall dietary intake. The Multiple-Pass Method uses a five-step multiple-pass approach to collect dietary data. The first step is an unstructured, uninterrupted listing of all foods and beverages consumed. The next three steps use a structured approach to data collection including memory cues. The final probe step is an unstructured question about any other foods recalled and includes several additional memory cues.

Table 3. Example of a 24-hour recall

Date	Time	Food or drink	Portion
6/19/19	9:00 am	• Instant oatmeal (maple and brown sugar) made with whole milk	• 1 packet oatmeal (43 g)<br>• 120 mL milk

		• Orange juice	• 240 mL juice
6/19/19	10:30 am	• Greek yogurt (strawberry cheesecake flavor)<br>• Water	• 1 container (150 g)<br>• 150 mL water
6/19/19	1:00 pm	• Ramen noodle soup (chicken flavor); used full packet of seasoning that came with soup<br>• Orange juice	• ½ block of noodles (43 g)<br>• Boiled in 480 mL of water<br>• 120 mL juice
6/19/19	2:15 pm	• Ice cream (Belgian chocolate) with whipped cream (original)	• ¾ cup ice cream<br>• 5 tbsp whipped cream
6/19/19	6:00 pm	• Homemade baked French fries (with skin)<br>• Ketchup<br>• Homemade baked chicken wings (with skin), made with barbeque sauce<br>• Water	• 2 small potatoes<br>• 2 tbsp ketchup<br>• 5 medium-sized chicken wings<br>• 3 tbsp barbeque sauce<br>• 240 mL water
6/19/19	10:30 pm	• Shredded wheat breakfast cereal<br>• Whole milk	• 1 cup cereal<br>• 120 mL milk

Abbreviations: g, gram; mL, milliliter; tbsp, tablespoon.

Table 4. Multiple-Pass Method

Steps	Purpose
1. Quick list	To collect a list of foods consumed by the respondent in a 24-hour period. Cues given to respondent to help them think about the day's events and remember the foods eaten. Respondent uses own recall strategies.
2. Forgotten foods list	To obtain the additional recall of foods by focusing respondent's attention on foods that are often forgotten: nonalcoholic beverages, alcoholic beverages, sweets, savory snacks, fruit, vegetables, cheeses, breads and rolls, and any other foods items.
3. Time and occasion	To collect information on the time at which the respondent ate each food and the name of the eating occasion. Food is sorted into chronological order and grouped by eating occasion or meal (e.g., breakfast, lunch, or dinner).
4. Detail and review	To collect a detailed description of each food reported, which includes amount eaten, brand, cooking method, restaurant name, and whether it was eaten at home.
5. Final probe	Provides a final opportunity to recall foods. Cues are given related to when foods may be eaten and easily forgotten. Encourages reporting of small amounts of food that may have been regarded as not worth mentioning.

Food Records

A three- or five-day dietary record may be useful in identifying problems patients have in meeting the recommended intakes and are particularly beneficial if obtained over a longer span of days. A dietary record can be useful in identifying weaknesses in the patient's dietary intake and can provide a basis for recommendations. A food record lists all foods consumed during a period of three days, which can be divided into two weekdays and one weekend day. Parents or guardians record the types of food served to the child, the manner in which the food was prepared, the use of additional condiments, and the amount of food consumed using common household measurements. Every effort should be made to document if side effects from therapy have resulted in altered dietary intakes. There are several benefits to collecting a food record such as obtaining detailed information about the foods and beverages consumed on a given day or in a given period of days, a more accurate account of food quantity, and reduced recall bias.

Nutritional Intervention

Oral Interventions

The initial step in providing nutritional therapy begins with counseling the child and caregivers on the impact of cancer and its therapy on nutritional status. For children up to six months of age, the WHO and United Nations Children's Fund (UNICEF) recommend breastfeeding exclusively, which should also be encouraged in children with cancer. If breast milk is not available, use only infant formula suitable for the baby's age—do not feed a child younger than six months animal milk, water, or formula for older children. From the age of six months, children should begin eating safe and adequate complementary foods while continuing to breastfeed for at least two years (Table 5).

The child's favorite nutrient-dense foods and beverages should be encouraged when the child is able to eat with minimal difficulty. If accepted by the child, oral supplements offer another source of nutrients by augmenting caloric intake. In Tanzania, homemade smoothies made from local foods available at the hospital/home are utilized to support growth and development. The use of medically dense food products such as ready-to-use therapeutic food (RUTF) ensures the adequate intake of macro- and micronutrients.[30,35,36] In Malawi, RUTF was produced from local peanut butter, and there is evidence supporting its efficacy in promoting weight gain in children with cancer.[36] In cases of severe malnutrition, adaptation of the WHO guidelines for the management of SAM should be implemented upon diagnosis (refer to the section entitled SAM Management in Pediatric Oncology Setting).[30,37] Evidence supporting the use of appetite stimulants such as megestrol acetate has been of low quality; as such, their use should be considered very judiciously due to their modest effects on weight gain, largely attributable to disproportionate increases in fat accrual, and an increased risk of adrenal suppression.[38-40]

Table 5. Practical guidance on complementary foods for children 6 to 23 months of age

<table>
<tr><th>Age (months)</th><th>Texture</th><th>Frequency</th></tr>
<tr><td>6–8</td><td>Start with well-mashed foods and continue with mashed family foods</td><td>2–3 meals per day</td></tr>
<tr><td colspan="3">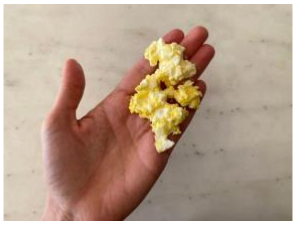
Hard-boiled egg mashed with water
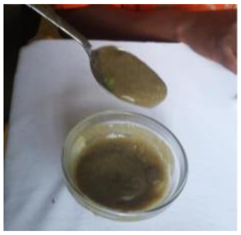
Porridge enriched with avocado
Note: Porridge can be enriched with a variety of foods including avocado, ripe banana, soya paste, or groundnut paste.
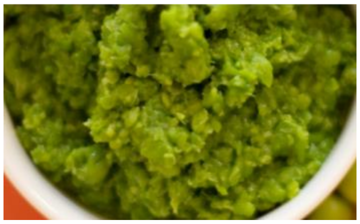
Mashed peas</td></tr>
<tr><td>9–11</td><td>Finely chopped or mashed family foods</td><td>3–4 meals per day</td></tr>
</table>

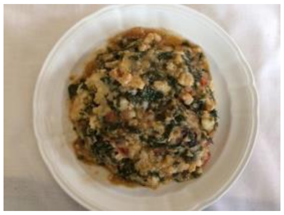<br>Mashed ugali with scrambled eggs and cooked vegetables	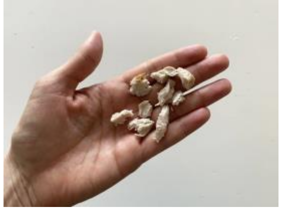<br>Finely chopped/shredded pieces of chicken	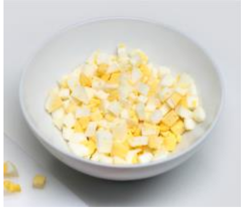<br>Finely chopped pieces of hard-boiled egg
12–23	Family foods, chopped or mashed if necessary	4–5 meals per day
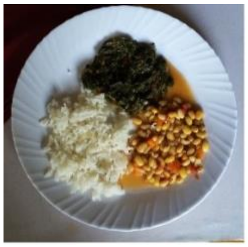<br>Soya stew served with lentils, vegetables, and rice	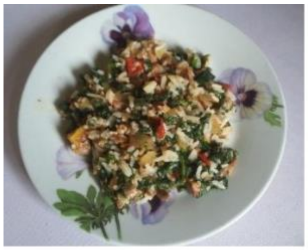<br>Vegetables mixed with rice and beef	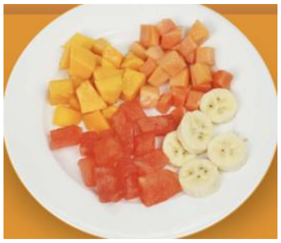<br>Various fruits cut into finger foods

Image sources: Kenya Ministry of Health, Department of Health Migori County, Department of Agriculture Migori County, et al. *Recipes for Complementary Feeding Children 6 to 23 Months of Age in Kisumu and Migori Counties*. Maternal and Child Survival Program, United States Agency for International Development; 2018. United States Department of Agriculture, Food and Nutrition Service. *Feeding Infants in the Child and Adult Care Food Program*. United States Department of Agriculture; 2021. Solid Starts. First foods database. Accessed August 20, 2022. https://solidstarts.com

Strategies for Nutritional Counseling

The goal of nutrition counseling is to help the patient initiate and maintain dietary changes. The nutritionist, the parent/caregiver, and the child/adolescent work together to assess current eating patterns and identify areas where change is needed. Making dietary changes is a gradual process. Goals should focus on the behaviors needed to achieve the desired dietary change. Self-monitoring, realistic expectations, and continued follow-up can help a patient maintain dietary changes. It is beneficial to involve the family, especially if they share responsibility for food selection and preparation. Having the support and understanding of family and friends makes success more likely. In making dietary changes, each individual's situation and background must be carefully considered, including the individual's ethnic background, religion, and SES.

An approach to nutrition counseling that can promote an empathetic relationship between the clinician and patient and help the patient or parent/caregiver explore their own motivations for change is motivational interviewing. There are three fundamental principles that must be present during motivational interviewing:

- **Expressing Empathy:** This involves sensing the hurt or pleasure of another as they have experienced it, as well as being able to hear and feel what a person is *not* saying or what they really mean.
- **Amplifying Ambivalence:** This involves exploring the person's internal thoughts and feelings about what is important to them (in particular, their values and self-imaging), highlighting their present behaviors that do not match up with those values, and illuminating the two conflicting agendas occurring internally. This allows the dietitian to guide the patient or parent toward resolving

the ambivalence they feel about the object of change or the conflict they are experiencing to a degree that is sufficient to move them toward change.

- **Supporting Self-Efficacy:** This involves encouraging the patient's own beliefs about their ability to control their own behavior and other events that affect their life, eliciting choices about how to accomplish their goals according to their values, and reinforcing confidence in their ability to realize those goals.

This form of counseling is a collaborative, goal-oriented style of communication with particular attention being given to the language of change. It is designed to strengthen personal motivation and commitment to a specific goal by eliciting and exploring the person's own reasons for change within an atmosphere of acceptance and compassion. Motivational interviewing focuses on working with patients in a way that is collaborative rather than prescriptive and honors the patient's autonomy and self-direction. This involves exploring the patient's capacity and having a genuine interest in the patient's experience and perspective.

Enteral Tube Feeding

Enteral tube feeding should be initiated when oral intake is inadequate to support growth or for nutritional repletion in a child with cancer. Patients eligible for tube feeding must have an intact GI tract. Enteral feedings have numerous advantages over PN, including maintenance of GI mucosal function, cost efficiency, and avoidance of PN complications such as bacterial infection, thrombosis, hepatic toxicity, cholestasis, and metabolic abnormalities. Tube feeding also offers the benefit of medication administration without oral ingestion. Despite these benefits, hesitation about the provision of tube feeding persists in the medical community; tube feeding is often presented as a punishment for not eating. Concerns also arise from patients (especially adolescents) and families due to the perceived inconvenience, discomfort, and poor body image associated with the placement of a NG tube. To optimize acceptance, tube feeding should be proposed as a positive intervention that is part of a comprehensive supportive care plan to aid in overall patient well-being. Support from the primary oncologist/pediatrician may assist in assuring the caregiver that this is an optimal clinical intervention for the child.

Types of Feeding Tubes

The type of feeding tube used depends on whether the tube is needed for a short- or long-term basis. There is a lack of consensus regarding the definition of *short-term* versus *long-term* enteral feeding; however, clinical practice guidelines suggest considering long-term tubes if the need for enteral access is expected to exceed two to four weeks.[41,42] In practice, the choice of feeding tube depends on availability, and short-term tubes are often used for longer than four weeks.

Short-term tubes include NG, nasoduodenal (ND), and NJ tubes, which go through the nose and end either in the stomach or small bowel. Short-term tubes may be indicated if a patient is unable to eat or drink for five days or more after an operation or treatment (e.g., bone marrow transplant) due to NV, loss of appetite, or a sore mouth/throat.

Tubes intended for long-term use include percutaneous endoscopic gastrostomy (PEG) tubes, also called gastrostomy tubes (G-tubes), and gastro-jejunostomy tubes (GJ-tubes). A child may benefit from a long-term feeding method in cases where the patient is severely malnourished and unable to meet nutrition needs via oral intake. Additional indications for the placement of a PEG/G-tube include

significant dysphagia and/or risk of aspiration, intractable vomiting, esophageal strictures, cancer of the head and neck, or radiation to the head, neck, or chest. The placement of a PEG/G-tube needs to be coordinated with the timing of chemotherapy or radiation in order to avoid an endoscopic or surgical procedure during periods of severe immunosuppression. Infection at the local insertion site can occur, so careful hygiene is required. Adequate GI function is also necessary for PEG/G-tube insertion.

The placement of long-term tubes requires access to advanced levels of care—including the availability of tubes and an experienced surgeon, interventional radiologist, or endoscopist—which are not always available or may pose a significant financial challenge for the patient's family. Adequate staffing is necessary for ongoing monitoring of all G-tubes, which should only be placed in institutions with the specified resources. A guide to determining your institution's capacity to undertake advanced forms of nutritional intervention has previously been published by the International Society of Pediatric Oncology.[43] Depending on the clinical needs of the child, the resources available, and the expertise of the medical staff, an appropriate tube can be selected (Figure 1).

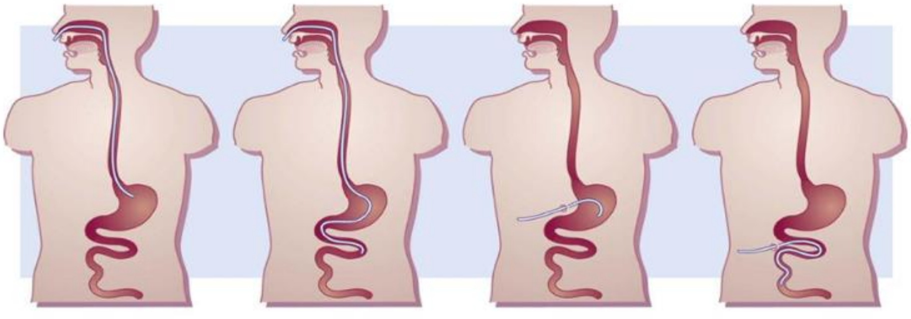

Figure 1. Enteral feeding tubes

The following provides a summary of the types of feeding tubes:

- **NG tubes:** NG tubes enter through the nose and feed into the stomach. These types of tubes are ideal for children who need tube feedings for only a short period of time (i.e., 1–3 months); however, a longer duration is often indicated. They can be placed and removed without surgery. Unfortunately, they can also be easily pulled out by the child.
- **ND tubes:** Similar to NG tubes, ND tubes enter through the nose, but extend into the beginning of the small intestine, the duodenum. These tubes are ideal for children who need tube feedings for a short period of time (i.e., 1–3 months) but are unable to tolerate feedings into the stomach. They can also be placed and removed without surgery but typically need to be placed in a hospital to ensure the tube extends into the duodenum.
- **NJ tubes:** NJ tubes are similar to ND tubes except that they extend a little bit further into the small intestine. Feedings go into the jejunum. Like other nasal tubes, NJ tubes are intended for short-term use (i.e., 1–3 months). They are specifically designed for patients who are unable to tolerate feedings into the stomach. They can be placed and removed without surgery, but they typically need to be placed in a hospital or clinic to ensure that the tube extends into the jejunum.
- **G-tube or PEG tube:** G-tubes are placed surgically or endoscopically directly through the skin and into the stomach. Children who require tube feeding for more than three months are likely to receive a G-tube. A G-tube may consist of a long tube, sometimes called a PEG tube.

- **GJ-tube/jejunostomy:** GJ-tubes are very similar to G-tubes in that they enter the stomach directly through the skin using the same site or stoma as a G-tube; they, too, enter the jejunum directly. Most have two feeding ports: one into the stomach and a second that extends into the small intestine. The longer internal tube allows feeding directly into the intestine for children who cannot tolerate gastric feeds. GJ-tubes are rarely a first tube. In most cases, a G-tube is converted to a GJ-tube when gastric feedings are not tolerated. These tubes are replaced at the hospital.

Placement of the Nasogastric Tube

The size of the tube used is determined by the weight of the child; however, substitutions may be made in settings with a limited variety of NG tubes. For pediatric patients, the most commonly used NG tube sizes are 6 to 12 French (1 F = 0.33 mm), depending on age and weight. The placement of the NG tube during routine sedation for other medical interventions may be ideal for insertion, but sedation is not necessary. Strategies that aim to “stiffen” silicone tubes make it easier to guide placement, while having patients swallow or drink water during placement will assist with correct localization and reduce discomfort.

The timing of tube replacement is dependent on the material of the tube. With the proper cleaning of the tube and good hygiene, silicone and polyurethane tubes can be kept in place for up to eight weeks[44] and are preferred over polyvinyl chloride (i.e., plastic) tubes, which need to be changed every five to seven days. In practice, silicone or polyurethane tubes have been kept in place for three to six months; however, it should be noted that there is a risk of bacterial contamination when using enteral feeding tubes and feeding systems, though this most likely results from contamination when handling or preparing the feeds and from the GI tract flora itself.[45,46]

Prior to administration of any liquid through an NG tube, verification of correct gastric placement is **essential**. A variety of methods are available to ensure proper placement at the time of insertion and on a regular basis while the NG tube is in place.[47,48] The strengths and weaknesses of each method are described in Table 6; often, a combination of approaches is the optimal method for ensuring proper placement. Placement of an NG tube is performed by a nurse or physician in most countries. Special care should be taken in children receiving proton pump inhibitors, as their use raises the pH, thereby preventing the detection of the appropriate placement of the NG tube.

Table 6. Advantages and disadvantages of methods of assessment for NG tube placement

Assessment method	Advantages	Disadvantages
X-ray visualization of tube	Most reliable method currently available	Costly, undesirable patient exposure to repeated X-rays, not always practical, repeated X-ray needed to determine dislodging of the NG tube
pH testing of aspirate	Easy to use, reliable, can be used to distinguish between gastric and intestinal tube placement	Continuous gastric feeding and medications for ulcers and reflux raise gastric pH, possibility of falsely indicating that the tube is not in the stomach
Visual assessment of aspirate	Useful in distinguishing between gastric and intestinal tube placement	Color and consistency of aspirate varies, of little value in differentiating between gastric and respiratory placement

Visualization of external tube length	Easy to use, may indicate if tube placement has shifted	Does not indicate location of tube, should never be used as a sole means of determining placement
Auscultation of air insufflated through the feeding tube for whooshing sounds	None	Highly unreliable, whooshing sound can be heard if tube is incorrectly placed in the lung

Abbreviation: NG, nasogastric.
Source: Simons SR, Abdallah LM. Bedside assessment of enteral tube placement: aligning practice with evidence. *Am J Nurs*. 2012;112(2):40-46.

Cleaning the Nasogastric Tube

It is important to ensure the NG tube stays clean to prevent blockage. Water flushes have been shown to be the most effective method in preventing clogs.[49] Before any feeds or formula are administered, flush the tube with 3-5 mL of water that is either **filtered** or **has been boiled and then cooled** to room temperature. Flush again with 3 to 5 mL of water post-feed. If any medication is given through the NG tube, it is important to always flush afterwards with 3 to 5 mL of water.[49]

If an NG tube is blocked, it recommended to use lukewarm water (filtered or boiled and then cooled to room temperature) to unblock the tube.[50] Use a 60 mL syringe with a catheter tip to ensure an easy flow of fluid, and a cloth to prevent any liquid from getting onto the patient. Draw up at least 15 mL of warm water into the syringe and push the water into the tube with the 60 mL syringe. Gently push and pull the plunger to loosen the clog. If the clog does not release right away, clamp the tube and let the water soak for 15 minutes to try to soften the blockage. Try gently massaging the tubing with your clean fingertips. Unclamp the tube and repeat the steps.

If the method described above is not successful, 1/8 of a teaspoon of baking soda and 5 mL of water can be mixed and allowed to soak in the tube.[50] Flush with 30 to 60 mL of water. The utilization of acidic beverages such as Coca-Cola® or cranberry juice may precipitate the caseinate in the feeding formula and contribute to clogging; thus, they should not be used.[49]

Determining the Formula

The choice of formula will depend on the clinical condition of the patient and institutional availability. Considerations in determining the type of formula, the volume of feeds, and the feeding schedule should include the patient's oral intake, sleep patterns, and lifestyle (e.g., school attendance), food allergies/intolerances, and GI conditions that affect dietary intake. In most children with cancer, a standard milk-based formula with or without fiber may be used to initiate tube feedings. Unflavored formulas have a lower osmolarity than do flavored products, are better tolerated, and are preferred for tube feeding. In patients with lactose intolerance, a soy-based, coconut-based, or lactose-free formula may be administered. Peptide and elemental formulas (i.e., formulas that contain proteins that are partially broken down [peptide] or completely broken down [elemental]) are ideal for patients with GI inflammation or malabsorption. Modification of the selected formula may be necessary in patients with underlying GI problems if there is an intolerance to the current formula, persistent constipation or diarrhea, or stomach pain. Appendix Table 13 provides an overview of commonly used industrialized formulas.

Homemade, blenderized, and strained feeds are a cost-effective way to provide EN; however, formulas should be standardized to ensure the delivery of adequate calories, protein, and micronutrients, and to ensure that the formulas are prepared according to institutional food-safety guidelines.[51] Cleaning the

NG tube will reduce the risk of blockages in the tube as well as the risk of an infection (refer to Cleaning the NG Tube).

Dosing, Rate, and Duration

Continuous feeding schedules are generally better tolerated than intermittent bolus feedings and are typically tolerated better in patients experiencing NV, constipation, or diarrhea. However, continuous feeds are highly disruptive to daytime routines and require constant oversight. Bolus feeds are preferred for active patients in the day and in patients with limited oversight of the administration of feeds; however, it should be noted that bolus feeds are contraindicated in patients with tubes extending into the small intestine. Nighttime continuous feeds may complement daytime bolus feeds, yet nighttime feeds also require oversight, which may be a limiting factor in their administration.

Continuous tube feeds are initiated at 1 to 2 mL/kg/hour and increased by 1 to 2 mL/kg/hour, as tolerated, until the goal rate is achieved (refer to Table 7). Elevating the head of the bed to > 30° during and after tube feeding may assist in reducing high gastric residuals caused by delayed gastric emptying and, accordingly, promote digestion. The use of prokinetic medications may be considered; however, clinical response is variable, and evidence for efficacy, especially in pediatric oncology settings, is limited.

Table 7. Initiating and advancing enteral nutrition*

Type	Age	Initiation	Advancement	Suggested tolerance volume
Continuous	0–12 months	1–2 mL/kg/hour	1–2 mL/kg every 2–8 hours	6 mL/kg/hour
	1–6 years	1 mL/kg/hour	1 mL/kg every 2–8 hours	4–5 mL/kg/hour
	≥ 7 years	15–40 mL/hour	15–40 mL every 2–8 hours	100–150 mL/hour
Bolus	0–12 months	10–60 mL every 2–3 hours	10–60 mL per feeding	90–180 mL every 4–5 hours
	1–6 years	30–90 mL every 2–3 hours	30–90 mL per feeding	100–300 mL every 4–5 hours
	≥7 years	60–120 mL every 2–3 hours	60–90 mL per feeding	240–480 mL every 4–5 hours

Abbreviations: mL, milliliter; kg, kilogram.
*The suggested rates of initiation and advancement are intended to guide clinical decision making. The actual rate of initiation and advancement will depend on factors such as current side effects (e.g., diarrhea, NV), the size of the child, and pre-existing conditions (e.g., SAM/MAM).

In the absence of diet pumps, gravity feeds or bolus feeds with a sterile syringe are an inexpensive and easy way to deliver EN via either NG or G-tubes.[51] Gravity feeds should be administered with the head-end of the bed elevated and as slowly as possible, using the feeding bag tubing clamp to control speed while always monitoring the child's ability to tolerate the feed (refer to Figure 2).

Re-evaluation of nutritional status and feeding methods is indicated if feeding problems persist and growth is not observed. Refer to Table 8 for a summary.

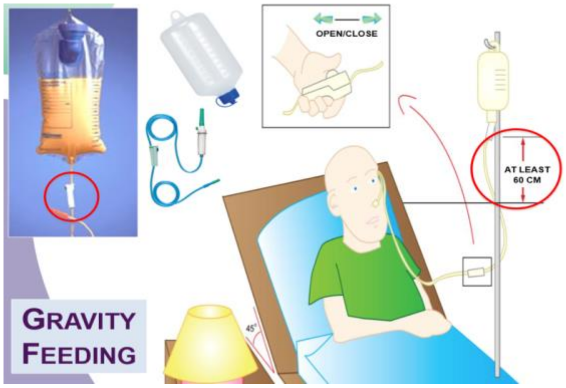

Figure 2. Illustration of gravity feeds

Table 8. Potential complications with enteral feeds

GI		
Complication	Possible cause	Management/prevention

Aspiration pneumonia	• Aspiration of feedings<br>• Emesis<br>• Displacement or migration<br>• Supine position during feeds<br>• Gastroesophageal reflux<br>• Presence of NG tube preventing complete closure of esophagus<br>• Delayed gastric emptying	• Confirm tube placement prior to administration of feeds<br>• Elevate head 30 to 45 degrees<br>• Ensure tube placement into the duodenum<br>• Consider prokinetics<br>• Change the formula
Bloating, cramps, or gas	• Air in tubing	• Remove as much air as possible when setting up feeding
Diarrhea	• Bacterial contamination of formula<br>• Food allergies<br>• Hyperosmolar formulas<br>• Too-rapid infusion<br>• Low fiber intake<br>• Fat malabsorption<br>• Medications (e.g., antibiotics, antacids, sorbitol, magnesium, or antineoplastic agents)	• Properly store, prepare, and administer feeds<br>• Change feeding bag daily<br>• Limit hang time of formulas to 8 to 12 hours for commercially manufactured products<br>• Utilize undiluted, ready-to-feed products to minimize risk<br>• Consider changing the formula<br>• Consider an isotonic formula<br>• Employ a slow rate of infusion<br>• Consider a fiber-containing formula<br>• Consider changing the formula to a product with partial MCT content
Dumping syndrome	• High carbohydrates content<br>• Rapid feeding rate<br>• Cold formula	• Use formulas with lower carbohydrate content or complex carbs<br>• Reduce feeding rate and volume per session<br>• Prefer continuous over bolus feeding<br>• Administer formula at room temperature
Vomiting	• Rapid feeding<br>• Hyperosmolar formulas<br>• Delayed gastric emptying<br>• Obstruction<br>• Too-rapid advancement of volume or concentration	• Employ a slow rate of feeding<br>• Consider changing to an isotonic formula<br>• Consider a transpyloric route for feeding<br>• Consider continuous infusion<br>• Elevate the head of the bed 45 degrees during feeding<br>• Check residuals prior to feeding<br>• Consider prokinetics<br>• Discontinue feeding<br>• Return to previously tolerated strength and volume, and advance more slowly
**Mechanical**		
Complication	Possible cause	Management/Prevention
Clogged tube	• Inadequate flushing<br>• Inadequate crushing of medications<br>• Formula or medication residue<br>• Kinking of the feeding tube<br>• Highly viscous, fiber-rich formulas	• Flush tube before and after gravity or bolus feedings, and every four to eight hours during continuous feeds<br>• Dissolve crushed tablets in warm water<br>• Flush tube before and after medication administration; avoid mixing formula with medication<br>• Replace feeding tube
Tube displacement	• Coughing<br>• Vomiting<br>• Inadvertent dislodgement<br>• Removal of tube by patient	• Replace feeding tube
**Metabolic**		
Complication	Possible cause	Management/Prevention

Dehydration	• Inadequate free water<br>• Hyperosmolar feedings	• Monitor input and output<br>• Monitor hydration status of patient routinely<br>• Assess renal solute load of formula
Overhydration	• Excessive fluid administration<br>• Too-rapid refeeding of patients with MAM/SAM	• Advance feeds slowly<br>• Allow a five- to seven-day period to meet nutritional goals
Electrolyte imbalance	• Formula components<br>• Medical condition/diagnosis	• Evaluate electrolyte adequacy of the specific formula and appropriateness of the formula dilution<br>• Monitor electrolytes, phosphorus, BUN, creatinine, and glucose
Failure to achieve weight gain	• Inadequate nutrient intake	• Evaluate adequacy of nutrient intake<br>• Perform routine nutritional assessments

Abbreviations: GI, gastrointestinal; NG, nasogastric; MCT, medium chain triglyceride; MAM, moderate acute malnutrition; SAM, severe acute malnutrition; BUN, blood urea nitrogen.

Criteria for Monitoring Nutritional Intervention

The purpose of nutrition monitoring and evaluation is to determine the amount of progress made and whether the nutrition goals are being met. During the initial nutrition assessment, appropriate outcomes/indicators are selected to be monitored and evaluated during the next re-assessment. There are several factors to consider when selecting indicators, which include the medical diagnosis, patient goals, practice setting, and disease state and/or severity. The following chart (Table 9) lists the factors to consider when evaluating the efficacy of nutritional interventions.

Table 9. Considerations in nutritional interventions

Food and nutrition	Anthropometric measurement	Biochemical data	Nutrition-focused physical examination
• Food and nutrient intake<br>• Food and nutrient administration<br>• Knowledge and beliefs<br>• Food supply availability	• Height<br>• Weight<br>• Body mass index<br>• Growth patterns<br>• Percentile ranks<br>• Weight history	• Laboratory data (e.g., electrolytes, minerals, and vitamins)	• Physical appearance<br>• Muscle and fat wasting<br>• Fluid accumulation/ edema

Growth Velocity

Evaluation of growth velocity is valuable in assessing the growth of children, particularly those experiencing a life-threatening diagnosis such as cancer. Length and height measurements help to detect growth abnormalities in length/height, and weight should be monitored for acute or chronic nutritional deficits. Oral intake must be monitored to ensure that nutritional requirements are being met, which requires regular anthropometric measurements. In Table 10, the desired increase in weight and length/height per month are provided. For example, a 20-month-old child should gain 4 to 10 g of weight per day (equal to 28–70 g per week) and 0.7 to 1.1 cm in length per month.

Table 10. Growth velocity

Age	Weight (g/day)	Length (cm/month)
< 3 months	25–35	2.6–3.5
3–6 months	15–21	1.6–2.5
6–12 months	10–13	1.2–1.7
1–3 years	4–10	0.7–1.1
4–6 years	5–8	0.5–0.8
7–10 years	5–12	0.4–0.6

Abbreviations: g, gram; cm, centimeter.
Source: Fomon SJ, Haschke F, Ziegler EE, Nelson SE. Body composition of reference children from birth to age 10 years. *Am J Clin Nutr.* 1982;35(5 Suppl):1169-1175.

Catch-up growth is needed for patients with SAM and other severe nutrition concerns that have impacted growth in weight or height. Catch-up growth formulas have been developed and are used to determine the patient's nutritional requirements; to do so, the RDA for the age corresponding to the patient's ideal weight (50th percentile or 0 Z-score for weight/height) is multiplied by the the ratio of the ideal weight to the patient's actual weight. Alternatively, the Food and Agriculture Organization of the United Nations has recommended the following increase in energy requirements to allow for twice the normal growth rate[52]:

- **6–9 months:** 14.5% increase over energy requirement
- **9–12 months:** 8.5% increase over energy requirement
- **12–18 months:** 5% increase over energy requirement
- **18–24 months:** 3.5% increase over energy requirement

An increase in caloric intake by 20% to 40% above the estimated calorie needs has also been suggested to achieve catch-up growth.[53]

Specialized Diets

Food Safety Diet

Treatment for cancer often results in neutropenia (i.e., low WBC count), which increases the risk of developing infection. The duration and severity of neutropenia can be predicted by the dose and class of the chemotherapy agent, the dose and field of the radiation administered, and the cancer. Food is an established vector of infections, and it is presumed that neutropenia increases the risk of a food-borne illness, especially in the setting of severe neutropenia (i.e., an absolute neutrophil count < 500 cells/mm^3). The neutropenic diet or low-microbial diet are frequently prescribed to minimize the risk of an infection through bacterial translocation by eliminating foods susceptible to infectious organisms.

Historically, all uncooked fruits and vegetables without peels, unpasteurized juice and dairy products, cheeses with molds, salad bars, dried fruits, delicatessens, raw fish, and certain spices were avoided as part of a neutropenic diet. However, as the evidence supporting these restrictions is limited, the recommendations have been revised.[54] At the very minimum, food safety guidelines should always be followed by any patient undergoing anticancer therapy. These guidelines will minimize the risk of food contamination. For patients with neutropenia or undergoing stem cell transplant, it is imperative to provide the family with a list of local, acceptable, and safe foods and to educate families to ensure that food is not bought from street vendors or received from sources where the safety guidelines followed are not known. Patients without access to clean water must avoid foods that require cleaning prior to

consumption, or they must boil the water prior to cleaning fruits and vegetables for consumption. Visual education tools have been effective in providing education to Spanish-speaking patients.[55,56]

Low-Sodium Diet

Certain chemotherapy drugs cause edema/fluid retention in the body. Water retention occurs when excess fluids build up inside the body, often in the circulatory system or within tissues and cavities. This leads to swelling in the hands, feet, ankles, and legs, and may affect other parts of the body, such as the abdomen. Fluid retention may also occur due to heart failure, liver disease, or kidney failure. Each of these conditions may lead to unexplained rapid weight gain.

A diet low in sodium may help to reduce fluid retention. Sodium controls the fluid balance in the body and maintains the blood volume and blood pressure. Eating too much sodium may raise the blood pressure, leading to fluid retention and swelling. Strategies to reduce sodium intake are provided in Table 11.

Table 11. Tips to reduce sodium intake

- Reduce the amount of salt used in cooking. Sea salt is no better than regular salt.
- Select low-sodium foods. Many salt-free or reduced-salt products are available. When reading food labels, *low sodium* is defined as < 140 mg of sodium per serving.
- Be creative and season your foods with spices, herbs, lemon, garlic, ginger, vinegar, and pepper. Sodium-free seasonings may be available.
- Remove the saltshaker from the table.
- Read ingredient labels to identify foods high in sodium. Items with ≥ 400 mg of sodium per serving are considered high in sodium. High-sodium food additives include salt, brine, or other items that contain sodium, such as monosodium glutamate.
- Eat more home-cooked meals so that you can control the amount of salt added.
- Avoid smoked, cured, salted, or canned meat, fish, or poultry, including bacon, cold cuts, ham, frankfurters, sausages, sardines, caviar, and anchovies.
- Avoid canned foods, such as vegetables, beans, meat, and chili.
- Avoid regular and processed cheeses; look for low-sodium cheeses instead.
- Avoid regular canned and dehydrated soup, broth, cup noodles, and seasoned ramen mixes.
- Avoid soy sauce, seasoning salt, and other sauces and marinades.

Abbreviations: mg, milligram.

Low-Fat Diet

The administration of asparaginase and steroid medications can cause significant changes in serum lipid levels, which may lead to hypertriglyceridemia. A low-fat or fat-restricted diet may aid in the management of elevated triglyceride levels. The low-fat diet focuses on limiting the total amount of fat in the diet as well as limiting saturated and trans fats. Food high in saturated fats include marbled (i.e., fatty) meat, poultry skin, bacon, sausage, whole milk, cream, and butter. Trans fats are found in stick margarine, some fried foods, and baked goods such as donuts, pizza, pie crusts, biscuits, and cookies. Recommended substitutions for high-fat foods are found in Table 12.

Table 12. Substitutes for high-fat foods

High-fat foods to avoid	Recommended substitutions
Butter	Light or diet margarine (without trans fats), spray margarine, olive or canola oil, applesauce for baking
Regular cheese	Low-fat or fat-free cheese, cottage cheese, mozzarella cheese, Requesón, quesillo
Whole milk	1% or fat-free (skim) milk

Cream cheese	Low-fat or fat-free cream cheese
Regular ice cream	Fat-free or low-fat frozen yogurt, sorbet, gelato, frozen fruit blended with skim or almond milk
Creamy salad dressings	Oil and vinegar or light salad dressings, freshly squeezed lemon
Mayonnaise	Light mayonnaise or non-fat Greek yogurt, hummus
Meat	Select choice or select grades of beef instead of prime grades of beef; tenderloin with fat trimmed instead of spareribs; ground sirloin / ground round (90–95% lean) instead of regular ground beef
Chicken with skin	Baked or broiled chicken breasts without skin
Whole egg	Select egg whites
Donuts, pizza, pie crusts, biscuits, and cookies	Cauliflower pizza made with low-fat mozzarella cheese, angel food cake, two-ingredient cookies (i.e., mashed banana and quick oats)
Processed meats: salami, Russians, sausages, frankfurters, ham, corned beef, meat pies, savory tarts	Roasted chicken or turkey, hummus and veggie wrap, black bean wrap, baked chicken or veggie empanada

Parenteral Nutrition (PN)

PN is indicated when EN is inaccessible or clinically contraindicated. Generally, when the period of no or minimal oral or enteral intake is anticipated to be longer than five to seven days, most children will benefit from PN. However, PN is not intended for routine use because it increases the risk of infections, occlusion, central venous thrombosis, pulmonary embolism, hyperglycemia, hypertriglyceridemia, and hepatobiliary complications.[51,57] If possible, the maintenance of gut integrity with very small amounts of oral feeding is beneficial during PN. If the administration of PN is indicated by a nutritional assessment, consult with the treating physician to determine if the need is expected to be prolonged and if access to PN is available.

Prior to initiating PN, consider the following[51,57]:

1. **Access:** Peripheral PN can only be administered for up to 2 weeks, and the dextrose concentration cannot exceed 12.5%. Central access allows for a longer duration and a dextrose concentration of up to 20%.
2. **Type of PN solutions available:** In the absence of a standard or individualized PN solution, an amino acid intravenous solution alone or combined with electrolytes and/or glucose may be used.
3. **Monitoring:** Monitoring glucose levels, electrolytes (i.e., sodium, potassium, phosphorus, magnesium, chloride, and calcium), and hepatic function tests (e.g., AST, ALT, total and direct bilirubin, and, if indicated, ultrasound to confirm liver enlargement) are essential to ensuring safe and effective nutritional support. Ideally, glucose and electrolyte levels should be monitored daily until stabilization. Once the patient is metabolically stable, glucose and electrolyte levels can be monitored once every one to two weeks.[58] Hepatic function tests should be performed as needed during the stabilization phase and once a month following stabilization.

The following tables can be utilized to guide the initiation, advancement, and goals for macronutrients (Table 13) and to provide general dosing guidelines for micronutrients in PN solutions (Tables 14–16). The European Society for Paediatric Gastroenterology Hepatology and Nutrition's Pediatric Parenteral Nutrition Tool may also be utilized to assist in PN planning: https://espghan.info/paediatric-parenteral-nutrition-tool/index.php.[59]

Table 13. Dosing for initiation and advancement of PN macronutrients

	Initiation		Advance by		Goals	
Infants (< 1 year)	Preterm	Term	Preterm	Term	Preterm	Term
Protein (g/kg/day)*	1–3 (max. 3–4)	2.5–3	—	—	3–4	2.5–3
Dextrose (mg/kg/min)	6–8	6–8	1–2	1–2	10–14 (max. 14–18)	10–14 (max. 14–18)
ILE (g/kg/day)**	0.5–1	0.5–1	0.5–1	0.5–1	3 (max. 0.15)	2.5–3 (max. 0.15)
Children (1–10 y)						
Protein (g/kg/day)	1.5–2.5		—		1.5–2.5	
Dextrose (mg/kg/min)	3–6		1–2		8–10	
ILE (g/kg/day)**	1–2		0.5–1		2–2.5	
Adolescents						
Protein (g/kg/day)	0.8–2		—		0.8–2	
Dextrose (mg/kg/min)	2.5–3		1–2		5–6	
ILE (g/kg/day)**	1		1		1–2	

Abbreviations: PN, parenteral nutrition; ILE, lipid injectable emulsion; g, grams; mg, milligram; kg, kilogram; min, minute; max, maximum; y, year.
*Protein does not need to be titrated; protein needs are increased with critical illness.
**ILE dosing is based on soybean oil-based emulsion. See manufacturer's product information for dosing of other ILE products.
Note: Glucose infusion rate (GIR) calculation (mg/kg/m) = [dextrose (g/day) × 1000] / [24 (hours/day) × 60 (min/hour) × weight (kg)]
Source: American Society for Parenteral and Enteral Nutrition. *Appropriate dosing for parenteral nutrition: ASPEN recommendations.* American Society for Parenteral and Enteral Nutrition; 2019:1-3.

Table 14. PN electrolyte and mineral daily dosing*

	Preterm neonates	Infants/children	Children/adolescents (> 50 kg)
Sodium	2–5 mEq/kg	2–5 mEq/kg	1–2 mEq/kg
Potassium	2–4 mEq/kg	2–4 mEq/kg	1–2 mEq/kg
Calcium	2–4 mEq/kg	0.5–4 mEq/kg	10–20 mEq
Phosphorus	1–2 mmol/kg	0.5–2 mmol/kg	10–40 mmol
Magnesium	0.3–0.5 mEq/kg	0.3–0.5 mEq/kg	10–30 mEq
Acetate	As needed to maintain acid base-balance		
Chloride	As needed to maintain acid base-balance		

Abbreviations: PN, parenteral nutrition; kg, kilogram; mEq, milliequivalents; mmol, millimole.
*Use caution in relation to compatibility when prescribing calcium and phosphorus.
Source: American Society for Parenteral and Enteral Nutrition. *Appropriate dosing for parenteral nutrition: ASPEN recommendations.* American Society for Parenteral and Enteral Nutrition; 2019:1-3.

Table 15. PN daily multiple vitamin product dosing

Weight (kg)	Dose (mL)
< 2.5	2 mL/kg
≥ 2.5	5 mL

Abbreviations: PN, parenteral nutrition; kg, kilogram; mL, milliliter.
Source: American Society for Parenteral and Enteral Nutrition.
Appropriate dosing for parenteral nutrition: ASPEN recommendations.
American Society for Parenteral and Enteral Nutrition; 2019:1-3.

Table 16. PN trace element daily dosing*

Trace element	Preterm neonates	Term neonates (3–10 kg)	Children (10–40 kg)	Adolescents (> 40 kg)
Zinc		250 mcg/kg	50 mcg/kg (max. 5000 mcg/day)	2–5 mg
Copper	20 mcg/kg	20 mcg/kg	20 mcg/kg (max. 500 mcg/day)	200–500 mcg
Manganese	1 mcg/kg	1 mcg/kg	1 mcg/kg (max. 55 mcg/day)	40–100 mcg
Chromium	0.05–0.3 mcg/kg	0.2 mcg/kg	0.2 mcg/kg (max. 5 mcg/day)	5–15 mcg
Selenium	2 mcg/kg	2 mcg/kg	2 mcg/kg (max. 100 mcg/day)	40–60 mcg

Abbreviations: PN, parenteral nutrition; mcg, microgram; kg, kilogram; mg, milligram.
*Note: These requirements are different than the multi-trace element products currently available in the United States.
Source: American Society for Parenteral and Enteral Nutrition. *Appropriate dosing for parenteral nutrition: ASPEN recommendations.* American Society for Parenteral and Enteral Nutrition; 2019:1-3.

Severe Acute Malnutrition Management in the Pediatric Oncology Setting

The following section provides a brief overview of the general principles, set forth by the WHO, of treating SAM. However, the WHO does not address the coexistence of SAM and childhood cancer. The modifications provided below are based on clinical experience and not based on well-designed clinical trials; research in this area is urgently needed. For the WHO guidelines, a comprehensive clinical reference is available in the original[37] and updated version.[30] Generally speaking, these guidelines may be implemented into pediatric cancer care. The management of SAM may be broken down into 3 phases: (1) initial treatment/stabilization (2–7 days), (2) rehabilitation (several weeks to months), and (3) follow-up. It is important to recognize that it is common for children and adolescents with cancer to present with SAM but not with the typical signs and symptoms associated with SAM. As a result, adhering to the WHO guidelines may not always be indicated for a child with SAM and cancer. It is important to screen patients according to the WHO guidelines and then create a nutrition rehabilitation plan with the oncology team based on the observed signs and symptoms, treatment plan, and availability/feasibility of nutrition interventions. In assessing a child with newly diagnosed SAM, the dietitian should screen to determine whether it is likely due to low SES or is related to the cancer itself. It may be particularly hard to decipher between these two issues in children who present with advanced-stage disease. If SES is likely to the be the cause, working with non-governmental organizations and volunteer groups to provide education on food security will aid in remediation and the prevention of recurrence.

Guidelines for the management of SAM are broken down into 10 steps, based on the signs and symptoms present: (1) treat/prevent hypoglycemia, (2) treat/prevent hypothermia, (3) treat/prevent infection, (4) correct electrolyte imbalance, (5) treat/prevent dehydration, (6) correct micronutrient deficiencies, (7) begin cautious feedings, (8) strive for catch-up growth, (9) provide sensory stimulation and emotional support, and (10) establish a follow-up plan. In most settings, steps 1 through 5 are managed by the physician. The dietitian should work alongside the physician to provide dietetic support as needed. Each of these signs and symptoms may also be altered due to the cancer itself and not necessarily related to SAM. Once immediate medical treatment is provided, a formula diet should be implemented and complemented with breastfeeding (when age appropriate). Due to problems with infections, impaired liver and intestinal function, and electrolyte balance, special formulas that are modified in protein, fat, and sodium (e.g., WHO F-75 and F-100) are provided during the initial and rehabilitation phase. A description of the therapeutic formulas used for SAM management is provided in the Appendix Table 13.

Initial Treatment/Stabilization

In the stabilization phase, formula (i.e., F-75) should be administered every two to four hours throughout the day and evening. If this is not tolerated, the interval between feeds should be reduced along with volume or the interval between feeds should be increased. It is also crucial to recognize that if metabolic abnormalities and other associated symptoms of SAM are attributed to the cancer itself rather than to malnutrition, initiation with F-100 is a reasonable initial intervention during the stabilizationion phase. In some instances, the use of RUTF may also be tolerated and safe. The selection of intervention will depend on the patient's clinical condition as well as institutional and individual access to formulas. Most importantly, the clinician needs to progress slowly to avoid any complications associated with feeding too much too soon.

During this stage, the target calorie and protein intake for children under 7 years of age is 80 to 100 kcal/kg and 1 to 1.5 g protein/kg.[37] The minimum calories per day to avoid additional muscle breakdown is 80 kcal/kg. The maximum that should be given during this period is 100 kcal/kg per day; if tolerated, strive to achieve 130 kcal/kg/day for children less than 5 years of age (only increase if the child is stable and improving). For older children, 75 kcal/kg (7–10 years of age), 60 kcal/kg (11–14 years of age), and 50 kcal/kg (15–18 years of age) are recommended.[37] For children less than or equal to six months of age, refer to the WHO SAM guidelines.[30]

Rehabilitation

When the child demonstrates an interest and willingness to eat by mouth, the initial or stabilization phase is complete, and the child enters the rehabilitation phase. In children with cancer, it is often the case that the rehabilitation phase is started immediately after the acute medical circumstances are addressed by the medical team; the stabilization phase is not always indicated and will depend on the clinical circumstances. In children with SAM, advancing to the rehabilitation phase usually takes between two and seven days. If the child was started on F-75, this should be replaced with an equal amount of F-100 for two days before increasing the volume. The evidence increasingly suggests that RUTF may be used in lieu of F-100.[30] This is particularly beneficial if supplies of F-100 are limited. During the rehabilitation phase, F-100 or RUTF should be administered every four hours throughout the day. F-100 should be continued until the child achieves −1 SD body mass index. Due to limited resources, it is also reasonable to administer other formulas; however, if possible, select formulations that are as low in osmolarity as possible. The transition between phases is completely dependent on the child's overall condition, ability to tolerate food, and cancer treatment initiation/side effects

During the rehabilitation phase, children less than 24 months of age should receive 150 to 220 kcal/kg/day[60] or about 1/3 higher than what was provided during the stabilization phase[61]; concern is warranted if intake is below 130 kcal/kg/day.[37] Children older than 24 months and up to 5 years of age should target at least 100 to 135 kcal/kg/day or about 1/3 higher than what was provided during the stabilization phase.[30] A similar approach may be applied to children older than five years of age, as no clear guidelines exist.

Monitoring

The child's weight is the optimal indicator of success of the intervention in a child with SAM, and weight gain should be greater than 5 g/kg of body weight per day.[37] Progress can be defined as weight gain of at least 10 to 15 g/kg per day.[37] If the patient does not have weight gain of at least 5 g/kg/day or is not

responding to treatment, the intervention is failing, and other interventions may be required. Table 10 (above) can serve as useful guide for monitoring normal growth and development.

However, in children with SAM who also have a large solid tumor burden, mid-upper arm circumference (MUAC) is the only reliable indicator of nutritional progress, as weight is often further reduced due to tumor debulking or to a rapid response to the treatment. Immediate changes in MUAC are typically not observed (e.g., in contrast to weight, daily fluctuations are not usually observed). Thus, monitoring the child clinically may be used as an indicator of the effectiveness of the intervention. This can include the child becoming more alert, able to sit or stand, or engaging in physical or verbal conversation.

Micronutrients

The correction of vitamin and mineral deficiencies is critical to ensuring optimal recovery. Low-dose vitamin A (5000 IU/day) should be administered daily from admission until discharge in patients who are receiving therapeutic foods that do not contain vitamin A. Low-dose supplementation is not necessary in patients receiving therapeutic foods that contain vitamin A. High-dose vitamin A should be administered on day 1, day 2, and day 15 in children who have clear signs of vitamin A deficiency or measles. The dosing for high-dose vitamin A is as follows: < 6 months: 50,000 IU; 6 months to 12 months: 100,000 IU; and > 12 months: 200,000 IU. This should be administered irrespective of whether vitamin A is in the therapeutic food being administered (e.g., F-75 and F-100 vs. homemade formula).

Children with SAM are likely to have several other micronutrient deficiencies. A daily supplementation regimen is recommended; however, for children receiving therapeutic foods fortified with micronutrients, supplementation is not warranted. The following may be used as a guide in children who are receiving formulas that are not fortified with multivitamins.

- **Multivitamins:** Twice the recommended intake daily; some multivitamins may include the individual nutrients below, and additional supplementation may not be warranted
- **Folic acid:** 1 mg/day (give 5 mg on day 1); monitoring of supplementation is required in patients receiving methotrexate
- **Zinc:** 10 mg/day < 6 months; 20 mg/day > 6 months for 10 to 14 days, if diarrhea is present
- **Copper:** 0.3 mg/kg/day
- **Iron:** 3 mg/kg/day in 2 divided doses; a maximum of 60 mg/day for 3 months only once stabilization has been achieved; administration of iron must be coordinated with oncologist/pediatrician to ensure no contraindication with therapy

Additional supplementation is **not warranted** if the patient is receiving RUTF, as vitamins and minerals are already included in the formulation. Consultation with the oncologist is necessary for patients receiving methotrexate (i.e., anti-folate chemotherapy drug) prior to initiating supplementation with folate. Folate-free multivitamins and RUTF are available.

Collaboration with Other Disciplines

To provide proper care and improve patient outcomes, nutritionists and dietitians must collaborate effectively with other disciplines. When all medical and healthcare professionals are working together, a more communicative environment develops. With increased collaboration, medical personnel are interacting on a professional level, sharing ideas about the patient's treatment, and working together to maintain continuity of care. As part of a multidisciplinary team consisting of pediatric oncologists, nursing staff, social workers, play therapists, volunteer groups, and others, dietitians should attend ward rounds to obtain knowledge about the patient's medical diagnosis and to give feedback on the patient's nutritional status. In this way, medical doctors, nurses, and other members of the medical team learn about the importance of nutrition. Volunteer groups spend time with the children and their families and can be of great help in providing essential items, based upon the family's needs.

References

1. National Cancer Institute. SEER Cancer Statistics Review 1975-2009. Updated August 20, 2012. Accessed August 20, 2022. http://seer.cancer.gov/csr/1975_2009_pops09/index.html

2. Pui CH, Relling MV, Campana D, Evans WE. Childhood acute lymphoblastic leukemia. *Rev Clin Exp Hematol*. 2002;6(2):161-180.

3. Jarrett RF, MacKenzie J. Epstein-Barr virus and other candidate viruses in the pathogenesis of Hodgkin's disease. *Semin Hematol*. 1999;36(3):260-269.

4. Weinstein JL, Katzenstein HM, Cohn SL. Advances in the diagnosis and treatment of neuroblastoma. *Oncologist*. 2003;8(3):278-292.

5. McDowell HP. Update on childhood rhabdomyosarcoma. *Arch Dis Child*. 2003;88(4):354-357.

6. Kline NE, Sevier N. Solid tumors in children. *J Pediatr Nurs*. 2003;18(2):96-102.

7. National Academy of Sciences, Institute of Medicine. *Dietary Reference Intakes for Energy, Carbohydrate, Fiber, Fat, Fatty Acids, Cholesterol, Protein, and Amino Acids (Macronutrients)*. Washington, DC: The National Academy Press; 2002.

8. McRorie JW, Jr., McKeown NM. Understanding the physics of functional fibers in the gastrointestinal tract: an evidence-based approach to resolving enduring misconceptions about insoluble and soluble fiber. *J Acad Nutr Diet*. 2017;117(2):251-264.

9. Rogers PC, Schoeman J. Nutritional assessment and intervention. In: Rodrigues-Galindo C, Stefan DC, eds. *Pediatric Hematology-Oncology in Countries with Limited Resources*. Springer; 2014:91-112.

10. McRorie JW, Jr. Evidence-based approach to fiber supplements and clinically meaningful health benefits, part 2: what to look for and how to recommend an effective fiber therapy. *Nutr Today*. 2015;50(2):90-97.

11. National Research Council. *Diet and Health: Implications for Reducing Chronic Disease Risk*. Washington, DC: The National Academies Press; 1989:768.

12. Jovanovic-Malinovska R, Kuzmanova S, Winkelhausen E. Oligosaccharide profile in fruits and vegetables as sources of prebiotics and functional foods. *Int J Food Prop*. 2014;17(5):949-965.

13. Khan WU, Sellen DW. *Zinc Supplementation in the Management of Diarrhoea*. Canada: World Health Organization; 2011.

14. Busch RA, Collier BR, Kaspar MB. When can we feed after a gastrointestinal bleed? *Curr Gastroenterol Rep*. 2022;24(1):18-25.

15. Dupuis LL, Sung L, Molassiotis A, Orsey AD, Tissing W, van de Wetering M. 2016 updated MASCC/ESMO consensus recommendations: prevention of acute chemotherapy-induced nausea and vomiting in children. *Support Care Cancer*. 2017;25(1):323-331.

16. Urbach DR, Rotstein OD. Typhlitis. *Can J Surg*. 1999;42(6):415-419.

17. Abu-El-Haija M, Kumar S, Szabo F, et al. Classification of acute pancreatitis in the pediatric population: clinical report from the NASPGHAN Pancreas Committee. *J Pediatr Gastroenterol Nutr*. 2017;64(6):984-990.

18. Abu-El-Haija M, Uc A, Werlin SL, et al. Nutritional considerations in pediatric pancreatitis: a position paper from the NASPGHAN Pancreas Committee and ESPGHAN Cystic Fibrosis/Pancreas Working Group. *J Pediatr Gastroenterol Nutr*. 2018;67(1):131-143.

19. Abu-El-Haija M, Wilhelm R, Heinzman C, et al. Early enteral nutrition in children with acute pancreatitis. *J Pediatr Gastroenterol Nutr*. 2016;62(3):453-456.

20. Shah AS, Wilson DP. Primary hypertriglyceridemia in children and adolescents. *J Clin Lipidol*. 2015;9(5 Suppl):S20-28.

21. Khwaja A. KDIGO clinical practice guidelines for acute kidney injury. *Nephron Clin Pract*. 2012;120(4):c179-c184.
22. Park PG, Hong CR, Kang E, et al. Acute kidney injury in pediatric cancer patients. *J Pediatr*. 2019;208:243-250.
23. Olowu WA, Adelusola KA. Pediatric acute renal failure in southwestern Nigeria. *Kidney Int*. 2004;66(4):1541-1548.
24. Sethi SK, Maxvold N, Bunchman T, Jha P, Kher V, Raina R. Nutritional management in the critically ill child with acute kidney injury: a review. *Pediatr Nephrol*. 2017;32(4):589-601.
25. Kyle UG, Akcan-Arikan A, Silva JC, Goldsworthy M, Shekerdemian LS, Coss-Bu JA. Protein feeding in pediatric acute kidney injury is not associated with a delay in renal recovery. *J Ren Nutr*. 2017;27(1):8-15.
26. Desloovere A, Renken-Terhaerdt J, Tuokkola J, et al. The dietary management of potassium in children with CKD stages 2-5 and on dialysis-clinical practice recommendations from the Pediatric Renal Nutrition Taskforce. *Pediatr Nephrol*. 2021;36(6):1331-1346.
27. McAlister L, Pugh P, Greenbaum L, et al. The dietary management of calcium and phosphate in children with CKD stages 2-5 and on dialysis-clinical practice recommendation from the Pediatric Renal Nutrition Taskforce. *Pediatr Nephrol*. 2020;35(3):501-518.
28. Sala A, Rossi E, Antillon F, et al. Nutritional status at diagnosis is related to clinical outcomes in children and adolescents with cancer: a perspective from Central America. *Eur J Cancer*. 2012;48(2):243-252.
29. Balint JP. Physical findings in nutritional deficiencies. *Pediatr Clin North Am*. 1998;45(1):245-260.
30. World Health Organization. *Guideline: Updates on the Management of Severe Acute Malnutrition in Infants and Children*. Geneva, Switzerland: World Health Organization; 2013.
31. Institute of Medicine. *Dietary Reference Intakes for Calcium and Vitamin D*. Washington, DC: National Academies Press; 2011.
32. Food and Nutrition Board, Institute of Medicine. Introduction to dietary reference intakes. In: Compounds IoMUPoDAaR, ed. *Dietary Reference Intakes for Vitamin C, Vitamin E, Selenium, and Carotenoids*. National Academy Press; 2002:21-34.
33. Sermet-Gaudelus I, Poisson-Salomon AS, Colomb V, et al. Simple pediatric nutritional risk score to identify children at risk of malnutrition. *Am J Clin Nutr*. 2000;72(1):64-70.
34. Joint Food and Agriculture Organization/World Health Organization/United Nations University Expert Consultation. Protein and Amino Acid Requirements in Human Nutrition. *World Health Organ Tech Rep Ser*. 2007;(935):1-265.
35. World Health Organization, World Food Programme, United Nations System Standing Committee on Nutrition, United Nations Children's Fund. *Community-Based Management of Severe Acute Malnutrition*. Geneva, Switzerland: World Health Organization; 2007. https://apps.who.int/iris/handle/10665/44295
36. Israels T, Borgstein E, Jamali M, de Kraker J, Caron HN, Molyneux EM. Acute malnutrition is common in Malawian patients with a Wilms tumour: a role for peanut butter. *Pediatr Blood Cancer*. 2009;53(7):1221-1226.
37. World Health Organization. *Management of Severe Malnutrition: A Manual for Physicians and Other Senior Health Workers*. Geneva, Switzerland: World Health Organization; 1999. https://apps.who.int/iris/bitstream/handle/10665/41999/a57361.pdf?sequence=1
38. Ruiz Garcia V, Lopez-Briz E, Carbonell Sanchis R, Gonzalvez Perales JL, Bort-Marti S. Megestrol acetate for treatment of anorexia-cachexia syndrome. *Cochrane Database Syst Rev*. 2013;3:CD004310.

39. Cuvelier GD, Baker TJ, Peddie EF, et al. A randomized, double-blind, placebo-controlled clinical trial of megestrol acetate as an appetite stimulant in children with weight loss due to cancer and/or cancer therapy. *Pediatr Blood Cancer*. 2014;61(4):672-679.
40. Couluris M, Mayer JL, Freyer DR, Sandler E, Xu P, Krischer JP. The effect of cyproheptadine hydrochloride (periactin) and megestrol acetate (megace) on weight in children with cancer/treatment-related cachexia. *J Pediatr Hematol Oncol*. 2008;30(11):791-797.
41. Bankhead R, Boullata J, Brantley S, et al. Enteral nutrition practice recommendations. *J Parenter Enteral Nutr*. 2009;33(2):122-167.
42. Loser C, Aschl G, Hebuterne X, et al. ESPEN guidelines on artificial enteral nutrition-percutaneous endoscopic gastrostomy (PEG). *Clin Nutr*. 2005;24(5):848-861.
43. Ladas EJ, Arora B, Howard SC, Rogers PC, Mosby TT, Barr RD. A framework for adapted nutritional therapy for children with cancer in low- and middle-income countries: a report from the SIOP PODC Nutrition Working Group. *Pediatr Blood Cancer*. 2016;63(8):1339-1348.
44. Braegger C, Decsi T, Dias JA, et al. Practical approach to paediatric enteral nutrition: a comment by the ESPGHAN committee on nutrition. *J Pediatr Gastroenterol Nutr*. 2010;51(1):110-122.
45. Mathus-Vliegen LM, Binnekade JM, de Haan RJ. Bacterial contamination of ready-to-use 1-L feeding bottles and administration sets in severely compromised intensive care patients. *Crit Care Med*. 2000;28(1):67-73.
46. Mathus-Vliegen EM, Bredius MW, Binnekade JM. Analysis of sites of bacterial contamination in an enteral feeding system. *J Parenter Enteral Nutr*. 2006;30(6):519-525.
47. Simons SR, Abdallah LM. Bedside assessment of enteral tube placement: aligning practice with evidence. *Am J Nurs*. 2012;112(2):40-46; quiz 48, 47.
48. Wilkes-Holmes C. Safe placement of nasogastric tubes in children. *Paediatr Nurs*. 2006;18(9):14-17.
49. Boullata JI, Carrera AL, Harvey L, Escuro AA, Hudson L, Mays A, et al.; ASPEN Safe Practices for Enteral Nutrition Therapy Task Force, American Society for Parenteral and Enteral Nutrition. ASPEN Safe Practices for Enteral Nutrition Therapy. JPEN J Parenter Enteral Nutr. 2017 Jan;41(1):15-103.
50. Fisher C, Blalock BE. Clogged feeding tubes: a clinician's thorn. *Practical Gastro*. 2014;XXXVIII(3):16-22.
51. Viani K. Parenteral and enteral nutrition for paediatric oncology in low and middle income countries. *Indian J Cancer*. 2015;52(2):182-184.
52. Food and Agriculture Organization of the United Nations, World Health Organization, United Nations University. Human energy requirements: report of a joint FAO/WHO/UNU expert consultation. *Food Nutr Bull*. 2005;26(1):166.
53. Grover Z, Ee LC. Protein energy malnutrition. *Pediatr Clin North Am*. 2009;56(5):1055-1068.
54. Moody KM, Baker RA, Santizo RO, et al. A randomized trial of the effectiveness of the neutropenic diet versus food safety guidelines on infection rate in pediatric oncology patients. *Pediatr Blood Cancer*. 2018;65(1)
55. Garcia M, Chismark EA, Mosby T, Day SW. Development and validation of a nutritional education pamphlet for low literacy pediatric oncology caregivers in Central America. *J Cancer Educ*. 2010;25(4):512-517.
56. Mosby TT, Romero AL, Linares AL, Challinor JM, Day SW, Caniza M. Testing efficacy of teaching food safety and identifying variables that affect learning in a low-literacy population. *J Cancer Educ*. 2015;30(1):100-107.
57. Hartman C, Shamir R, Simchowitz V, et al. ESPGHAN/ESPEN/ESPR/CSPEN guidelines on pediatric parenteral nutrition: complications. *Clin Nutr*. 2018;37(6 Pt B):2418-2429.

58. Puntis J, Hojsak I, Ksiazyk J, ESPGHAN/ESPEN/ESPR/CSPEN working group on pediatric parenteral nutrition. ESPGHAN/ESPEN/ESPR/CSPEN guidelines on pediatric parenteral nutrition: organisational aspects. *Clin Nutr*. 2018;37(6 Pt B):2392-2400.
59. European Society for Paediatric Gastroenterology Hepatology and Nutrition. Paediatric parenteral nutrition tool. Updated 2020. Accessed August 8, 2022. https://espghan.info/paediatric-parenteral-nutrition-tool/index.php
60. World Health Organization. *Pocket Book of Hospital Care for Children: Guidelines for the Management of Common Childhood Illnesses*. 2nd ed. Geneva, Switzerland: World Health Organization; 2013.
61. Malawi Ministry of Health. *Guidelines for Community-Based Management of Acute Malnutrition*. Lilongwe, Malawi: Malawi Ministry of Health; 2016. https://www.fantaproject.org/sites/default/files/resources/Malawi-CMAM-Guidelines-Dec2016.pdf
