# Supplementary material for: Consensus recommendations for the nutritional management of children with cancer in limited resource settings: a report from the International Initiative for Pediatrics and Nutrition
Source: Front Nutr. 2025 Jun 26;12:1605632. doi: 10.3389/fnut.2025.1605632 (PMC12240747; doi:10.3389/fnut.2025.1605632)
Supplement: Supplementary file 3 [file Data_Sheet_3.pdf]

IIPAN

COLUMBIA
COLUMBIA UNIVERSITY
IRVING MEDICAL CENTER

IIPAN

INTERNATIONAL INITIATIVE
for PEDIATRICS AND NUTRITION

Appendix for Nutritional Intervention:
A Training Manual for Pediatric Oncology

Second Edition

IIPAN

COLUMBIA
COLUMBIA UNIVERSITY
IRVING MEDICAL CENTER

Contents

Appendix Table 1. Management of Common Nutrition-Related Side-Effects of Cancer Chemotherapy and Radiotherapy	3
Appendix Table 2. Common Laboratory Values Used in Nutritional Assessment	7
Appendix Table 3. Clinical Evidence of Nutritional Deficiencies	10
Appendix Table 4. Estimated Energy Requirement (EER) for Girls and Boys 0 through 35 Months of Age	14
Appendix Table 5. Estimated Energy Requirement (EER) for Girls 3 through 18 Years of Age	15
Appendix Table 6. Estimated Energy Requirement (EER) for Boys 3 through 18 Years of Age	16
Appendix Table 7. Recommended Dietary Allowance (RDA) and Adequate Intake (AI) for Protein	17
Appendix Table 8. Estimated Daily Fluid Requirements	18
Appendix Table 9. Dietary Reference Intakes (DRIs): Recommended Dietary Allowances and Adequate Intakes, Vitamins	19
Appendix Table 10. Dietary Reference Intakes (DRIs): Recommended Dietary Allowances and Adequate Intakes, Elements	20
Appendix Table 11. Dietary Reference Intakes (DRIs): Tolerable Upper Intake Levels, Vitamins	21
Appendix Table 12. Dietary Reference Intakes (DRIs): Tolerable Upper Intake Levels, Elements	22
Appendix Figure 1. Nutritional Intervention Algorithm for Low- and Middle-Income Countries (LMIC)	23
Appendix Table 13. Commonly Used Enteral Formulas	24

Appendix Table 1. Management of Common Nutrition-Related Side-Effects of Cancer Chemotherapy and Radiotherapy

Side effect	Description	Causative agents	What happens if not managed?	Coping strategies
Altered taste	Chemotherapy damage to the taste buds situated in the tongue and oropharynx due to the fact that they have a high turnover	Vincristine, Carmustine, Dacarbazine, Cisplatin, Cyclophosphamide, and antibiotics are among the most common	Tendency to lower oral intake that leads to anorexia and then nutritional depletion<br>Can lead to vomiting<br>Learn aversion to some foods	Good oral hygiene<br>Keep mouth moist at all times<br>Use stronger seasoning on foods and serve food warm<br>Avoid excessively sweet foods<br>Offer salty or sour foods<br>Try new flavors<br>Use different cooking methods<br>Use gravies and sauce to help with swallowing
Anorexia and low intake	Anorexia is described as one of the following: low appetite, early satiety, taste and smell alterations, and meat aversions	Result of enlarged organs pressing on the stomach<br>Shortness of breath<br>Altered taste<br>Constipation<br>Nausea and vomiting<br>Diarrhea<br>Pain and fatigue<br>Metabolic disturbances<br>Depression due to unfamiliar surroundings and continuous medical procedures	Decreased intake of food resulting in weight loss and muscle wasting	Nutritional counseling can lead to increased oral caloric intake<br>Small, frequent meals (6-8 a day)<br>Serve food on a smaller plate<br>Eat and make food more nutrient dense (e.g., add margarine to porridge for breakfast or cheese to mashed potatoes)<br>Present food more attractively<br>Mealtimes in a relaxed, pleasant environment or let children eat together at a table for social interaction<br>Provide nutritional supplements or start with NG feeds
Constipation	Decrease in frequency of stools or hard stools accompanied by pain and discomfort	Vincristine<br>Intestinal obstruction<br>Spinal cord compression<br>Electrolyte imbalance	Pain<br>Low intake leads to weight loss	Prevention is best option<br>Increase fluid and fiber intake<br>Exercise

Side effect	Description	Causative agents	What happens if not managed?	Coping strategies
		Pain medication<br>Lack of exercise<br>Low fiber diet		Absolute privacy for child when going to the toilet or using a bedpan
Diarrhea	Frequent loose stools, abdominal pains, and flatus	Actinomycin, Adriamycin, Methotrexate (*high dose*), and Cytosine are among the most common<br>Result of mucositis, tumor infiltration in GI tract, anxiety, malabsorption, infection, prolonged use of antibiotics, disturbance of the flora of the GI tract, radiotherapy to GI tract	Increased loss of nutrients<br>Decreased oral intake due to fear of increasing diarrhea<br>Weight loss<br>Breakdown of skin and infections	Adequate hydration<br>Good hygiene<br>Keep rectal area clean and dry<br>Clear fluids<br>Elemental or semi-elemental enteral diet<br>NPO or TPN in worse case
Liver damage	Chemotherapy may place added stress on the liver's filtering function<br>Excess calories and lipids in TPN solutions is associated with liver damage	Mercaptopurine, Thioguanine, and Asparaginase are among the most common	Liver damage may lead to kidney failure, excessive fluid in the brain, increase risk of infections and bleeding in the GI tract	Protein, sodium, or fat-controlled diet (if indicated)<br>Supplementation of milk thistle may be beneficial[a]
Nausea and vomiting	The vomiting center is situated in the medulla oblongata near the respiratory center and may be stimulated by:<br>• Chemoreceptor trigger zone<br>• Vagal and visceral nerves	Cytarabine, Methotrexate, Cisplatin, Cyclophosphamide, Doxorubicin, Dactinomycin, and Dacarbazine are among the most common	Dehydration<br>Metabolic abnormalities<br>Children may vomit and lose their appetite - one of the major causes of anorexia because children are afraid to eat and drink for the risk of vomiting again	Optimize antiemetic therapy<br>Small amount of cold food<br>Encourage slow eating<br>Avoid strong odors<br>Drink fluids between meals and not with meals<br>Drink liquids with a straw and covered cup

Side effect	Description	Causative agents	What happens if not managed?	Coping strategies
	• Cerebral cortex and limbic system		Anxiety and stress can lead to food aversion and refusal of treatment	Children to develop strategies like wishful thinking to cope and activities like storytelling
Renal damage	Decreased output due to chemotherapy damage to nephrons	Cisplatin, Cyclophosphamide, and Ifosfamide are among the most common	Loss of protein through kidneys<br>Loss of minerals (Mg, K, Ca, PO_4) through kidneys can lead to Fanconi's renal syndrome<br>Can lead to chronic renal failure<br>Adjustment to chemotherapy drugs and risk of relapse	Protein and electrolyte-controlled diet (if indicated)<br>Supplementation of electrolytes (if indicated)
Stomatitis/ mucositis	Chemotherapy damages the mucosa in the mouth by penetrating the epithelium cells and causing damage to the connective tissue<br>Suppressing of bone marrow and neutropenia	Actinomycin, Adriamycin, Daunorubicin, Epirubicin, Bleomycin, Melphalan, and Methotrexate are among the most common	Leads to inadequate oral intake due to swelling, pain, ulcerations, dry, cracked lips, and then nutritional depletion	BEST is to prevent oral mucositis with good oral hygiene and sucking ice cubes: vasoconstriction takes place in the mouth because of the cold ice cubes and prevents damage by the chemotherapy<br>Optimal pain medication<br>Give liquids or bland, pureed food<br>Add butter, gravy, sauce or salsa dressing to moisten food<br>Avoid highly seasoned food<br>Avoid hard food<br>Enteral nutrition

Abbreviations: NG, nasogastric, GI, gastrointestinal; TPN, total parenteral nutrition; NPO, nothing by mouth; Mg, magnesium; K, potassium; Ca, calcium; PO_4, phosphorus.

Source: Adapted from Rogers PC, Schoeman J. Nutrition Assessment and Intervention. In: Stefan DC, Rodrigues-Galindo C (eds.). *Pediatric Hematology-Oncology in Countries with Limited Resources*. New York, Springer; 2014:91-112.
[a]Ladas EJ, Kroll DJ, Oberlies NH, Cheng B, Ndao DH, Rheingold SR, Kelly KM. A randomized, controlled, double-blind, pilot study of milk thistle for the treatment of hepatotoxicity in childhood acute lymphoblastic leukemia (ALL). Cancer. 2010 Jan 15;116(2):506-13. doi: 10.1002/cncr.24723. PMID: 20014183; PMCID: PMC3542639.

Appendix Table 2. Common Laboratory Values Used in Nutritional Assessment

Indicator	Lab Values	Clinical Interpretation
ALT	9-50 U/L	ALT is increased with liver damage and is used to screen for and /or monitor liver disease. ALT is usually measured concurrently with AST as part of a liver function panel to determine the source of organ damage.
Albumin (half-life 14-21 days)	3.9-5.2 g/dL	Conditions associated with high levels include dehydration, acute infections, and stress from surgery.<br>Conditions associated with low levels of albumin include ascites, burns, glomerulonephritis, liver disease, malabsorption syndromes, inflammation, and malnutrition.
AST	10-37 U/L	AST is indicated when liver disease is suspected (e.g., jaundice, fatigue, loss of appetite, abdominal pain, nausea, vomiting, dark urine, pale colored stools, itching, ascites, mental changes, history of alcohol abuse, suspected acetaminophen overdose, family history of liver disease, exposure to hepatitis viruses).
BUN	3-20 mg/dL	BUN levels may rise in setting of a high-protein diet, steroid use, dehydration, burn injuries, and renal insufficiency/failure.
Calcium	Males:<br>• <12 months: Not established<br>• 1-14 years: 9.6-10.6 mg/dL<br>• 15-16 years: 9.5-10.5 mg/dL<br>• 17-18 years: 9.5-10.4 mg/dL<br>• 19-21 years: 9.3-10.3 mg/dL<br>• ≥22 years: 8.9-10.1 mg/dL<br>Females:<br>• <12 months: Not established<br>• 1-11 years: 9.6-10.6 mg/dL<br>• 12-14 years: 9.5-10.4 mg/dL<br>• 15-18 years: 9.1-10.3 mg/dL<br>• ≥19 years: 8.9-10.1 mg/dL	Too much calcium in your blood can weaken your bones, create kidney stones, and interfere with how your heart and brain work. Hypercalcemia is usually a result of overactive parathyroid glands.<br>Hypocalcemia results when the parathyroid glands are either absent or impaired. Impaired vitamin D synthesis can also cause the condition. Hypocalcemia can often be caused by chronic kidney disease, hyperphosphatemia, severe pancreatitis, chronic liver disease, and hypoalbuminemia.<br>Ionized calcium is calcium in your blood that is not attached to proteins. Corrected calcium can be used by using the following formula: corrected calcium = serum calcium (mg/dL) + [0.8 * (4 - serum albumin (g/dL))].

Indicator	Lab Values	Clinical Interpretation
Creatinine	Adult males: 0.5-1.2 mg/dL<br>Adult females: 0.4-1.1 mg/dL<br>Children (up to 12 years): 0-0.7 mg/dL	Low serum creatinine values are rare; they usually reflect low muscle mass. Low values may also reflect increased GFR.<br>Serum creatinine increases with decreases in GFR (acute kidney injury or chronic kidney disease).
Glucose	Infants: 40-90 mg/dL or 2.2-5.0 mmol/L<br>Children <2 years: 60-100 mg/dL or 3.3-5.5 mmol/L<br>Adults: 74-106 mg/L or 4.1-5.9 mmol/L	Low glucose (hypoglycemia) may result from endogenous or exogenous insulin excess, prolonged starvation, or liver disease.<br>Elevated glucose levels (hyperglycemia) are most often encountered clinically in the setting of diabetes mellitus. It may also occur with pancreatic neoplasms, hyperthyroidism, steroid use, and adrenocortical dysfunction.
Magnesium	Adults: 13-2.1 mEq/L<br>Children: 1.4-1.7 mEq/L<br>Newborns: 1.4-2 mEq/L	Low magnesium levels can be a sign of diabetes, some digestive problems, malnourishment, or long-term (chronic) alcoholism.<br>Increased levels of magnesium may be seen with kidney disease because magnesium is excreted by the kidneys.
Potassium	Adults: 3.5-5.1 mEq/L or mmol/L<br>Children: 3.4-4.7 mEq/L or mmol/L (age dependent)	Low potassium levels (hypokalemia) can cause muscle weakness, lethargy, and irregular heart rate (arrhythmia). Low levels make it hard for the nerves to fire signals. This affects muscle contraction. The most common cause of hypokalemia is the use of diuretics.<br>Conditions associated with high potassium (hyperkalemia) values include acute kidney injury, kidney disease, tissue injury, infection, dehydration, magnesium deficiency, eating disorder, and supplement use. It is a hallmark of Addison's disease.
Pre-albumin (half-life 1-3 days)	1-5 years: 14-30 mg/dL<br>6-9 years: 15-33 mg/dL<br>10-13 years: 22-36 mg/dL<br>14-19 years: 22-45 mg/dL	Low pre-albumin scores may indicate poor nutrition, liver disease, or inflammation.<br>High pre-albumin scores may be a sign of long-term chronic kidney disease, steroid use, or alcoholism.

Indicator	Lab Values	Clinical Interpretation
Sodium	137-145 mmol/L	Severe vomiting or diarrhea, diuretic use, drinking too much water, kidney disease and liver disease can cause hyponatremia.<br><br>Hypernatremia involves dehydration, which can have many causes, including not drinking enough fluids, diarrhea, kidney dysfunction, and diuretics.

Abbreviations: ALT, alanine aminotransferase; U, unit; L, liter; AST, aspartate aminotransferase; dL, deciliter; BUN, blood urea nitrogen; g, gram; mg, milligram; GFR, glomerular filtration rate; mEq, milliequivalents; mmol, millimole.

Appendix Table 3. Clinical Evidence of Nutritional Deficiencies

Nutrient	Effects of deficiency	Clinical findings
Biotin	Seborrheic dermatitis, alopecia, glossitis, irritability, lethargy	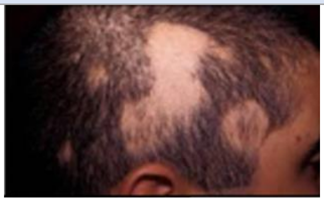
Copper	Poor growth, depigmentation of hair, brittle hair, pallor, microcytic anemia, neutropenia, osteoporosis, fractures, hypotonia	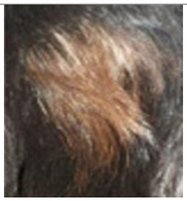
Cyanocobalamin (B12)	Glossitis, cheilosis, megaloblastic anemia, peripheral neuropathy	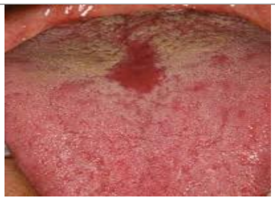
Essential fatty acids	Desquamating dermatitis, alopecia, poor growth, hepatomegaly	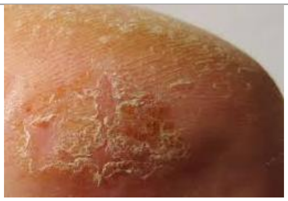
Folate	Glossitis, megaloblastic anemia	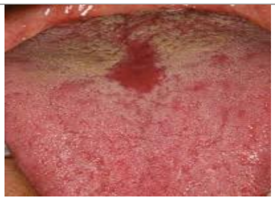
Iodine	Thyroid enlargement (goiter)	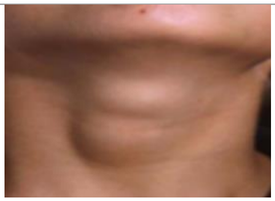

Nutrient	Effects of deficiency	Clinical findings
Iron	Fatigue, headache, koilonychia (spoon nails), glossitis, pallor, tachycardia, anemia, decreased mental performance	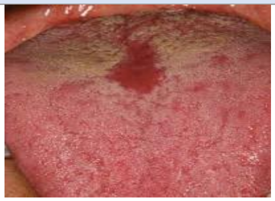
Niacin	Symmetric dermatitis in light-exposed areas or on pressure (Pellagra), cheilosis, glossitis, diarrhea, apathy	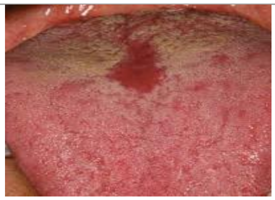 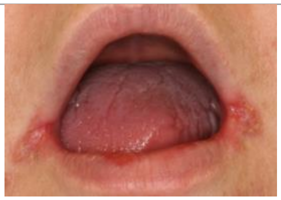 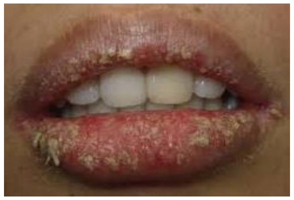
Protein	Easily pulled out hair, transverse ridging of nails, pitting edema	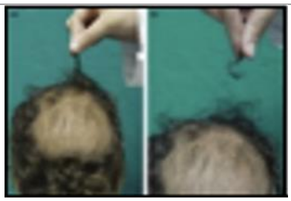 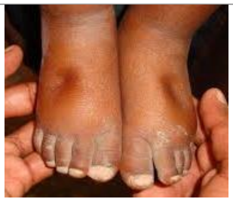 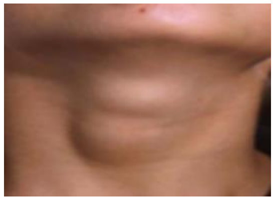 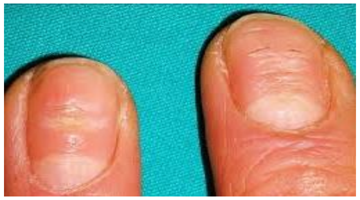
Protein-energy	Poor growth, decreased subcutaneous fat, muscle wasting, edema, bilateral pitting, fine, dull, sparse hair, dry, scaling, flaky skin, altered skin pigmentation, atrophic lingual papillae, angular stomatitis, cheilosis, ridging of nails, abdominal distension, hepatomegaly	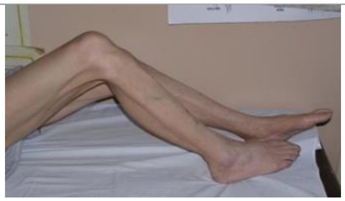

Nutrient	Effects of deficiency	Clinical findings
Pyridoxine (B6)	Seborrheic dermatitis, glossitis, angular stomatitis, cheilosis, seizures, peripheral neuropathy, microcytic anemia	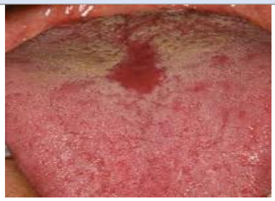 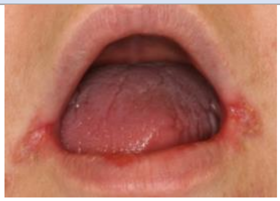 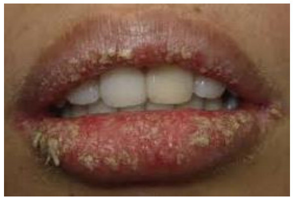
Riboflavin (B2)	Angular stomatitis, cheilosis, glossitis, seborrheic dermatitis, normocytic anemia	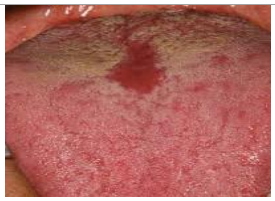 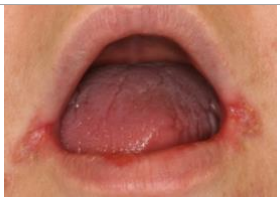 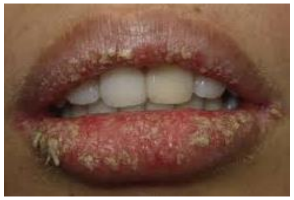
Selenium	Poor growth, cardiomyopathy, osteoarthropathy	
Thiamin (B1)	Ophthalmoplegia, calf tenderness, hyporeflexia, muscle weakness, ataxia, tachycardia, edema, irritability, apathy, restlessness	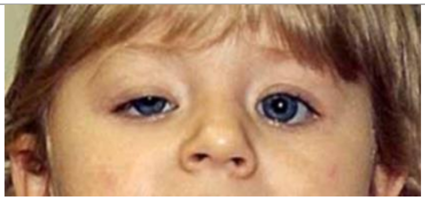
Vitamin A	Night blindness, xerophthalmia (xerosis of conjunctivae and cornea, Bitot's spots, corneal ulcerations), follicular hyperkeratosis, corkscrew hairs, poor growth	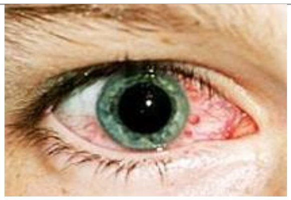 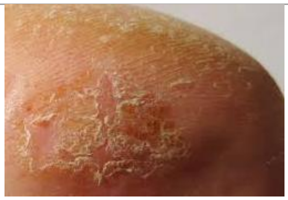

Nutrient	Effects of deficiency	Clinical findings
Vitamin C (ascorbic acid)	Follicular hyperkeratosis, corkscrew hairs, gingival hypertrophy and bleeding, perifollicular petechiae, ecchymoses, hemarthroses, periosteal hemorrhage, anemia, bone tenderness, costochondral beading, poor wound healing, irritability, fatigue	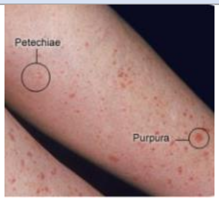
Vitamin D	Poor growth, craniotabes, enlarged fontanel, epiphyseal enlargement, knock-knees or bowing of extremities, costochondral beading, tetany	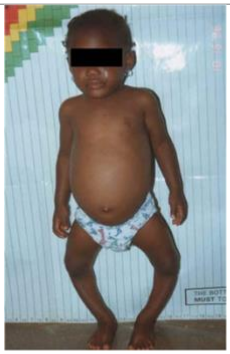
Vitamin E	Hemolytic anemia, edema, ataxia, hyporeflexia, ophthalmoplegia, hypotonia	
Vitamin K	Petechiae, purpura, ecchymoses, bleeding diathesis	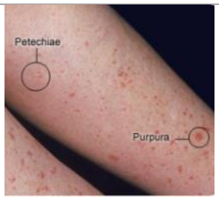
Zinc	Periorificial dermatitis, perianal rash, poor growth, angular stomatitis, alopecia, hypogonadism, delayed puberty, poor wound healing, hepatosplenomegaly, diarrhea, apathy, impaired taste sensation	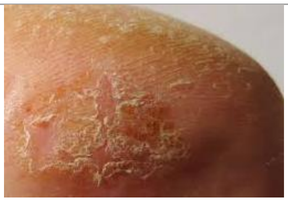

Source: Adapted from Ladas EJ, Arora B, Howard SC, Rogers PC, Mosby TT, Barr RD. A framework for adapted nutritional therapy for children with cancer in low- and middle-income countries: a report from the SIOP PODC Nutrition Working Group. *Pediatr Blood Cancer*. 2016;63(8):1339-1348.

Appendix Table 4. Estimated Energy Requirement (EER) for Girls and Boys 0 through 35 Months of Age

Estimated Energy Requirement (kcal/day)[a]		
Age	Girls	Boys
1 month	438	472
2 months	500	567
3 months	521	572
4 months	508	548
5 months	553	596
6 months	593	645
7 months	608	668
8 months	643	710
9 months	678	746
10 months	717	793
11 months	742	817
12-14 months	768	844
15-17 months	837	908
18-20 months	899	961
21-23 months	952	1006
24-26 months	997	1050
27-29 months	1033	1086
30-32 months	1077	1121
33-34 months	1113	1157
35 months	1139	1184

Abbreviations: kcal, calories.
[a]EER is equal to total energy expenditure (TEE) + energy deposition (ED).
Source: Institute of Medicine. *Dietary Reference Intakes for Energy, Carbohydrate, Fiber, Fat, Fatty Acids, Cholesterol, Protein, and Amino Acids.* Washington, DC: The National Academies Press; 2005. https://doi.org/10.17226/10490.

Appendix Table 5. Estimated Energy Requirement (EER) for Girls 3 through 18 Years of Age

Estimated Energy Requirement for Girls 3 through 18 Years of Age						
Age	Reference weight (kg)	Reference height (m)	Sedentary[a] (kcal/day)	Low active[b] (kcal/day)	Active[c] (kcal/day)	Very active[d] (kcal/day)
3 years	13.9	0.94	1080	1243	1395	1649
4 years	15.8	1.01	1133	1310	1475	1750
5 years	17.9	1.08	1189	1379	1557	1854
6 years	20.2	1.15	1247	1451	1642	1961
7 years	22.8	1.21	1298	1515	1719	2058
8 years	25.6	1.28	1360	1593	1810	2173
9 years	29.0	1.33	1415	1660	1890	2273
10 years	32.9	1.38	1470	1729	1972	2376
11 years	37.2	1.44	1538	1813	2071	2500
12 years	41.6	1.51	1617	1909	2183	2640
13 years	45.8	1.57	1684	1992	2281	2762
14 years	49.4	1.60	1718	2036	2334	2831
15 years	52.0	1.62	1731	2057	2362	2870
16 years	53.9	1.63	1729	2059	2368	2883
17 years	55.1	1.63	1710	2042	2353	2871
18 years	56.2	1.63	1690	2024	2336	2858

Abbreviations: kg, kilogram; m, meter; kcal, calories.
[a]Sedentary: Typical daily living activities (e.g., household tasks, walking to the bus)
[b]Low active: Typical daily living activities PLUS 30-60 minutes of daily moderate activity (e.g., walking at 5-7 km/h)
[c]Active: Typical daily living activities PLUS at least 60 minutes of daily moderate activity
[d]Very active: Typical daily living activities PLUS at least 60 minutes of daily moderate activity PLUS an additional 60 minutes of vigorous activity or 120 minutes of moderate activity
Source: Institute of Medicine. *Dietary Reference Intakes for Energy, Carbohydrate, Fiber, Fat, Fatty Acids, Cholesterol, Protein, and Amino Acids*. Washington, DC: The National Academies Press; 2005. https://doi.org/10.17226/10490.

Appendix Table 6. Estimated Energy Requirement (EER) for Boys 3 through 18 Years of Age

Estimated Energy Requirement for Boys 3 through 18 Years of Age						
Age	Reference weight (kg)	Reference height (m)	Sedentary[a] (kcal/day)	Low active[b] (kcal/day)	Active[c] (kcal/day)	Very active[d] (kcal/day)
3 years	14.3	0.95	1162	1324	1485	1683
4 years	16.2	1.02	1215	1390	1566	1783
5 years	18.4	1.09	1275	1466	1658	1894
6 years	20.7	1.15	1328	1535	1742	1997
7 years	23.1	1.22	1393	1617	1840	2115
8 years	25.6	1.28	1453	1692	1931	2225
9 years	28.6	1.34	1530	1787	2043	2359
10 years	31.9	1.39	1601	1875	2149	2486
11 years	35.9	1.44	1691	1985	2279	2640
12 years	40.5	1.49	1798	2113	2428	2817
13 years	45.6	1.56	1935	2276	2618	3038
14 years	51.0	1.64	2090	2459	2829	3283
15 years	56.3	1.70	2223	2618	3013	3499
16 years	60.9	1.74	2320	2736	3152	3663
17 years	64.6	1.75	2366	2796	3226	3754
18 years	67.2	1.76	2383	2823	3263	3804

Abbreviations: kg, kilogram; m, meter; kcal, calories.
[a]Sedentary: Typical daily living activities (e.g., household tasks, walking to the bus)
[b]Low active: Typical daily living activities PLUS 30-60 minutes of daily moderate activity (e.g., walking at 5-7 km/h)
[c]Active: Typical daily living activities PLUS at least 60 minutes of daily moderate activity
[d]Very active: Typical daily living activities PLUS at least 60 minutes of daily moderate activity PLUS an additional 60 minutes of vigorous activity or 120 minutes of moderate activity
Source: Institute of Medicine. *Dietary Reference Intakes for Energy, Carbohydrate, Fiber, Fat, Fatty Acids, Cholesterol, Protein, and Amino Acids*. Washington, DC: The National Academies Press; 2005. https://doi.org/10.17226/10490.

Appendix Table 7. Recommended Dietary Allowance (RDA) and Adequate Intake (AI) for Protein

RDA or AI for Protein (g/day)		
Age	Girls	Boys
0-6 months	9.1	
7-12 months	11	
1-3 years	13	
4-8 years	19	
9-13 years	34	
14-18 years	46	52

Abbreviations: RDA, Recommended Dietary Allowance; AI, Adequate Intake; g, gram.
Source: Institute of Medicine. *Dietary Reference Intakes for Energy, Carbohydrate, Fiber, Fat, Fatty Acids, Cholesterol, Protein, and Amino Acids.* Washington, DC: The National Academies Press; 2005. https://doi.org/10.17226/1.

Appendix Table 8. Estimated Daily Fluid Requirements

Weight (kg)	Daily fluid requirements (mL)	Weight (kg)	Daily fluid requirements (mL)	Weight (kg)	Daily fluid requirements (mL)
1	100	32	1740	72	2540
2	200	34	1780	74	2580
3	300	36	1820	76	2620
4	400	38	1860	78	2660
5	500	40	1900	80	2700
6	600	42	1940	82	2740
7	700	44	1980	84	2780
8	800	46	2020	86	2820
9	900	48	2060	88	2860
10	1000	50	2100	90	2900
12	1100	52	2140	92	2940
14	1200	54	2180	94	2980
16	1300	56	2220	96	3020
18	1400	58	2260	98	3060
20	1500	60	2300	100	3100
22	1540	62	2340	102	3140
24	1580	64	2380	104	3180
26	1620	66	2420	106	3220
28	1660	68	2460	108	3260
30	1700	70	2500	110	3300

Abbreviation: kg, kilogram; mL, milliliter.

Appendix Table 9. Dietary Reference Intakes (DRIs): Recommended Dietary Allowances and Adequate Intakes, Vitamins

Life stage group	Vitamin A (µg/d)	Vitamin C (mg/d)	Vitamin D (µg/d)	Vitamin E (mg/d)	Vitamin K (µg/d)	Thiamin (mg/d)	Riboflavin (mg/d)	Niacin (mg/d)	Vitamin B_6 (mg/d)	Folate (µg/d)	Vitamin B_{12} (µg/d)	Pantothenic acid (mg/d)	Biotin (µg/d)	Choline (mg/d)
Infants														
0-6 mo	400*	40*	10*	4*	2.0*	0.2*	0.3*	2*	0.1*	65*	0.4*	1.7*	5*	125*
7-12 mo	500*	50*	10*	5*	2.5*	0.3*	0.4*	4*	0.3*	80*	0.5*	1.8*	6*	150*
Children														
1-3 y	300	15	15	6	30*	0.5	0.5	6	0.5	150	0.9	2*	8*	200*
4-8 y	400	25	15	7	55*	0.6	0.6	8	0.6	200	1.2	3*	12*	250*
Males														
9-13 y	600	45	15	11	60*	0.9	0.9	12	1.0	300	1.8	4*	20*	375*
14-18 y	900	75	15	15	75*	1.2	1.3	16	1.3	400	2.4	5*	25*	550*
Females														
9-13 y	600	45	15	11	60*	0.9	0.9	12	1.0	300	1.8	4*	20*	375*
14-18 y	700	65	15	15	75*	1.0	1.0	14	1.2	400	2.4	5*	25*	400*
Pregnancy														
14-18 y	750	80	15	15	75*	1.4	1.4	18	1.9	600	2.6	6*	30*	450*
Lactation														
14-18 y	1,200	115	15	19	75*	1.4	1.6	17	2.0	500	2.8	7*	35*	550*

Abbreviations: µg, microgram; d, day; mg, milligram; mo, months; y, years. *Adequate Intakes are followed by an asterisk (*).
Sources: Dietary Reference Intakes for Calcium, Phosphorous, Magnesium, Vitamin D, and Fluoride (1997); Dietary Reference Intakes for Thiamin, Riboflavin, Niacin, Vitamin B6, Folate, Vitamin B12, Pantothenic Acid, Biotin, and Choline (1998); Dietary Reference Intakes for Vitamin C, Vitamin E, Selenium, and Carotenoids (2000); Dietary Reference Intakes for Vitamin A, Vitamin K, Arsenic, Boron, Chromium, Copper, Iodine, Iron, Manganese, Molybdenum, Nickel, Silicon, Vanadium, and Zinc (2001); Dietary Reference Intakes for Energy, Carbohydrate, Fiber, Fat, Fatty Acids, Cholesterol, Protein, and Amino Acids (2002/2005); and Dietary Reference Intakes for Calcium and Vitamin D (2011).

Appendix Table 10. Dietary Reference Intakes (DRIs): Recommended Dietary Allowances and Adequate Intakes, Elements

Life Stage Group	Ca (mg/d)	Cr (µg/d)	Copper (µg/d)	Fl (mg/d)	Iodine (µg/d)	Iron (mg/d)	Mg (mg/d)	Mn (mg/d)	Mo (µg/d)	Phos (mg/d)	Se (µg/d)	Zinc (mg/d)	K (mg/d)	Na (mg/d)	Cl (g/d)
Infants															
0-6 mo	200*	0.2*	200*	0.01*	110*	0.27*	30*	0.003*	2*	100*	15*	2*	400*	110*	0.18*
7-12 mo	260*	5.5*	220*	0.5*	130*	11	75*	0.6*	3*	275*	20*	3	860*	370*	0.57*
Children															
1-3 y	700	11*	340	0.7*	90	7	80	1.2*	17	460	20	3	2,000*	800*	1.5*
4-8 y	1,000	15*	440	1*	90	10	130	1.5*	22	500	30	5	2,300*	1,000*	1.9*
Males															
9-13 y	1,300	25*	700	2*	120	8	240	1.9*	34	1,250	40	8	2,500*	1,200*	2.3*
14-18 y	1,300	35*	890	3*	150	11	410	2.2*	43	1,250	55	11	3,000*	1,500*	2.3*
Females															
9-13 y	1,300	21*	700	2*	120	8	240	1.6*	34	1,250	40	8	2,300*	1,200*	2.3*
14-18 y	1,300	24*	890	3*	150	15	360	1.6*	43	1,250	55	9	2,300*	1,500*	2.3*
Pregnancy															
14-18 y	1,300	29*	1,000	3*	220	27	400	2.0*	50	1,250	60	12	2,600*	1,500*	2.3*
Lactation															
14-18 y	1,300	44*	1,300	3*	290	10	360	2.6*	50	1,250	70	13	2,500*	1,500*	2.3*

Abbreviations: Ca, calcium; mg, milligram; d, day; Cr, chromium; µg, microgram; Fl, fluoride; Mg, magnesium; Mn, manganese; Mo, molybdenum; Phos, phosphorus; Se, selenium; K, potassium; Na, sodium; Cl, chloride; g, gram; mo, months; y, years. *Adequate Intakes are followed by an asterisk (*).

Sources: Dietary Reference Intakes for Calcium, Phosphorous, Magnesium, Vitamin D, and Fluoride (1997); Dietary Reference Intakes for Thiamin, Riboflavin, Niacin, Vitamin B6, Folate, Vitamin B12, Pantothenic Acid, Biotin, and Choline (1998); Dietary Reference Intakes for Vitamin C, Vitamin E, Selenium, and Carotenoids (2000); Dietary Reference Intakes for Vitamin A, Vitamin K, Arsenic, Boron, Chromium, Copper, Iodine, Iron, Manganese, Molybdenum, Nickel, Silicon, Vanadium, and Zinc (2001); Dietary Reference Intakes for Energy, Carbohydrate, Fiber, Fat, Fatty Acids, Cholesterol, Protein, and Amino Acids (2002/2005); and Dietary Reference Intakes for Calcium and Vitamin D (2011).

Appendix Table 11. Dietary Reference Intakes (DRIs): Tolerable Upper Intake Levels, Vitamins

Life stage group	Vitamin A (µg/d)	Vitamin C (mg/d)	Vitamin D (µg/d)	Vitamin E (mg/d)	Vitamin K (µg/d)	Thiamin (mg/d)	Riboflavin (mg/d)	Niacin (mg/d)	Vitamin B6 (mg/d)	Folate (µg/d)	Vitamin B12 (µg/d)	Pantothenic acid (mg/d)	Biotin (µg/d)	Choline (g/d)
Infants														
0-6 mo	600	ND	25	ND	ND	ND	ND	ND	ND	ND	ND	ND	ND	ND
7-12 mo	600	ND	38	ND	ND	ND	ND	ND	ND	ND	ND	ND	ND	ND
Children														
1-3 y	600	400	63	200	ND	ND	ND	10	30	300	ND	ND	ND	1.0
4-8 y	900	650	75	300	ND	ND	ND	15	40	400	ND	ND	ND	1.0
Males														
9-13 y	1,700	1,200	100	600	ND	ND	ND	20	60	600	ND	ND	ND	2.0
14-18 y	2,800	1,800	100	800	ND	ND	ND	30	80	800	ND	ND	ND	3.0
Females														
9-13 y	1,700	1,200	100	600	ND	ND	ND	20	60	600	ND	ND	ND	2.0
14-18 y	2,800	1,800	100	800	ND	ND	ND	30	80	800	ND	ND	ND	3.0
Pregnancy														
14-18 y	2,800	1,800	100	800	ND	ND	ND	30	80	800	ND	ND	ND	3.0
Lactation														
14-18 y	2,800	1,800	100	800	ND	ND	ND	30	80	800	ND	ND	ND	3.0

Abbreviations: µg, microgram; d, day; mg, milligram; mo, months; y, years; ND, not determinable.
Sources: Dietary Reference Intakes for Calcium, Phosphorous, Magnesium, Vitamin D, and Fluoride (1997); Dietary Reference Intakes for Thiamin, Riboflavin, Niacin, Vitamin B6, Folate, Vitamin B12, Pantothenic Acid, Biotin, and Choline (1998); Dietary Reference Intakes for Vitamin C, Vitamin E, Selenium, and Carotenoids (2000); Dietary Reference Intakes for Vitamin A, Vitamin K, Arsenic, Boron, Chromium, Copper, Iodine, Iron, Manganese, Molybdenum, Nickel, Silicon, Vanadium, and Zinc (2001); Dietary Reference Intakes for Energy, Carbohydrate, Fiber, Fat, Fatty Acids, Cholesterol, Protein, and Amino Acids (2002/2005); and Dietary Reference Intakes for Calcium and Vitamin D (2011).

Appendix Table 12. Dietary Reference Intakes (DRIs): Tolerable Upper Intake Levels, Elements

Life Stage Group	Boron (mg/d)	Ca (mg/d)	Cr (µg/d)	Copper (µg/d)	Fl (mg/d)	Iodine (µg/d)	Iron (mg/d)	Mg[a] (mg/d)	Mn (mg/d)	Mo (µg/d)	Phos (g/d)	Se (µg/d)	Zinc (mg/d)	K (mg/d)	Na (mg/d)	Cl (g/d)	Nickel (mg/d)
Infants																	
0-6 mo	ND	1,000	ND	ND	0.7	ND	40	ND	ND	ND	ND	45	4	ND	ND	ND	ND
7-12 mo	ND	1,500	ND	ND	0.9	ND	40	ND	ND	ND	ND	60	5	ND	ND	ND	ND
Children																	
1-3 y	3	2,500	ND	1,000	1.3	200	40	65	2	300	3	90	7	ND	ND	2.3	0.2
4-8 y	6	2,500	ND	3,000	2.2	300	40	110	3	600	3	150	12	ND	ND	2.9	0.3
Males																	
9-13 y	11	3,000	ND	5,000	10	600	40	350	6	1,100	4	280	23	ND	ND	3.4	0.6
14-18 y	17	3,000	ND	8,000	10	900	45	350	9	1,700	4	400	34	ND	ND	3.6	1.0
Females																	
9-13 y	11	3,000	ND	5,000	10	600	40	350	6	1,100	4	280	23	ND	ND	3.4	0.6
14-18 y	17	3,000	ND	8,000	10	900	45	350	9	1,700	4	400	34	ND	ND	3.6	1.0
Pregnancy																	
14-18 y	17	3,000	ND	8,000	10	900	45	350	9	1,700	3.5	400	34	ND	ND	3.6	1.0
Lactation																	
14-18 y	17	3,000	ND	8,000	10	900	45	350	9	1,700	4	400	34	ND	ND	3.6	1.0

Abbreviations: mg, milligram; d, day; Ca, calcium; Cr, chromium; µg, microgram; Fl, fluoride; Mg, magnesium; Mn, manganese; Mo, molybdenum; Phos, phosphorus; g, gram; Se, selenium; K, potassium; Na, sodium; Cl, chloride; mo, months; y, years; ND, not determinable. [a]The ULs for magnesium represent intake from a pharmacological agent only and do not include intake from food and water.
Sources: Dietary Reference Intakes for Calcium, Phosphorous, Magnesium, Vitamin D, and Fluoride (1997); Dietary Reference Intakes for Thiamin, Riboflavin, Niacin, Vitamin B6, Folate, Vitamin B12, Pantothenic Acid, Biotin, and Choline (1998); Dietary Reference Intakes for Vitamin C, Vitamin E, Selenium, and Carotenoids (2000); Dietary Reference Intakes for Vitamin A, Vitamin K, Arsenic, Boron, Chromium, Copper, Iodine, Iron, Manganese, Molybdenum, Nickel, Silicon, Vanadium, and Zinc (2001); Dietary Reference Intakes for Energy, Carbohydrate, Fiber, Fat, Fatty Acids, Cholesterol, Protein, and Amino Acids (2002/2005); and Dietary Reference Intakes for Calcium and Vitamin D (2011).

Appendix Figure 1. Nutritional Intervention Algorithm for Low- and Middle-Income Countries (LMIC)

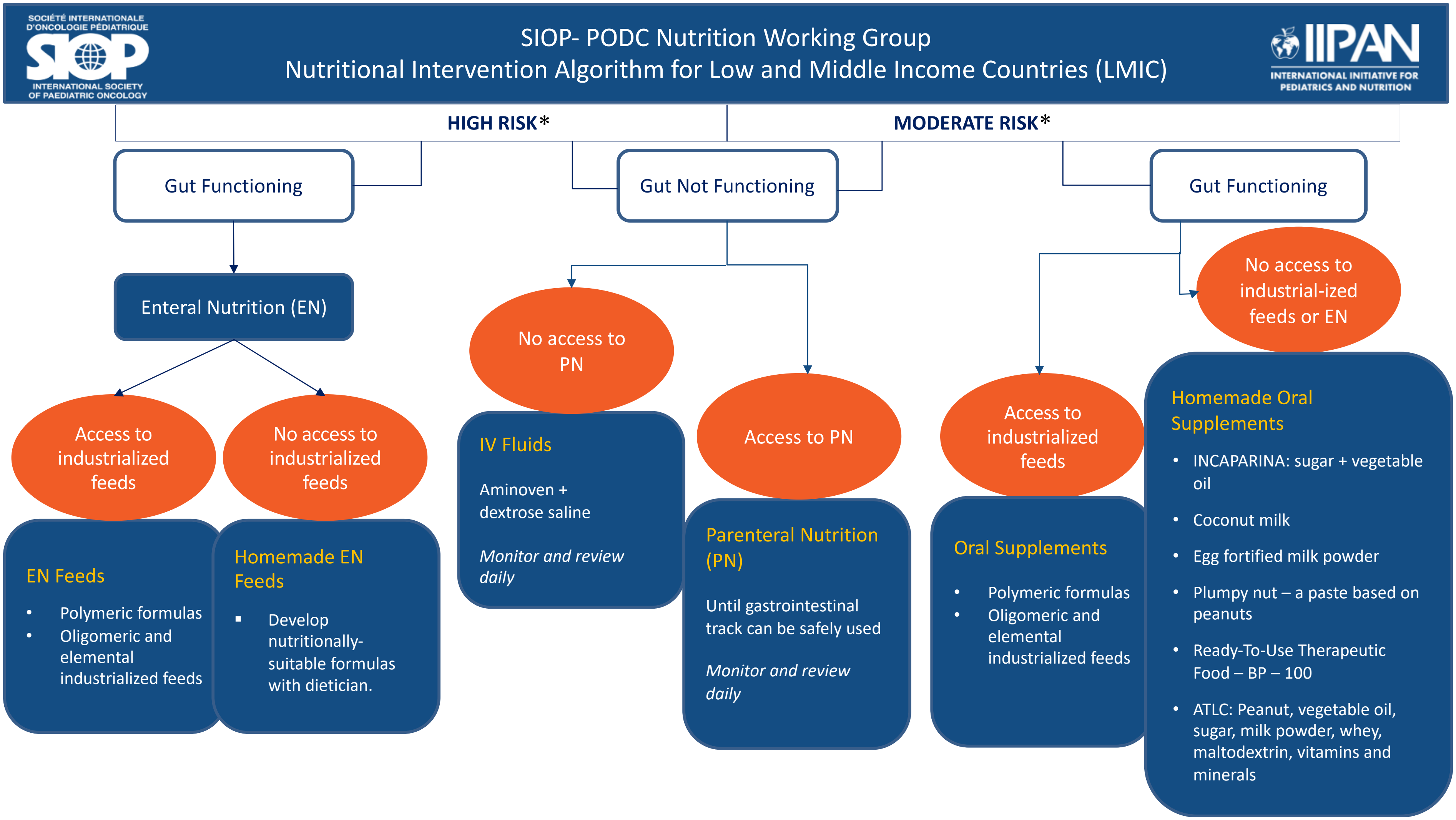

Abbreviations: EN, enteral nutrition; PN, parenteral nutrition; IV, intravenous.
*The definitions of high and moderate nutritional risk for children and adolescents with cancer are not clearly established. Factors that indicate nutritional risk include acute malnutrition, micronutrient deficiencies, dietary intake below 50% of nutritional needs, the presence of a high-risk malignancy, and gastrointestinal symptoms such as vomiting and diarrhea. Additionally, gastrointestinal complications like obstructions, pancreatitis, and typhlitis should also be considered as significant nutritional risk factors.

Appendix Table 13. Commonly Used Enteral Formulas

Formula	Description		Indication	
Polymeric formulas	Contains whole, intact proteins.<br>Typically made of milk and/or soy proteins with various carbohydrate and fat sources.<br>Range in calorie concentration from 100 kcal/100 mL to 150 kcal/100 mL.		First choice for any patient without allergies to soy protein or cow's milk and without severe malabsorptive disorders.<br>Higher calorie concentration formulas (150 kcal/100 mL) may be considered in patients who are having difficulty tolerating higher volumes.	
Examples	Age	Calories	Protein	Fiber
PediaSure® (Abbott)	Pediatric	42 kcal/9.2 g (1 scoop)	1.3 g/9.2 g (1 scoop)	0 g/9.2 g (1 scoop)
PediaSure® (Abbott)	Pediatric	100 kcal/100 mL	3.0 g/100 mL	0 g/100 mL
Nutren Junior® (Nestle)	Pediatric	100 kcal/100 mL	3.0 g/100 mL	0 g/100 mL
Trophic Infant® (Prodiet)	Pediatric	100 kcal/100 mL	3.1 g/100 mL	0 g/100 mL
Boost® Kid Essentials (Nestle)	Pediatric	100 kcal/100 mL	3.0 g/100 mL	0 g/100 mL
Fortini™ Plus (Danone)	Pediatric	150 kcal/100 mL	3.4 g/100 mL	0 g/100 mL
Frebini® Energy Drink (Fresenius Kabi)	Pediatric	150 kcal/100 mL	3.8 g/100 mL	0 g/100 mL
Sustagen® Hospital Formula (Nestle)	Adult	94 kcal/100 mL	5.8 g/100 mL	0 g/100 mL
Fresubin® Original (Fresenius Kabi)	Adult	100 kcal/100 mL	3.8 g/100 mL	0 g/100 mL
Energyzip® (Prodiet)	Adult	100 kcal/100 mL	3.8 g/100 mL	0 g/100 mL
NutriComp® D (Braun)	Adult	103 kcal/100 mL	4.1 g/100 mL	2.1 g/100 mL
NutriComp® Soup (Braun)	Adult	150 kcal/100 mL	6 g/100 mL	2 g/100 mL
Fresubin® Energy Drink (Fresenius Kabi)	Adult	150 kcal/100 mL	5.6 g/100 mL	0 g/100 mL
Nutren® 1.5 (Nestle)	Adult	150 kcal/100 mL	6.8 g/100 mL	0 g/100 mL
Complan® Original (Nutricia)	Adult	61 kcal/15 g (1 rounded tbsp)	2.1 g/15 g (1 rounded tbsp)	0 g/15 g (1 rounded tbsp)
Polymeric formulas with fiber	Contains whole, intact protein and fiber from various sources.		Intended to help maintain healthy gastrointestinal flora and regulate frequency/consistency of stool.	
Examples	Age	Calories	Protein	Fiber

Ensoy Ninos Defense® (Abbott)	Pediatric	70 kcal/15 g (1 scoop)	2.3 g/15 g (1 scoop)	1 g/15 g (1 scoop)
Enterex® Kidz Ultra (Victus)	Pediatric	115 kcal/25 g (1 scoop)	3.5 g/25 g (1 scoop)	1 g/25 g (1 scoop)
Fortini™ Complete (Danone)	Pediatric	99 kcal/100 mL	2.9 g/100 mL	1.0 g/100 mL
Nutren® Junior with Fiber (Nestle)	Pediatric	100 kcal/100 mL	3 g/100 mL	0.6 g/100 mL
FontActiv® Junior (Grand Fontaine)	Pediatric	111 kcal/100 mL	2.6 g/100 mL	0.6 g/100 mL
Fresubin® Original Fibre (Fresenius Kabi)	Adult	100 kcal/100 mL	3.8 g/100 mL	1.5 g/100 mL
Ensure® (Abbott)	Adult	106 kcal/100 mL	3.7 g/100 mL	0 g/100 mL
FontActiv® Complete (Grand Fontaine)	Adult	108 kcal/100 mL	3.9 g/100 mL	1.3 g/100 mL
Supportan® Drink (Fresenius Kabi)	Adult	150 kcal/100 mL	10 g/100 mL	1.5 g/100 mL
Ensure® (Abbott)	Adult	39 kcal/8.9 g (1 scoop)	1.4 g/8.9 g (1 scoop)	0.4 g/8.9 g (1 scoop)
Semi-elemental formulas (also referred to as oligomeric or peptide-based)	Contain proteins that have been hydrolyzed to produce peptides of varying lengths and a small amount of free amino acids.<br>Frequently contain a portion of fat as medium chain triglycerides, which has favorable intestinal absorption.<br>Higher osmolality (300-500 mOsmol/L) than polymeric formulas.<br>More expensive than polymeric formulas.		Intended for use among patients with malabsorptive disorders (the presence of di- and tripeptides enhances nitrogen absorption in the intestines).<br>May be useful in patients with radiation enteritis.	
Examples	Age	Calories	Protein	Fiber
Peptamen Junior® (Nestle)	Pediatric	100 kcal/100 mL	3.0 g/100 mL	0 g/100 mL
Pentasure® Critipep (Hexagon Nutrition)	Adult	99 kcal/20 g (1 scoop)	4 g/20 g (1 scoop)	0.3 g/20 g (1 scoop)
AlitraQ® (Abbott)	Adult	100 kcal/100 mL	5.3 g/100 mL	0 g/100 mL
Perative® (Abbott)	Adult	130 kcal/100 mL	6.7 g/100 mL	0 g/100 mL
Survimed® OPD HN (Fresenius Kabi)	Adult	133 kcal/100 mL	6.7 g/100 mL	0 g/100 mL
Elemental formulas (also referred to as monomeric)	Contain free amino acids.		Intended for use among patients with malabsorptive disorders or among patients with allergy to dairy or soy.<br>Osmolality may contribute to osmotic diarrhea.	

	Frequently contain a portion of fat as medium chain triglycerides, which has favorable intestinal absorption.<br>Highest osmolality (500–900 mOsmol/L) compared to polymeric and semi-elemental formulas.<br>More expensive than polymeric formulas and semi-elemental formulas.			
Examples	Age	Calories	Protein	Fiber
Nutrilon/Aptamil® Pepti-Junor (Nutricia)	Infant (6 mo+)	22 kcal/4.3 g (1 scoop)	0.6 g/4.3 g (1 scoop)	0 g/4.3 g (1 scoop)
PurAmino™ Junior (Mead Johnson)	Pediatric	33 kcal/6.8 g (1 scoop)	1.1 g/6.8 g (1 scoop)	0 g/6.8 g (1 scoop)
Therapeutic formulas and RUTF	F75 and F100 are formulas used for the management of children with severe acute malnutrition.<br>RUTF can be lipid-based pastes or in the form of biscuits.		Intended for use among children with severe acute malnutrition.	
Examples	Age	Calories	Protein	Fiber
F-75	Pediatric/Adult	75 kcal/100 mL	0.9 g/100 mL	Not reported
F-100	Pediatric/Adult	100 kcal/100 mL	2.9 g/100 mL	Not reported
RUTF	Pediatric/Adult	500 kcal/92 g sachet	12.8 g/92 g sachet	<5%

Abbreviations: kcal, calories; mL, milliliter; g, gram; tbsp, tablespoon; mo, months; RUTF, ready-to-use therapeutic foods.
Sources: Brown B, Roehl K, Betz M. Enteral nutrition formula selection: current evidence and implications for practice. *Nutr Clin Pract*. 2015;30(1):72-85.; Zadák Z, Kent-Smith L. Basics in clinical nutrition: commercially prepared formulas. *E Spen Eur E J Clin Nutr Metab*. 2009;4(5):e212-5.; Sanz-Paris A, Martinez-García M, Martinez-Trufero J, et al. Oligomeric enteral nutrition in undernutrition, due to oncology treatment-related diarrhea: systematic review and proposal of an algorithm of action. *Nutrients*. 2019;11(8):1888.; Schoonees A, Lombard M, Musekiwa A, Nel E, Volmink J. Ready-to-use therapeutic food for home-based treatment of severe acute malnutrition in children from six months to five years of age. *Cochrane Database Syst Rev*. 2013;2013(6):CD009000.; World Health Organization. *WHO Guideline: Updates on the management of severe acute malnutrition in infants and children.* Geneva, Switzerland: World Health Organization; 2013.
